# Designing Antitrypanosomal and Antileishmanial BODIPY Derivatives: A Computational and In Vitro Assessment

**DOI:** 10.3390/molecules29092072

**Published:** 2024-04-30

**Authors:** Raquel C. R. Gonçalves, Filipe Teixeira, Pablo Peñalver, Susana P. G. Costa, Juan C. Morales, M. Manuela M. Raposo

**Affiliations:** 1 Centre of Chemistry, University of Minho, Campus de Gualtar, 4710-057 Braga, Portugalfteixeira@quimica.uminho.pt (F.T.); spc@quimica.uminho.pt (S.P.G.C.); 2 Advanced (Magnetic) Theranostic Nanostructures Lab, International Iberian Nanotechnology Laboratory, Av. Mestre José Veiga s/n, 4715-330 Braga, Portugal; 3 Instituto de Parasitología y Biomedicina López Neyra, CSIC, PTS Granada, Avenida del Conocimiento, 17, 18016 Armilla, Granada, Spain; pablo@ipb.csic.es (P.P.);

**Keywords:** antiprotozoal agent, BODIPY derivatives, *Leishmania major*, molecular modeling, *Trypanosoma brucei*

## Abstract

Leishmaniasis and Human African trypanosomiasis pose significant public health threats in resource-limited regions, accentuated by the drawbacks of the current antiprotozoal treatments and the lack of approved vaccines. Considering the demand for novel therapeutic drugs, a series of BODIPY derivatives with several functionalizations at the *meso*, 2 and/or 6 positions of the core were synthesized and characterized. The in vitro activity against *Trypanosoma brucei* and *Leishmania major* parasites was carried out alongside a human healthy cell line (MRC-5) to establish selectivity indices (SIs). Notably, the *meso*-substituted BODIPY, with 1-dimethylaminonaphthalene (**1b**) and anthracene moiety (**1c**), were the most active against *L. major*, displaying IC_50_ = 4.84 and 5.41 μM, with a 16 and 18-fold selectivity over MRC-5 cells, respectively. In contrast, the mono-formylated analogues **2b** and **2c** exhibited the highest toxicity (IC_50_ = 2.84 and 6.17 μM, respectively) and selectivity (SI = 24 and 11, respectively) against *T. brucei*. Further insights on the activity of these compounds were gathered from molecular docking studies. The results suggest that these BODIPYs act as competitive inhibitors targeting the NADPH/NADP^+^ linkage site of the pteridine reductase (PR) enzyme. Additionally, these findings unveil a range of quasi-degenerate binding complexes formed between the PRs and the investigated BODIPY derivatives. These results suggest a potential correlation between the anti-parasitic activity and the presence of multiple configurations that block the same site of the enzyme.

## 1. Introduction

*Leishmania major* and *Trypanosoma brucei* are the pathogenic parasites responsible for two neglected tropical diseases (NTDs): leishmaniasis and Human African trypanosomiasis (HAT, also known as sleeping sickness). Due to the absence of an approved vaccination for these pathogenic diseases and the high toxicity and drug resistance associated with current antileishmanial and antitrypanosomal drugs, ongoing efforts are being dedicated to develop improved molecules to address the limitations of the existing treatments [1]. Notably, in 2021, the US Food and Drug Administration (FDA) approved fexinidazole as the first all-oral treatment for both stages of the *Trypanosoma brucei gambiense* form of sleeping sickness. In 2023, the approval by the European Medicines Agency followed. In any case, new potential treatments are advisable for HAT and also for leishmaniasis.

Recently, our group reported the investigation of *bis*(indolyl)methane (BIM) derivatives substituted with different (hetero)aromatic moieties as antiparasitic agents. We found that the triphenylamine-functionalized compound exhibited an IC_50_ of 3.21 and 3.30 μM against *Trypanosoma brucei* (*T. brucei*) and *Leishmania major* (*L. major*), respectively, and an eight-fold selectivity over a healthy cell line (MRC-5) [2].

Identifying and understanding molecular targets associated with the survival of parasites plays an important role in advancing drug discovery towards antiparasitic agents. Numerous studies have elucidated the potential of target-based drug discovery for parasitic diseases, including HAT and leishmaniasis. These findings have been comprehensively reviewed previously [3,4]. For example, dihydrofolate reductase (DHFR) and pteridine reductase 1 (PTR1) are NADPH-dependent enzymes involved in the reduction of pterins and folates of the trypanosomatid parasites. A greater understanding of the folates pathway in trypanosomatid biology highlighted their crucial role in cellular mechanisms, such as DNA and protein synthesis [5]. Therefore, by disrupting these cellular processes, drugs targeting the folate pathway (also known as antifolates) have the potential to effectively combat trypanosomatid infections. Inhibitors of DHFR, such as trimethoprim and chloroguanide, have been used for the treatment of bacterial infections and malaria, respectively [6,7,8]. However, blockers of the DHRF have shown less efficacy against *Trypanosoma* and *Leishmania* parasites, mainly due to the presence of the PTR1 enzyme [9]. Studies demonstrated that PTR1 is upregulated under DHFR inhibition, thus contributing to the parasites’ resistance to antifolates [10]. Hence, additional PTR1 inhibition has been proposed as an alternative to suppress the trypanosomatidae folate pathway in order to avoid this mechanism of resistance [11,12]. In the past years, several compounds targeting PTR1 have been developed, for example, quinoxaline-based, 2,4-diaminopteridine-based and thiadiazoles-based derivatives [11,13,14,15]. However, additional advancements are required to enhance their selectivity and effectiveness in combating these pathogenic parasites.

The derivatives based on the boron-dipyrromethene (BODIPY) scaffold represent a versatile class of fluorophores widely employed in different scientific fields. The first BODIPY was synthesized by Alfred Treibs and Franz-Heinreich Kreuzer in 1968 [16] and since then, there has been an exponential emergence in the design and synthesis of new compounds. The relevance of these derivatives arises from their facile synthesis and structural versatility. There are numerous functionalization strategies to develop a desirable framework pattern with specific features, including pre-functionalization of the pyrrole/aldehyde and post-functionalization of each position of the core [17,18]. The synthetic flexibility of this scaffold has given rise to a plethora of derivatives with fine-tuned properties, making them versatile tools for diverse applications, such as organic light-emitting diodes (OLEDs), chemosensors, fluorescence probes for bioimaging and photosensitizers in Photodynamic Therapy (PDT) [19,20,21,22,23].

As a matter of fact, BODIPY derivatives are demonstrating a growing success in the field of Photodynamic Therapy (PDT) and Photodynamic Inactivation (PDI) [24,25]. These two therapeutic approaches have become an alternative, especially for cancer treatment and against resistant pathogenic agents, such as bacteria, viruses and fungi. Several approaches have been attempted to fine-tune the BODIPY framework and improve its efficiency in generating reactive oxygen species [26,27,28].

BODIPY-based molecules are also increasingly prominent in the bioimaging field, ascribed to their excellent photophysical properties and great photostability in biological conditions [20]. Interestingly, BODIPY-based fluorescence probes have been developed to investigate the cellular internalization and biodistribution of antiparasitic drugs [29,30,31]. A BODIPY-fluorophore based probe for an anti-Chagas agent was developed and investigated for its potential as an in vivo theranostic probe [30]. Moreover, fluorescent analogues of the leishmanicidal drug miltefosine were successfully developed by integrating a BODIPY moiety on the alkyl chain. These derivatives demonstrated in vitro antiparasitic activity, comparable to that of the original alkylphosphocholine compound [31]. Nevertheless, to the best of our knowledge, the investigation of BODIPY derivatives as the active moiety of antiparasitic agents has not been reported previously.

**Figure 1:**
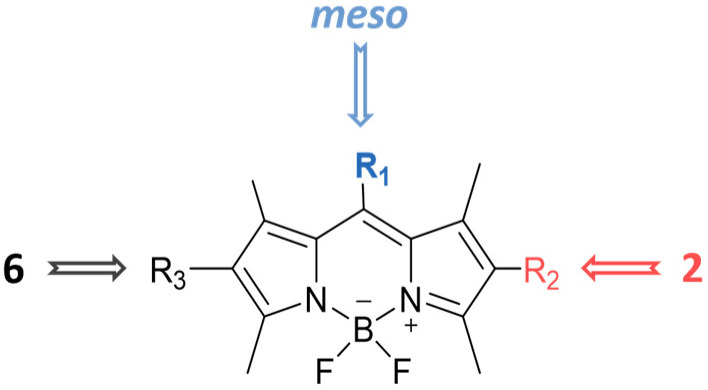
Functionalization of the BODIPY core at *meso* position, 2 position and 6 position with different substituents groups.

In this work, we report on the therapeutic activity of BODIPY derivatives against protozoan parasites. We prepared a series of BODIPY derivatives featuring different functionalizations at the *meso*, 2 and/or 6 positions of the scaffold structure (Cf. Figure 1). The in vitro effectiveness of these compounds was assessed against *T. brucei* and *L. major*, along with the human lung fibroblast cell line (MRC-5), to establish their selectivity indices (SIs). Additional understanding regarding the antitrypanosomal and antileishmanial activity of the BODIPY derivatives was obtained by employing molecular modeling methods to analyze the interactions of the compounds with the pteridine reductase (PR) enzyme.

## 2. Results and Discussion

### 2.1. Synthesis and Characterization of BODIPY Derivatives

**Scheme 1:**
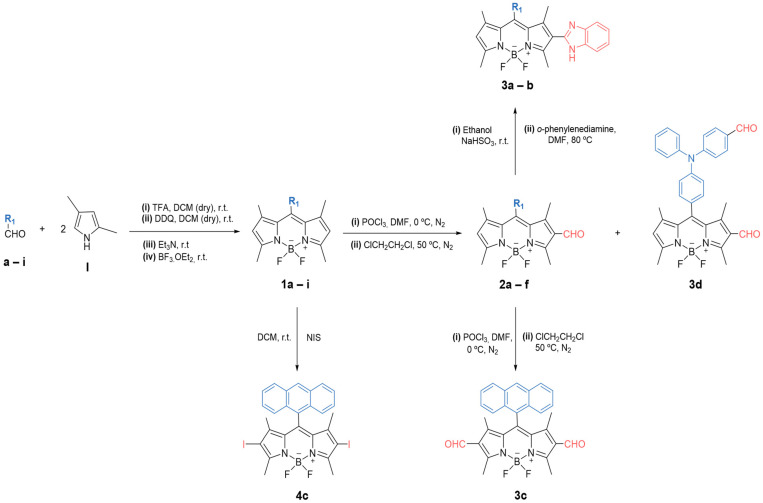
Synthetic route for the BODIPY derivatives functionalized at *meso* position and 2 and/or 6 positions.

A series of BODIPY derivatives were prepared and characterized in order to investigate the effects of the different chemical modifications at positions 2, 6 and *meso* of the core on their biological activity against *T. brucei* and *L. major* parasites. The route for the synthesis of the BODIPY derivatives is represented in Scheme 1, and the functionalization groups at each position of the BODIPY core (R_1_, R_2_ and R_3_), the reaction yields and the photophysical characterization in acetonitrile solution are compiled in Table 1. In this work, the nomenclature employed is as follows: the precursor compounds (R_2_ = R_3_ = H, Cf. Figure 1) of the core are denominated with number **1**; the successor compounds functionalized at R_2_ and/or R_3_ are denominated with numbers from **2** to **4**; and the suffix letters **a** to **i** denote the *meso* substituent (R_1_). The synthesis and characterization of the BODIPY derivatives **1a**, **2a**, **3a**, **1b**, **2b**, **3b**, **1c**, **2c**, **4c**, **1d**, **2d**, **1e** and **2e** have been previously reported by our group [27,28,32,33,34,35,36,37,38,39,40]. The synthesis of the BODIPY derivatives **1f**–**h** has been reported previously elsewhere [41,42,43].

Firstly, to obtain the BODIPY scaffold substituted at *meso* position with several aromatic and heteroaromatic groups (**1a**–**i**), we followed the established Lindsey’s method, as reported by our group previously [27,32,33,34,35,36,44], with yields ranging from 3% to 74%, probably explained by the lower reactivity of the precursor aldehydes, and the difficulty in the purification, by silica gel column chromatography, especially for the thiophene, thiazole and thieno[3,2-*b*]thiophene derivatives **1f**–**i**. The BODIPYs formylated in position 2 and/or 6 (**2a**–**f**) were obtained from the precursors **1a**–**f** via the Vilsmeier-Haack reaction [27,28,32,36,37,38,39] in moderate to excellent yields (47–91%). In the case of the formylation of precursor **1d**, the reaction resulted in two products: the monoformylated compound **2d** (yield 25%), with an aldehyde group on the BODIPY core and the diformylated compound **3d** (yield 26%), with a second aldehyde group on the triphenylamine moiety. The diformylated BODIPY **3c** substituted with two aldehydes groups on the BODIPY core was obtained by a second formylation of its precursor **2c**, with a lower yield (10%). The derivatives functionalized with a benzimidazole at position 2 of the core (**3a** and **3b**) were obtained, in fair to good yields (77% and 31%, respectively), by the condensation between the aldehyde group and *o*-phenylenediamine, followed by an intramolecular cyclisation reaction [32,36]. Finally, derivative **4c** was obtained by the direct halogenation of the precursor **1c** with *N*-iodosuccinimide (NIS), with a yield of 57% [28]. The NMR spectroscopic characterization of compounds **1f**, **1g** and **1h** were in agreement with the previously published data [41,42,43]. The new compounds **3c**, **3d**, **2f** and **1i** were characterized through NMR spectroscopy and high-resolution mass spectrometry. The synthetic methods and characterizations are described in the supporting information.

A comprehensive study focused on the influence of the electron donor/withdrawing substituents at the *meso* and 2 positions of the BODIPY core and solvent polarity on the photophysical properties of the BODIPY derivatives **3a**, **1b**, **2b**, **1c** and **2c** has been recently reported by our research group [27,36]. Overall, the derivatives are characterized by an absorption band, with maxima at 492–512 nm, and emission maxima at 507–524 nm, the typical features for the BODIPY chromophore [19]. Nevertheless, the anthracene-BODIPY substituted with iodine atoms at positions 2 and 6 (compound **4c**) displayed the highest absorption maxima of the series at 540 nm and emission maxima at 559 nm. Comparison of derivatives **1d** and **3d** shows that the introduction of the electron-withdrawing formyl group, at position 2 of the BODIPY core and at the triphenylamine moiety, leads to a red-shift of the maximum emission band (λ_fluo_ = 519 nm vs. λ_fluo_ = 549 nm, respectively), whereas the absorption maximum did not suffer significant changes. Regarding the relative fluorescence quantum yield (*Φ_F_*), in general, the BODIPY derivatives demonstrated low fluorescence intensity, with the exception of compounds **1a**, **2a** and **2f**, which exhibited values of 0.68, 0.84 and 0.30, respectively. Comparison between compounds **1a** and **2a** with their counterpart **3a** shows that the modification with the benzimidazole induces a fluorescence quenching (*Φ_F_* = 0.68 and *Φ_F_* = 0.84 vs. *Φ_F_* = 0.10, respectively). In the case of derivative **2f**, bearing a thiophene moiety at the *meso* position of the BODIPY core and an electron acceptor formyl group at position 2, the compound demonstrated a higher fluorescence emission’s efficiency when compared to its analogue **1f**, non-substituted with the formyl group (*Φ_F_* = 0.30 vs. *Φ_F_* = 0.045, respectively).

### 2.2. Antiparasitic and Cytotoxicity Activity

The in vitro antitrypanosomal and antileishmanial activity of the BODIPY derivatives were evaluated on the bloodstream form (BSF) of *T. brucei* and the promastigote form of *L. major* parasites. The toxicity of the compounds was determined after 72 h of incubation using the alamarBlue assay (for *T. brucei*) and the MTT assay (for *L. major*). The cytotoxicity of the compounds on a healthy human cell line (human lung fibroblast, MRC-5) was also determined after 72 h of incubation using the alamarBlue assay. The data were expressed as the half maximal inhibitory concentration (IC_50_). The selectivity index (SI) of each derivative was calculated by dividing the IC_50_ of MRC-5 cells into the IC_50_ of *T. brucei* or *L. major*. The results are shown in Table 2.

We observed that the BODIPY derivatives **1b**, *meso*-substituted with 1-dimethylaminonaphthalene, and **1c**, *meso*-substituted with anthracene moiety, were the most active of the series against *L. major*, exhibiting IC_50_ values of 4.84 and 5.41 μM, together with a 16 and 18-fold selectivity over the healthy cell line (MRC-5), respectively. In contrast, the cytotoxicity of the derivative **3a**, functionalized at the *meso*-position with a phenyl group and with a benzimidazole moiety at position 2, was higher for the healthy cell line than towards the *L. major* (IC_50_ = 63 μM vs. IC_50_ > 100 μM, respectively), which resulted in a SI lower than one. Interestingly, the compound exhibited an inhibitory effect on *T. brucei* with an IC_50_ value of 5.5 μM and an SI value of 11.38. The formylated derivatives **2b** and **2c** also exhibited good inhibitory activity against *T. brucei*, particularly compound **2b** (substituted with 1-dimethylaminonaphthalene at *meso* position). This compound displayed the best IC_50_ 2.84 μM and selectivity index of the series and a 24-fold selectivity over MRC-5, which suggests an interesting therapeutic potential of **2b** against *T. brucei* parasites. Moreover, inhibitory concentration values of 3.6 μM against *L. major* and 2.7 μM against *T. brucei* have been reported for methotrexate, a reference antifolate drug [12,45]. These values fall within the range of the toxicity exhibited by the most active BODIPY derivatives **1b**, **2c** and **3c** towards *L. major* and compound **2b** towards *T. brucei*.

The functionalization of the BODIPY core with a formyl group seems to influence, in certain cases, their antiparasitic activity. The unsubstituted compounds **1b** and **1c** were more active against *L. major*, whilst the mono-formyl analogues **2b** and **2c** were more cytotoxic against *T. brucei* parasites. Additionally, in *T. brucei*, the BODIPYs functionalized at R_3_ with formyl were overall more toxic than the unsubstituted analogues, hence with higher selectivity indices (C.f. Figure S1). For instance, compound **2b** has an IC_50_ of 2.84 μM (SI = 24.34), whilst its precursor **1b** has an IC_50_ = 18.24 μM (SI = 4.21); compound **2c** shows an IC_50_ = 6.17 μM (SI = 10.84) and its unsubstituted analogue **1c** an IC_50_ = 77.11 μM (SI = 1.07); compound **2f** displays an IC_50_ of 12.37 μM (SI = 6.54), whilst its counterpart **1f** shows an IC_50_ = 36.93 μM (SI = 1.57). However, this tendency seems not to be so relevant for antileishmanial activity (C.f. Figure S2).

### 2.3. Molecular Docking Studies

In order to rationalize the antileishmanial and antitrypanosomal activity data presented in Table 2 for the 20 BODIPY derivatives, their interaction with pteridine reductase native to *L. major* (PRLm) and *T. brucei* (PRTb) was studied using a molecular docking protocol.

The selection of Pteridine reductase as the probable target for these BODIPY derivatives was predicated on its structural resemblance to known antileishmanial and antitrypanosomal drugs. A quantitative assessment of the structural similarities was conducted through the analysis of Morgan fingerprint bit differences (Cf. Table S1).

**Figure 2:**
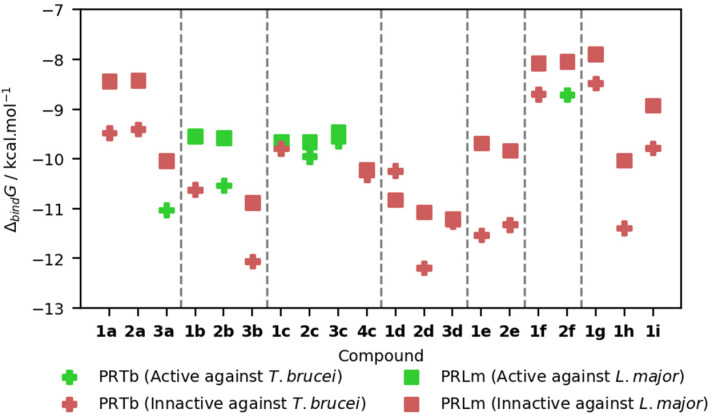
Binding Gibbs energies (${∆}_{bind}G$, in kcal/mol) for the best binding mode of each enzyme-BODIPY derivative. Compounds **1b**, **1c**, **2b**, **2c**, and **3c** present antileishmanial activity, whereas compounds **2b**, **2c**, **2f**, **3a**, and **3c** present antitrypanosomal activity.

Figure 2 summarizes the main results from the molecular docking studies. In general, all BODIPY derivatives present good binding energies for the most stable binding mode, and ${∆}_{bind}G$ is generally lower for PRTb than for PRLm, with the noticeable exception of **1d**. As it will be detailed later, the attachment of the BODYPY derivatives is mostly established via hydrophobic interactions between the *sp*^2^ carbon atoms of the BODIPY derivative and the carbon atoms on the side chains of a handful of amino acid residues (Cf. Figures S3–S11). The only significant exceptions noted involved the possible hydrogen bonding between PRLm and compounds **1b**, **2b** and **3b**, involving the H atoms of the amine group and the backbone N of Ala15, the heterocyclic N of Hys241 and the O atom of Ser111, respectively. Compound **3b** also forms a hydrogen bond with the amino moiety at the side chain of Arg14 of PRTb.

Although the results depicted in Figure 2 do not allow for a clear distinction between active and inactive compounds, the docking studies found additional low-energy binding modes, some of which translate into a significantly different geometry of the enzyme-ligand complex.

In most cases, the set of 30 binding modes with binding energies lower than 5 kcal·mol^−1^, relative to the most stable mode, could be classified into 2 or 3 families of nearly degenerate modes. The importance of these observations was first accessed by estimating the probability $p_{i}$ of finding each enzyme-BODIPY derivative complex at a given binding mode *i*:

(1) $$p_{i}=\frac{g_{i}e^{\frac{-{∆}_{bind}G_{i}}{RT}}}{\sum_{j}^{N_{modes}}g_{j}e^{\frac{-{∆}_{bind}G_{j}}{RT}}}$$

where T = 298.15 K, R is the gas constant (in kcal/mol) and $g_{i}$ is the degeneracy of each binding mode. Because the modes are nearly, but not completely, degenerate, all calculations were carried out using $g_{i}$ = 1. Assuming an exhaustive search of the conformational space and that the collected modes are representative of the lowest energy states of the system, these probabilities reflect the population of each binding mode [46]. The complete results from these calculations in the form of the population of each binding mode for each enzyme-BODIPY derivative are given in Tables S2 and S3 in the Supporting Information.

In general, the results show that there is a high probability that the most stable binding mode is not representative of the thermally available complexes. Indeed, the higher $p_{i}$ values were found for the lowest energy binding mode of PRLm-**3d** (48%) and PRTb-**3b** (44%). These, however, are exceptions, with the second higher $p_{i}$ being observed for the complexes of **2d** with PRLm and PRTb, which corresponded to populations of only 26% and 39%, respectively. It should be noted that the ligand in each of these highly stable complexes is not active against *L. major* nor *T. brucei*. On the other hand, the results show that most complexes with antileishmanial and antitrypanosomal compounds present a population of the lowest energy binding mode smaller than 20%, with the exception of PRTb-**2b**, for which the lowest energy binding mode represents 36% of the population.

Thus far, the results from the molecular docking studies strongly suggest that most BODIPY derivatives have some freedom to move within the binding cavity of the tested enzymes, making the lowest energy binding mode less representative than what ought to be expected. As a consequence, the affinity of each compound for the enzyme cannot be rationalized solely based on the geometry of this binding mode, prompting us to explore more sophisticated ways of understanding the formation of these complexes; why are only a handful of BODIPY derivatives biologically active, and why some are specific to a given parasite? For this purpose, the geometry of each binding mode was analyzed in order to find the 10 closest contacts Nc between the enzyme and the ligand; for each contact, we recorded the amino acid involved, the atoms of the ligand and amino acid involved, as well as the distance between the atoms at close contact. For each ligand, the contact to each amino acid in the enzyme was weighted by the $p_{i}$ of the binding mode. Thus, for each amino acid A and ligand L, the overall affinity between a given amino acid, and a ligand is the number of closest contacts between A and L, weighted by $p_{i}$:

(2) $$Aff\left(A,L\right)=\sum_{j}^{N_{modes}}100.0\times p_{i}card\left(A\in C_{i}\right)$$

where $C_{i}$ is the set of amino acids containing atoms in close contact with the ligand in the binding mode i (limited to the Nc closest contacts) and $card\left(A\in C_{i}\right)$—cardinality of $A\in C_{i}$—denotes the count of amino acid *A* in the set of closest contacts.

The results from using this unitless affinity metric were quite interesting, as they highlighted the affinity of the BODIPY derivatives to certain amino acids in and around the binding pocket. Considering all the 20 BODIPY derivatives, only 57 of the 301 amino acids in PRLm’s chain A have some affinity for these ligands, representing about 19% of the enzyme’s primary structure. In the case of PRTb, affinity toward the BODIPY derivatives was found for 61 of the 267 amino acids in chain A, which covers about 23% of the primary structure. Nevertheless, the values of $Aff\left(A,L\right)$ vary by more than three orders of magnitude, from less than 0.1 to about 325 (measured for Aff(Val206, **2b**) in PRTb).

**Figure 3:**
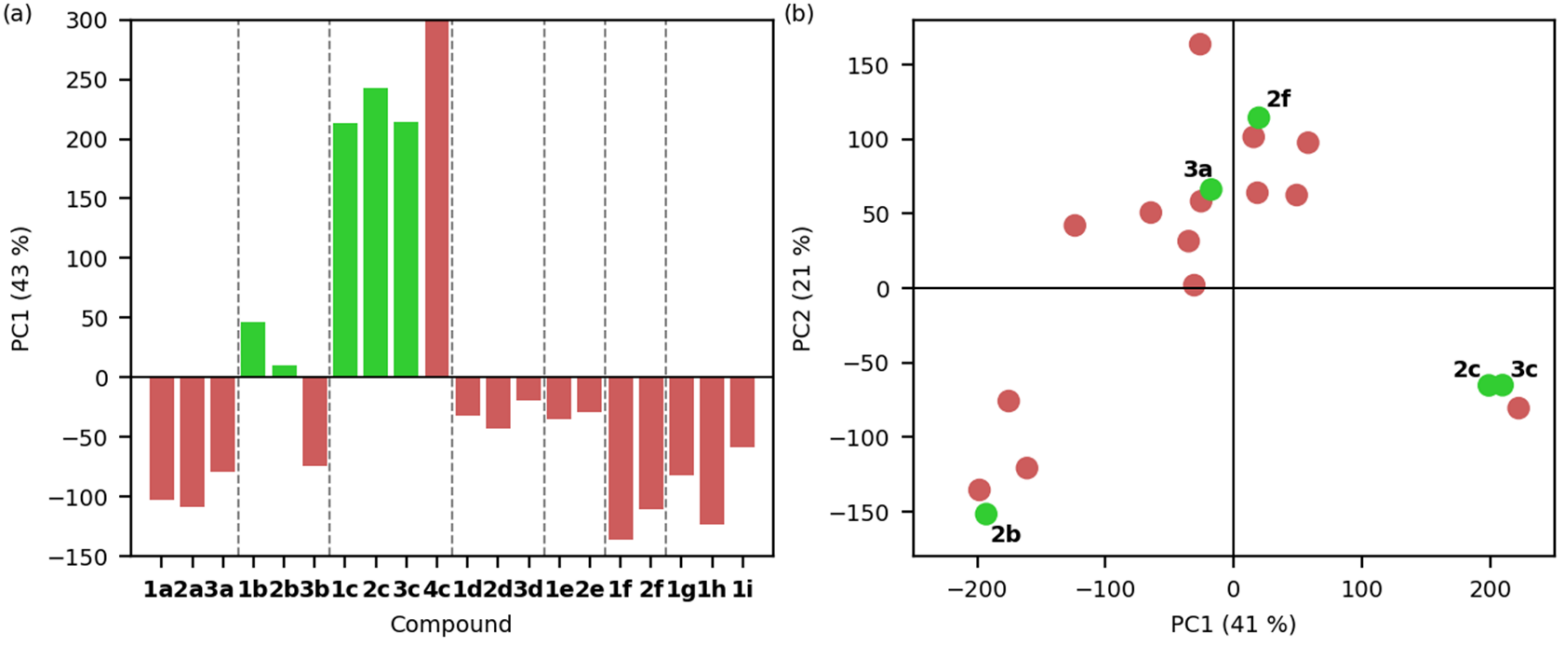
Principal Component Analysis (PCA) scores of the affinity data for PRLm (**a**) and PRTb (**b**) from the molecular docking studies of the 20 BODIPY derivatives binding to the active site at chain A of each enzyme. The variance explained by each PC is given in parenthesis, as a percentage of the total variance in each data set. Compounds with antileishmanial (**a**) or antitrypanosomal (**b**) activity are depicted in green and are further labeled in (**b**), and inactive ones are depicted in red.

Although some patterns in $Aff\left(A,L\right)$ were apparent by a simple visual depiction of the data, a Principal Component Analysis (PCA) of the affinity data provided a much-needed simplification of the data. In the case of PRLm, the first Principal Component (PC), representing 43% of the variance in the data, clearly classifies the active from the inactive compounds, as shown in Figure 3a, with the exception of **4c**. This erroneous classification of **4c** may be due to its low solubility in water, which may have prevented it from reaching PRTb during the biological assays.

On the other hand, PCA was unable to resolve the compounds for which antitrypanosomal activity was observed from those lacking it, as shown in Figure 3b, which depicts the distribution of the data along PC1 and PC2, covering 62% of the variance in the data.

This intriguing result prompted more details of the loadings associated with PC1 for the data concerning PRLm. Table 3 shows the seven most prominent loadings along the positive (pointing toward antileishmanial activity) and negative (pointing toward lack of antileishmanial activity) directions of PC1. These results highlight that affinity toward Ser40 and Ala15 of PRLm are desirable traits when searching for antileishmanial activity. On the other hand, significant affinity toward Phe113, as well as affinity toward a number of hydrophobic amino acids in the 224 to 230 positions, appear to hinder the antileishmanial activity of the BODIPY derivatives.

**Figure 4:**
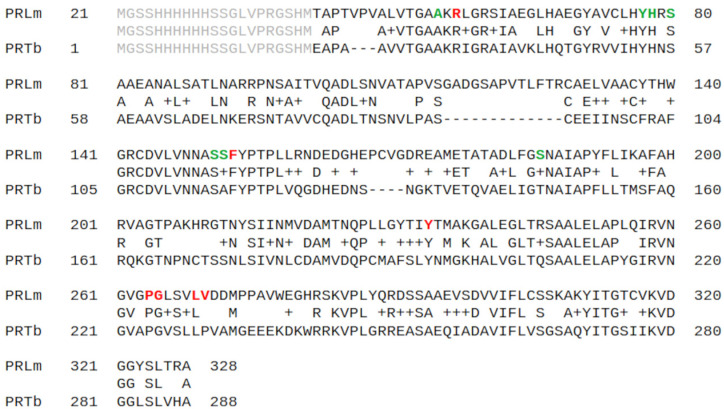
Alignment of the primary structure of PRLm (PDB code 2QHX) and PRTb (PDB code 4CM7). In the structure of PRLm, affinity toward the highlighted amino acids determines antileishmanial activity (green), or lack thereof (red), according to the data shown in Table 3. The initial sequence of 21 amino acids is due to the technique used to express these proteins during the sequencing procedure. The initial 21 amino acids, marked in grey, derive from the cloning strategy used in the sequencing protocol.

**Figure 5:**
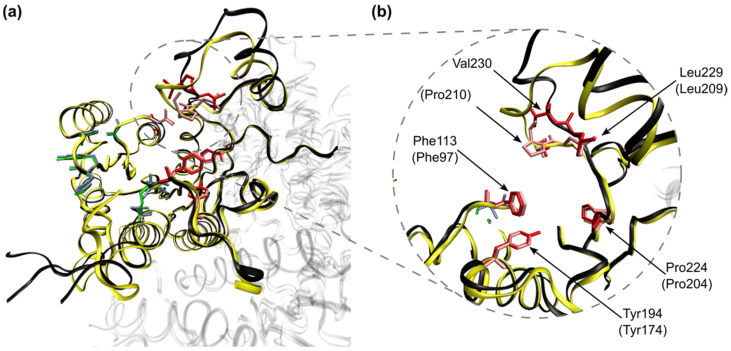
Aligned structures of PRLm (black) and PRTb (yellow) in ribbons representation. Amino acid residues targeted for antileishmanial activity (positive loading value in Table 3) are shown in the structure of PRLm in green, and their homologues in PRTb are highlighted in light blue. Amino acid residues associated with a lack of antileishmanial activity (negative loading value in Table 3) are highlighted in red (PRLm), whereas their homologues in PRTb are depicted in pink. (**a**) The overall structure of chain A (chains B, C and D of both enzymes are depicted using high transparency). (**b**) Detailed view of the pocket surrounding Val230 in PRLm, with the structural homologues in PRTb labeled inside parenthesis.

With the results from PRLm in mind, we sought to find in the primary structure of PRTb, the homologous amino acids to those listed in Table 3 using the aligned primary structures of both compounds, as shown in Figure 4. As shown in Figure 4, almost all amino acids listed in Table 3 are present in both PRLm and PRTb. Despite that, a few of these amino acids are replaced by similar ones: Ser112 is replaced in PRTb by alanine (Ala96) and Ser146 is replaced by threonine (Thr126), only one amino acid mentioned in Table 3 (Val230) is replaced by a substantially different one (Pro210). Moreover, the two enzymes align quite well, with the amino acids highlighted by PCA in PRLm overlaying their homologues in PRTb, as shown in Figure 5.

Looking at the aligned structures shown in Figure 5, it is clear that the antileishmanial activity is linked to the compound’s ability to bind to the more peripheral side of the binding cavity, whereas affinity towards the internal face of this cavity will likely lead to a lack of antileishmanial activity. Furthermore, Figure 5a shows that the amino acids more likely to bind to an antileishmanial BODIPY derivative occupy the same position and orientation in both PRLm and PRTb. In both cases, these amino acids form the pocket where the adenine moiety of NADPH binds to these enzymes, allowing one to postulate that these BODIPY derivatives compete with NADPH for the binding cavity.

Although most amino acids considered as undesirable binding spots (Figure 5a, depicted in red) also line up well between PRLm and PRTb (depicted in pink), it is noticeable that replacing Val230 with Pro210 leads to a local discrepancy in the three-dimensional structure of these enzymes, which is highlighted in Figure 5b. This is evident by the lack of overlap between PRLm and PRTb in the turn domain following Pro210, as well as some misalignment of the *α*-helix that follows. Moreover, Pro210 is oriented towards Phe113, forming a narrow space that may trap planar molecules or groups, such as some of the *meso* substituents of the BODIPY derivatives described in this work. In a similar manner, the narrowing of the space close to Phe113 may drive some bulkier compounds to seek the binding spots in the peripheral face of the binding cavity, hindering the enzyme’s ability to bind to NADPH.

**Figure 6:**
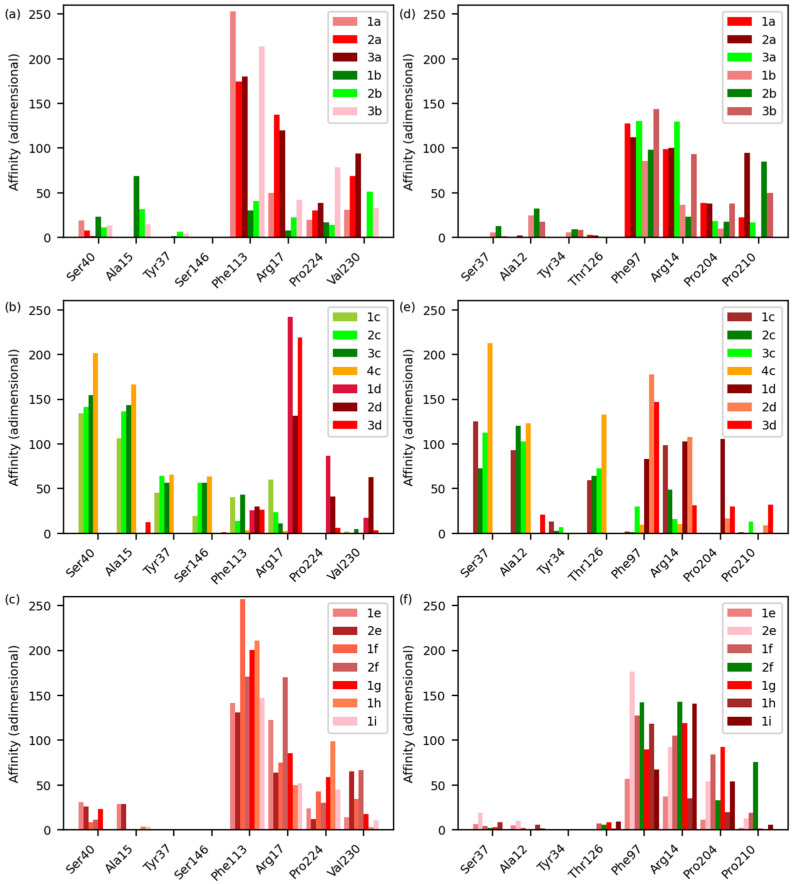
Dimensional affinities (Cf. Equation (2)) towards key amino acid residues in PRLm impacting the antileishmanial activity of BODIPY derivatives: compounds of the (**a**) **a** and **b** series; (**b**) **c** and **d** series; (**c**) **e** and **f** series, as well as **1g**–**1i**. Likewise, for the structural homologues in PRTb (**d**) **a** and **b** series; (**e**) **c** and **d** series; (**f**) **e** and **f** series, as well as **1g**–**1i**. Compounds showing antileishmanial activity are depicted in various shades of green, whereas those lacking activity are shown in various shades of red (**a**–**c**). A similar color coding is applied for antitrypanosomal activity (**d**–**f**).

This rationale leads to the hypothesis that the inhibition of PRTb is driven by the affinity to the amino acids that are homologues of the ones driving the inhibition of PRLm. In order to test this hypothesis, the affinities of each BODIPY derivative toward the amino acids listed in Table 3, or their homologues in PRTb, are depicted in Figure 6.

The results depicted in Figure 6 provide some validation of our hypothesis. Indeed, all antileishmanial compounds show considerable affinity toward Ala15, whereas none of the compounds inactive against *L. major* show affinity toward this amino acid residue, as depicted in Figure 6a–c. The same observation can be noted for Tyr37 and Ser146. On the other hand, BODIPY derivatives lacking antileishmanial activity have an increased affinity toward Phe113, Arg117 and (to a lesser extent) toward Pro224 and Val230. It should be noted that some of the BODIPY derivatives with the smallest IC_50_ against *L. major* also feature some affinity toward Ser40. It is also noteworthy that the preferential orientation of the active BODIPY derivatives toward these amino acid residues involves pointing the group in the *meso* position towards said amino acid (regardless of the amino acid being associated with antileishmanial activity, or lack thereof).

Thus far, the results from the ensemble of configurations collected in the docking studies show that the affinity of the *meso* group toward specific amino acid residues is a good indicator of the possible antileishmanial activity, with the already justified exception of compounds **4c**. In order to fully validate our hypothesis, some rules concerning antitrypanosomal activity should also be inferred from the data concerning the docking studies carried out using PRTb. Indeed, some commonalities are found between Figure 6a–c and their PRTb counterparts (Figure 6d, Figure 6e, and Figure 6f, respectively), such as the overall high affinity of non-antitrypanosomal BODIPY derivatives toward Phe97 (homologue of Phe113 in PRLm) or the relatively high affinity of antitrypanosomal compounds **2c** and **3c** towards Ala12 (homologue of Ala15 in PRLM, Figure 6e). On the other hand, a plain application of the rules devised for PRLm may be misleading. For example, antitrypanosomal compounds **3a** and **2b** show high affinity toward Phe97 and Arg14 and low or no noticeable affinity towards amino acids that would be associated with the inactivation of PRTb such as Ala12 (homologue of Ala15 in PRLm). Interestingly, these two compounds show some affinity toward Pro210, suggesting the possibility that these compounds can stay “pinched” between Phe97 and Pro210, effectively preventing the nicotinamide moiety of NADPH to bind.

The BODIPY derivatives from the **c** and **d** series show similar affinity profiles in both PRLm (C.f. Figure 6b) and PRTb (C.f. Figure 6e), suggesting that they may share the same inactivation mechanism for both enzymes. However, all antileishmanial compounds from the **c** series show a decreased affinity toward Tyr34. Moreover, compound **1c** also shows increased affinity toward Arg14, which suggests that this compound is more labile inside PRTb’s cavity, which may lead to its displacement by NADPH (or water, or another cofactor).

Finally, compound **2f** does not present antileishmanial activity, showing an affinity profile toward PRLm similar to other non-antileishmanial BODIPY derivatives (C.f. Figure 6c). In the presence of PRTb, this compound shows some significant affinity toward Pro210, suggesting again a stabilization of its binding configuration at the location where the nicotinamide moiety of NADPH binds to PRTb.

Overall, the results from the docking studies show that the BODIPY derivatives considered in this work have two inactivation mechanisms towards pteridine reductase: they may bind to the Ser, Ala and Tyr residues in the most peripheral area of pteridine reductase’s active cavity, preventing the adenine moiety of NADPH from binding, or they may attach closer to Phe113, Pro204 and Val230 (or their homologues in PRTb) and interfere with the binding of NADPH’s nicotinamide moiety. This latter mechanism is much less effective in PRLm and usually leads to an incomplete inactivation of PRLm, allowing the parasite to survive. On the other hand, the structural differences between PRLm and PRTb allow the nicotinamide approach to be an effective way to inhibit PRTb, via the formation of stable enzyme-BODIPY derivatives in which the latter are kept stable between Phe97 and Pro204. Most importantly, the results from this study showed a multitude of quasi-degenerate binding complexes between either enzyme and the BODIPY derivatives under study, suggesting that antileishmanial and antitrypanosomal activities may be linked to the existence of a number of quasi-degenerated configurations blocking the same moiety of the target enzyme. Indeed, the results from the docking studies strongly suggest that this effect, closely related to the entropy of the binding complexes, surpasses the mere evaluation of the binding energy between the enzyme and its ligands when it comes to predicting anti-parasitic activity.

## 3. Conclusions

In conclusion, we prepared a series of BODIPY derivatives functionalized at the *meso*, 2 and/or 6 positions of the core and evaluated their antileishmanial (*L. major* parasites) and antitrypanosomal (*T. brucei* parasites) activity. The studies revealed promising anti-parasitic activities, with compounds **1b** and **1c**, *meso*-substituted with 1-dimethylaminonaphthalene and anthracene moieties, standing out as the most potent of the series against *L. major*. These derivatives exhibited IC_50_ values of 4.84 and 5.41 μM, along with selectivity indices of 16 and 18-fold over healthy cells, respectively. Concerning the antitrypanosomal activity, the formylated derivatives **2b** and **2c** displayed significant efficacy against *T. brucei*, with compound **2b** demonstrating the highest inhibitory effect and selectivity (IC_50_ = 2.84 μM and SI = 24.34) within the series.

Additionally, the docking studies conducted on the BODIPY derivatives unveil two distinct inactivation mechanisms for pteridine reductase: (a) these derivatives exhibit an ability to bind to specific residues, including Ser, Ala and Tyr, preventing the adenine moiety of NADPH from binding; (b) blocking NADPH’s nicotinamide moiety from binding by interference with Phe113, Pro204 and Val230 in PRLm (or their homologues in PRTb). On the one hand, the second mechanism seems to be less effective against PRLm, leading to an incomplete inactivation of the pteridine reductase in *L. major*. On the other hand, structural disparities between PRLm and PRTb underscore the effectiveness of the nicotinamide approach in inhibiting the enzyme in *T. brucei*. Moreover, this study highlights the significance of quasi-degenerate binding complexes, indicating a potential correlation between antileishmanial and antitrypanosomal activities and various configurations hindering the target enzyme’s moiety. This emphasizes the importance of considering these multiple binding configurations beyond traditional binding energy assessments in predicting anti-parasitic activity.

## 4. Experimental Section

### 4.1. General

NMR spectra were obtained on a Bruker Avance III 400 at an operating frequency of 400 MHz for ^1^H and 100.6 MHz for ^13^C, at 25 °C using the solvent peak as an internal reference. All chemical shifts are given in ppm using *δ*_H_ Me_4_Si = 0 ppm as a reference. Peak assignments were supported by spin decoupling-double resonance and bidimensional heteronuclear techniques. High-resolution mass spectrometry analysis was performed at the “C.A.C.T.I.—Unidad de Espectrometria de Masas” at the University of Vigo, Spain. All reagents were purchased from Sigma–Aldrich, Acros and Fluka and used as received. Thin-layer chromatography (TLC) was carried out on 0.25 mm thick precoated silica plates (Merck Fertigplatten Kieselgel 60F_254_), and spots were visualized under ultraviolet (UV) light. Mps were determined on a Gallenkamp apparatus. UV-visible absorption spectra (200–700 nm) were obtained using a Shimadzu UV/2501PC spectrophotometer. Fluorescence spectra were collected using a FluoroMax-4 spectrofluorometer. Fluorescence quantum yields were measured using Rhodamine 6G solution in ethanol as standard (*Φ_F_* = 0.95) [47]. The synthesis and characterization of the BODIPY derivatives **1**–**3a**, **1**–**3b**, **1c**, **2c**, **4c**, **1d**, **2d**, **1e** and **2e** has been previously reported by our group [27,28,32,33,34,35,36,37,38,39,40]. The synthesis of the BODIPY derivatives **1f**–**h** has been reported previously elsewhere [41,42,43]. The synthesis and characterization of BODIPY derivatives **1f**–**i**, **2f**, **3c** and **3d** are given in the Supporting Information.

### 4.2. Biological Activity

#### 4.2.1. Parasite and Cell Culturing

*T. brucei* (Lister 427, antigenic type MiTat 1.2, clone 221a, bloodstream forms, “single marker” S427 (S16)) [48] were cultured at 37 °C, 5% CO_2_ in HMI-9 medium supplemented with 10% heat-inactivated fetal bovine serum (hiFBS, Invitrogen, Carlsbad, CA, USA), as previously described [49].

*L. major* (MHOM/IL/80/Friedlin) promastigotes were cultured at 28 °C, 5% CO_2_ in modified RPMI-1640 medium (Invitrogen, Carlsbad, CA, USA) supplemented with 10% hiFBS [49]. Parasites were maintained in culture in their experimental growth phase (below 2 million parasites per mL for *T. brucei* and 10 million parasites per mL for *L. major*).

MRC-5 cell line (human lung fibroblast) was grown in a monolayer at 37 °C, 5% CO_2_ in DMEM medium (1 g/L glucose) supplemented with 10% hiFBS, 100 U/mL penicillin, 100 mg/mL streptomycin and 2 mM L-glutamine [50].

#### 4.2.2. Antiparasitic Activity

The antitrypanosomal activity of the BODIPY derivatives was determined by the alamarBlue^®^ assay (ThermoFisher Scientific, Waltham, MA, USA) [50,51]. The stock solutions of the compounds were prepared in DMSO and the final DMSO percentage in each well was adjusted to be less than 1%. A total of 2 × 10^4^ parasites per mL were incubated at 37 °C, 5% CO_2_ in 96-well plates (50 μL/well) alone or in the presence of an increasing concentration of compounds for 72 h. A total of 20 μL of resazurin solution (110 ng/mL) was then added to each well, and the parasites were incubated for 4 h at 37 °C. Finally, cells were lysed with 50 μL per well of SDS 3%. The plate was incubated at 37 °C for an extra hour and then, fluorescence intensity was measured with an Infinite F200 plate reader (Tecan Austria, GmbH, Grödig, Austria), exciting at 550 nm and recording the emission at 590 nm. The results are expressed as the concentration of a compound that reduces cell growth by 50% versus untreated control cells (IC_50_). Data are presented as the average of at least three independent measurements, all conducted in triplicate conditions.

The antileishmanial activity of the BODIPY derivatives was assessed using an MTT-based assay (Sigma-Aldrich, St. Louis, MO, USA) [52]. The stock solutions of the compounds were prepared in DMSO and the final DMSO percentage in each well was adjusted to be less than 1%. A total of 4 × 10^6^ parasites per mL were incubated at 28 °C in 96-well plates (50 μL/well) alone or in the presence of an increasing concentration of compounds DMSO for 72 h. A total of 10 μL of MTT (5 mg/mL) were added to each well and parasites were incubated for 4 h at 28 °C. Finally, cells were lysed with 50 μL/well of 20% SDS. The plate was incubated at 37 °C for an extra hour and then, absorbance was measured at the Infinite F200 plate reader (TECAN Austria, GmbH, Grödig, Austria) at a wavelength of 540 nm. The IC_50_ was calculated as described above. Data are presented as the average of at least three independent measurements, all conducted in triplicate conditions.

#### 4.2.3. Cytoxicity

Cytotoxicity was measured in MRC-5 through alamarBlue^®^ assay (ThermoFisher Scientific) [50,51]. The stock solutions of the compounds were prepared in DMSO and the final DMSO percentage in each well was adjusted to be less than 1%. A total of 5 × 10^3^ cells per mL were seeded in 96-well plates (100 μL/well) with 24 h of incubation time before compound addition at 37 °C, 5% CO_2_. Then, the compounds were added at increasing concentrations (from 0 to 100 μM) and the plates were incubated for 72 h. A total of 20 μL of resazurin solution (110 ng/mL) was added to each well and cells were incubated for 4 h. Then, cells were lysed with 50 μL/well of 3% SDS. The plate was incubated at 37 °C for an extra hour and then, the fluorescence intensity was measured at the Infinite F200 plate reader (TECAN Austria, GmbH), exciting at 550 nm and recording the emission at 590 nm. The results are expressed as the concentration of a compound that reduces cell growth by 50% versus untreated control cells (IC_50_). Data are presented as the average of at least three independent measurements, all conducted in triplicate conditions.

#### 4.2.4. Molecular Docking

The structures of the BODIPY derivatives were optimized using Density Functional Theory (DFT) calculations using the B3LYP functional [53] in conjunction with Alrichs’ Def2-TZVP basis set [54] for all atoms, except iodine, which was described using the relativistic pseudopotential by Peterson et al. [55]. The equilibrium geometries of these compounds were converted to the pdbqt format to be used in the molecular docking studies, which were updated with the CHELPG charges [56,57] derived from the molecular electrostatic potential obtained from the DFT calculations. All DFT calculations were carried out using the Orca software package, version 5.0.3 [58].

The structures of the native form of pteridine reductase from *Leishmania major* (PRLm) and *Trypanosoma brucei* (PRTb) were obtained from the Protein Data Bank (PDB) [59], with codes 2QHX and 4CM7, respectively. These structures were filtered to remove water, ions, NADPH and any substrate or inhibitor declared in the PDB records. Alignment of the primary structure of the two enzymes was performed using the BLAST software, version 2.15.0 [60,61] linked from inside PDB.

The docking studies were prepared using the MGLTools software suite, version 1.5.7 [62] and the binding mode search and ranking were carried out using AutoDock Vina, version 1.2.5 [63,64]. Both enzymes exist as a tetramer, and the conformational search was restricted to the binding region of chain A, using a grid spacing of 1 Å, an exhaustiveness setting of 24, and collecting the 30 lowest energy binding modes within 5 kcal.mol^−1^ of the lowest energy mode. The specific details concerning the grid box used in each enzyme are given in the Supporting Information. The subsequent treatment of the results from the docking studies was carried out using in-house developed Python scripts, whose source code is transcribed in the Supporting Information.

## Figures and Tables

**Table 1:** Synthesis and photophysical data for the BODIPY derivatives. The photophysical studies were conducted in acetonitrile solutions with concentrations of the compounds ranging from 10^−5^ to 10^−6^ M (absorption, λ_abs_, and fluorescence emission maxima, λ_fluo_, and fluorescence quantum yields, *Φ_F_*).

Compound	R_1_	R_2_	R_3_	Yield (%)	λ_abs_ (nm)	λ_fluo_ (nm)	*Φ*_F_
**1a**	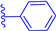	H	H	21	497	513	0.68
**2a**		H	CHO	91	492	508	0.84
**3a**		H	Benzimidazole	77	507	517	0.10
**1b**	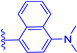	H	H	22	502	515	0.03
**2b**		H	CHO	59	497	509	0.01
**3b**		H	Benzimidazole	31	512	514	0.03
**1c**	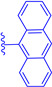	H	H	74	502	515	0.03
**2c**		H	CHO	47	497	509	0.01
**3c**		CHO	CHO	10	500	524	0.009
**4c**		I	I	57	540	559	0.003
**1d**	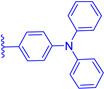	H	H	20	497	519	0.005
**2d**		H	CHO	25	491	515	0.01
**3d**	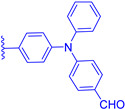	H	CHO	26	493	546	0.011
**1e**	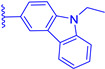	H	H	17	496	507	0.019
**2e**		H	CHO	57	492	506	0.024
**1f**	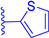	H	H	8	508	519	0.045
**2f**		H	CHO	49	502	517	0.30
**1g**	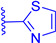	H	H	7	512	524	0.009
**1h**	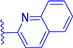	H	H	37	500	512	0.026
**1i**	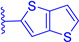	H	H	3	511	522	0.036

**Table 2:** Antiparasitic activity against *T. brucei* BSF and *L. major* promastigotes and cytotoxicity in MRC-5 represented as IC_50_ values (μM) with the error reported as *σ*. The selectivity index was calculated related to a healthy cell line (MRC-5). The best selectivity values (SI > 10) are highlighted in bold.

Compounds	IC_50_ μM			Selectivity Index (SI)	
	*T. brucei*	*L. major*	MRC 5	*T. brucei*	*L. major*
**1a**	38.20 ± 7.29	19.65 ± 3.94	67.16 ± 4.39	1.76	3.42
**2a**	74.68 ± 6.93	>100	91.33 ± 0.66	1.22	<0.91
**3a**	5.5 ± 0.21	>100	62.61 ± 3.27	**11.38**	<0.63
**1b**	18.24 ± 3.63	4.84 ± 1.56	76.84 ± 5.33	4.21	**15.93**
**2b**	2.84 ± 0.04	15.75 ± 1.31	69.12 ± 4.90	**24.34**	4.39
**3b**	27.87 ± 5.42	84.39 ± 0.32	>100	>3.59	>1.18
**1c**	77.11 ± 2.98	5.41 ± 0.43	82.14 ± 0.03	1.07	**17.90**
**2c**	6.17 ± 2.35	10.33 ± 1.59	66.89 ± 7.65	**10.84**	6.47
**3c**	14.15 ± 0.31	3.13 ± 0.13	21.65 ± 3.46	1.53	6.91
**4c**	28.05 ± 1.77	>50	48.22 ± 1.13	1.72	<0.96
**1d**	48.84 ± 3.29	>100	91.29 ± 6.31	1.87	<0.91
**2d**	22.74 ± 1.88	>100	81.06 ± 3.90	3.56	<0.81
**3d**	38.26 ± 0.93	>100	87.13 ± 3.28	2.28	<0.87
**1e**	18.65 ± 2.67	>100	88.89 ± 3.82	4.77	<0.89
**2e**	15.23 ± 0.86	20.71 ± 1.79	89.65 ± 9.08	5.89	4.33
**1f**	36.93 ± 7.43	64.55 ± 8.43	57.96 ± 4.76	1.57	0.90
**2f**	12.37 ± 4.02	19.72 ± 3.21	80.89 ± 1.06	6.54	4.10
**1g**	55.67 ± 1.65	>100	82.82 ± 1.20	1.49	<0.83
**1h**	77.36 ± 3.46	>100	95.58 ± 3.45	1.24	<0.96
**1i**	61.47 ± 10.94	92.21 ± 10.02	96.82 ± 4.50	1.58	1.05

SI = (IC_50_ MRC-5/IC_50_ parasite).

**Table 3:** Most prominent PCA loadings associated with PC1 of the affinity data for PRLm and the set of 20 BODIPY derivatives.

Positive Loadings		Negative Loadings	
Amino Acid	Value	Amino Acid	Value
Ser40	+0.43	Phe113	−0.49
Ala15	+0.40	Arg17	−0.23
Ser111	+0.23	Tyr194	−0.23
His38	+0.23	Gly225	−0.15
Tyr37	+0.17	Pro224	−0.14
Ser112	+0.16	Val230	−0.11
Ser146	+0.14	Leu229	−0.10

## Data Availability

Data are contained within the article and Supplementary Materials.

## Supplementary Materials

The following supporting information can be downloaded at: https://www.mdpi.com/article/10.3390/molecules29092072/s1. Synthesis and characterization of *meso*-substituted BODIPY derivatives **1f**–**i**; Synthesis and characterization of formylated BODIPY derivatives **2f**, **3c** and **3d**; Figure S1. Analysis of the chemical structure—antitrypanosomal activity relationship according to the selectivity index (SI) values of the BODIPY derivatives; Figure S2. Analysis of the chemical structure—antileishmanial activity relationship according to the selectivity index values of the BODIPY derivatives; Table S1. Inter-compound distances based on difference counts of Morgan fingerprints of radius 2, normalized so that the maximum distance between the BODYPI derivatives reported in this work was 1.0; Figure S3. Images of the most stable complexes of **1a** (a), **1b** (b), **1c** (c), **1d** (d), **1e** (e), and **1f** (f) with PRLm, as found in the molecular docking studies; Figure S4. Images of the most stable complexes of **1g** (a), **1i** (b), **1h** (c), **2a** (d), **2b** (e), and **2c** (f) with PRLm, as found in the molecular docking studies; Figure S5. Images of the most stable complexes of **2d** (a), **2e** (b), **2f** (c), **3a** (d), **3b** (e), and **3c** (f) with PRLm, as found in the molecular docking studies; Figure S6. Images of the most stable complexes of **3d** (a) and **4c** (b) with PRLm, as found in the molecular docking studies; Figure S7. Images of the most stable complexes of **1a** (a), **1b** (b), **1c** (c), **1d** (d), **1e** (e), and **1f** (f) with PRTb, as found in the molecular docking studies. Figure S8. Images of the most stable complexes of **1g** (a), **1i** (b), **1h** (c), **2a** (d), **2b** (e), and **2c** (f) with PRTb, as found in the molecular docking studies; Figure S9. Images of the most stable complexes of **2d** (a), **2e** (b), **2f** (c), **3a** (d), **3b** (e), and **3c** (f) with PRTb, as found in the molecular docking studies; Figure S10. Images of the most stable complexes of **3d** (a), **4c** (b) with PRTb, as found in the molecular docking studies; Figure S11. Adimensional affinities towards the amino acid residues in PRLm impacting the antileishmanial activity of BODIPY derivatives; Table S2. Population of each binding mode for each complex PRLm-BODIPY derivative complex; Table S3. Population of each binding mode for each complex PRTb-BODIPY derivative; Configuration of Autodock Vina used in the docking studies; Python Scripts.
