# Supplementary material for: Designing Antitrypanosomal and Antileishmanial BODIPY Derivatives: A Computational and In Vitro Assessment
Source: Molecules. 2024 Apr 30;29(9):2072. doi: 10.3390/molecules29092072 (PMC11085077; doi:10.3390/molecules29092072)
Supplement: Supplementary file 1 [file molecules-29-02072-s001.zip › Supporting information_Main File.pdf]

Supporting Information

Designing Antitrypanosomal and Antileishmanial BODIPY Derivatives: A Computational and in vitro Assessment

Raquel C. R. Gonçalves[1,2]*, Filipe Teixeira[1]*, Pablo Peñalver[3], Susana P. G. Costa[1], Juan C. Morales[3], M. Manuela M. Raposo[1]**

[1]Centre of Chemistry, University of Minho, Campus de Gualtar, 4710-057 Braga, Portugal.

[2]Advanced (Magnetic) Theranostic Nanostructures Lab, International Iberian Nanotechnology Laboratory, Av. Mestre José Veiga s/n, 4715-330 Braga, Portugal.

[3]Instituto de Parasitología y Biomedicina López Neyra, CSIC, PTS Granada, Avenida del Conocimiento, 17, 18016, Armilla, Granada, Spain.

*Co-first authors; **Corresponding author: mfox@quimica.uminho.pt

1. Synthesis and characterization of *meso*-substituted BODIPY derivatives **1f-i** 2

2. Synthesis and characterization of formylated BODIPY derivatives **2f, 3c** and **3d** 4

Figure S1. Analysis of the chemical structure - antitrypanosomal activity relationship according to the selectivity index (SI) values of the BODIPY derivatives... 6

Figure S2. Analysis of the chemical structure - antileishmanial activity relationship according to the selectivity index values of the BODIPY derivatives. 7

Table S1. Inter-compound distances based on difference counts of Morgan fingerprints of radius 2, normalized so that the maximum distance between the BODYPI derivatives reported in this work was 1.0.. 7

Figure S3. Images of the most stable complexes of **1a** (a), **1b** (b), **1c** (c), **1d** (d), **1e** (e), and **1f** (f) with PRLm, as found in the molecular docking studies. The side chains of the most relevant amino acid residues are explicitly shown (clockwise, starting at noon: Ser146, Tyr37, Ser40, Ala15, Arg17, Val230, Pro224 and Phe113). 8

Figure S4. Images of the most stable complexes of **1g** (a), **1i** (b), **1h** (c), **2a** (d), **2b** (e), and **2c** (f) with PRLm, as found in the molecular docking studies. The side chains of the most relevant amino acid residues are explicitly shown (clockwise, starting at noon: Ser146, Tyr37, Ser40, Ala15, Arg17, Val230, Pro224 and Phe113). 9

Figure S5. Images of the most stable complexes of **2d** (a), **2e** (b), **2f** (c), **3a** (d), **3b** (e), and **3c** (f) with PRLm, as found in the molecular docking studies. The side chains of the most relevant amino acid residues are explicitly shown (clockwise, starting at noon: Ser146, Tyr37, Ser40, Ala15, Arg17, Val230, Pro224 and Phe113). 10

Figure S6. Images of the most stable complexes of **3d** (a) and **4c** (b) with PRLm, as found in the molecular docking studies. The side chains of the most relevant amino acid residues are

explicitly shown (clockwise, starting at noon: Ser146, Tyr37, Ser40, Ala15, Arg17, Val230, Pro224 and Phe113). 11

Figure S7. Images of the most stable complexes of **1a** (a), **1b** (b), **1c** (c), **1d** (d), **1e** (e), and **1f** (f) with PRTb, as found in the molecular docking studies. The side chains of the most relevant amino acid residues are explicitly shown (clockwise, starting at noon: Thr126, Tyr34, Ser37, Ala12, Arg14, Pro210, Pro204, and Phe97). 12

Figure S8. Images of the most stable complexes of **1g** (a), **1i** (b), **1h** (c), **2a** (d), **2b** (e), and **2c** (f) with PRTb, as found in the molecular docking studies. The side chains of the most relevant amino acid residues are explicitly shown (clockwise, starting at noon: Thr126, Tyr34, Ser37, Ala12, Arg14, Pro210, Pro204, and Phe97). 12

Figure S9. Images of the most stable complexes of **2d** (a), **2e** (b), **2f** (c), **3a** (d), **3b** (e), and **3c** (f) with PRTb, as found in the molecular docking studies. The side chains of the most relevant amino acid residues are explicitly shown (clockwise, starting at noon: Thr126, Tyr34, Ser37, Ala12, Arg14, Pro210, Pro204, and Phe97). 13

Figure S10. Images of the most stable complexes of 3**d** (a), **4c** (b) with PRTb, as found in the molecular docking studies. The side chains of the most relevant amino acid residues are explicitly shown (clockwise, starting at noon: Thr126, Tyr34, Ser37, Ala12, Arg14, Pro210, Pro204, and Phe97). 14

Figure S11. Adimensional affinities towards the amino acid residues in PRLm impacting the antileishmanial activity of BODIPY derivatives 15

Table S2. Population of each binding mode for each complex PRLm-BODIPY derivative. 16

Table S3. Population of each binding mode for each complex PRTb-BODIPY derivative. 28

3. Configuration of Autodock Vina used in the docking studies 40

3.1 For PRLm (protein structure extracted from PDB 2HQX) 40

3.2 For PRTb (protein structure extracted from PDB 4CM7) 40

4. Python Scripts 41

4.1. Script for finding the 10 closest contacts 41

4.2. Script for performing Principal Component Analysis on the affinity data 43

5. References: 45

1. Synthesis and characterization of *meso*-substituted BODIPY derivatives 1f-i

2,4-Dimethylpyrrole (2.0 mmol) and aldehyde **f-i** (1.0 mmol) were dissolved in dry dichloromethane (100 mL). One drop of trifluoroacetic acid was added and the mixture was allowed to stir for 50 min at room temperature. A solution of DDQ (2.0 mmol) in dry dichloromethane (100 mL) was added to the mixture. The reaction was stirred for another 50 min and then triethylamine (16.3 mmol) was added. After stirring for 15 min, $BF_3.OEt_2$ (27.6 mmol) was added and further stirred for 30 min. The solvent was evaporated under reduced

pressure and the crude residue was purified by dry flash chromatography (petroleum ether/ethyl acetate, 4:1).

BODIPY derivative **1f** [1]

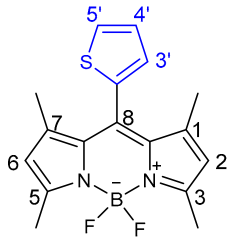

Yield = 8%; ^{1}H NMR (400 MHz, $CDCl_3$): δ = 1.59 (s, 6H, CH_3-1 and CH_3-7), 2.56 (s, 6H, CH_3-3 and CH_3-5), 6.01 (s, 2H, H-2 and H-6), 6.99 (dd, *J*= 1.2 and 3.6 Hz, 1H, H-3’), 7.14 (t, *J*= 3.6 Hz, 1H, H-4’), 7.51 (dd, *J*= 1.2 and 5.2 Hz, 1H, H-5’) ppm.

BODIPY derivative **1g** [2]

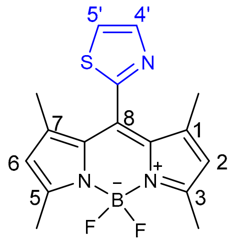

Yield = 7%; ^{1}H NMR (400 MHz, $CDCl_3$): δ =1.46 (s, 6H, CH_3-1 and CH_3-7), 2.56 (s, 6H, CH_3-3 and CH_3-5), 6.03 (s, 2H, H-2 and H-6), 7.64 (d, *J*= 3.2 Hz, 1H, H-5’), 8.02 (d, *J*= 3.6 Hz, 1H, H-4’) ppm; HRMS (ESI) *m/z* : $[M + 1]^{+\bullet}$ calcd for $C_{16}H_{17}BF_2N_3S$, 332.1199; found 332.1206.

BODIPY derivative **1h** [3]

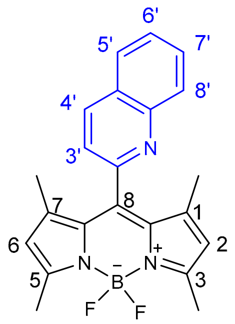

Yield = 37%; ^{1}H NMR (400 MHz, $CDCl_3$): δ =1.25 (s, 6H, CH_3-1 and CH_3-7), 2.56 (s, 6H, CH_3-3 and CH_3-5), 6.00 (s, 2H, H-2 and H-6), 7.60 (d, 1H, H-3'), 7.73 (dt, *J*= 1.2 and 8 Hz, 1H, H-6'), 7.88 (dt, *J*= 1.2 and 7.2 Hz, 3H, H-7'), 7.99 (d, *J*= 7.2 Hz, 1H, H-5'), 8.27 (d, *J*= 8.4 Hz, 1H, H-4'), 8.46 (d, *J*= 8.4 Hz, 1H, H-8') ppm; HRMS (ESI) *m/z*: [M + 1]$^{+\bullet}$ calcd for $C_{22}H_{21}BF_2N_3$, 376.1791; found 376.1797.

BODIPY derivative **1i**

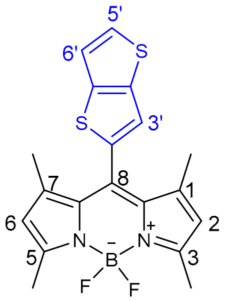

Yield = 3%; ^{1}H RMN (400 MHz, $CDCl_3$): δ =1.66 (s, 6H, CH_3-1 and CH_3-7), 2.57 (s, 6H, CH_3-3 and CH_3-5), 6.03 (s, 2H, H-2 and H-6), 7.19 (s, 1H, H-3'), 7.33 (dd, *J*= 0.8 and 5.2 Hz, 1H, H-5'), 7.46 (d, *J*= 5.2 Hz, 1H, H-6') ppm; ^{13}C NMR (100.6 MHz, $CDCl_3$): δ = 13.95 (CH_3-1 and CH_3-7), 14.68 (CH_3-3 and CH_3-5), 119.58 (C5'), 120.29 (C3'), 121.62 (C2 and C6), 127.95 (C6'), 132.45 (C8), 132.93 (C2'), 136.13 (C7a and C8a), 139.01 (C3'a), 140.28 (C6'a), 143.57 (C1 and C7), 156.45 (C3 and C5) ppm; HRMS (ESI) *m/z* : [M + 1]$^{+\bullet}$ calcd for $C_{19}H_{18}BF_2N_sS_2$, 387.0967; found 387.0965.

2. Synthesis and characterization of formylated BODIPY derivatives 2f, 3c and 3d

A mixture of *N,N*-dimethylformamide (23 mmol) and $POCl_3$ (18.2 mmol) was stirred for 5 min at 0 °C under N_2 atmosphere. The mixture was allowed to reach room temperature and stirred for 30 min. The BODIPY precursor **1f, 2c** or **1d** (0.127 mmol) dissolved in dichloroethane (7 mL) was added dropwise while stirring. The reaction mixture was heated for 2 h at 50 °C. After cooling, the solution was poured slowly into 40 mL of saturated sodium bicarbonate solution at 0 °C and stirred for 30 min at room temperature. Ethyl acetate (5 mL) was added to the reaction mixture and the resulting organic layer separated and washed with water (2 × 50 mL). The organic layer was dried with anhydrous $MgSO_4$ and filtered. After evaporation of the solvent to dryness, the crude residue was purified by a silica gel chromatography column, using dichloromethane as eluent.

BODIPY derivative **2f**

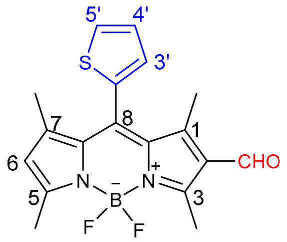

Yield = 49%; ^{1}H NMR (400 MHz, $CDCl_3$): δ = 1.66 (s, 3H, CH_3-7), 1.86 (s, 3H, CH_3-1), 2.63 (s, 3H, CH_3-5), 2.83 (s, 3H, CH_3-3), 6.19 (s, 1H, H-6), 7.03 (dd, *J*= 1.2 and 3.6 Hz, 1H, H-3'), 7.19 (dd, *J*= 3.6 and 5.2 Hz, 1H, H-4'), 7.58 (dd, *J*= 1.2 and 4.8 Hz, 1H, H-5'), 10.04 (s, 1H, CHO) ppm; ^{13}C NMR (100.6 MHz, $CDCl_3$): δ = 10.88 (CH_3-1), 13.05 (CH_3-3), 14.06 (CH_3-7), 15.18 (CH_3-5), 124.35 (C6), 126.44 (C2), 127.97 (C4'), 128.17 (C3' and C5'), 130.89 (C8a), 133.68 (C2'), 135.30 (C7a), 136.04 (C8), 143.20 (C1), 147.63 (C5), 156.89 (C3), 162.48 (C7), 185.95 (CHO) ppm.

BODIPY derivative **3c**

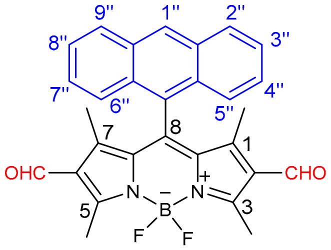

Yield = 10%; ^{1}H NMR (400 MHz, $CDCl_3$): δ = 1.02 (s, 6H, CH_3-1 and CH_3-7), 2.97 (s, 6H, CH_3-3 and CH_3-5), 7.49 (dt, *J*= 1.2 and 8 Hz, 2H, H-3' and H-8'), 7.56 (dt, *J*= 1.2 and 8.4 Hz, 2H, H- 4' and H-7'), 7.78 (dd, *J*= 0.8 and 8.8 Hz, 2H, H-2' and H-9'), 8.10 (d, *J*= 8.4 Hz, 2H, H-5' and H-6'), 8.69 (s, 1H, H-1'), 9.93 (s, 2H, C6-CHO and C2-CHO) ppm; HRMS (ESI) *m/z*: $[M + 1]^{+\bullet}$ calcd for $C_{29}H_{24}BF_2N_2O_2$, 481.1893; found 481.1905.

BODIPY derivative **3d**

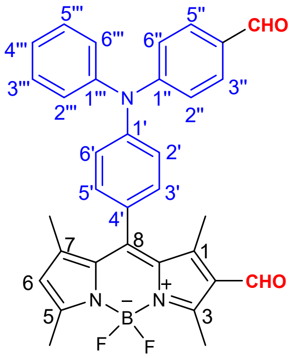

Yield = 26%; ^{1}H NMR (400 MHz, $CDCl_3$): δ =1.64 (s, 3H, CH_3-7), 1.86 (s, 3H, CH_3-1), 2.63 (s, 3H, CH_3-5), 2.82 (s, 3H, CH_3-3), 6.20 (s, 1H, H-6), 7.10 (d, *J*=8.8 Hz, 2H, H-2’’ and H-6’’), 7.18 (dd, *J*=1.2 and 8.4 Hz, 2H, H-3’’’ and H-5’’’), 7.23 (m, 3H, H-4’’’, H-2’ and H-6’), 7.32 (d, *J*=8.8 Hz, 2H, H-3’ and H-5’), 7.38 (m, 2H, H-2’’’ and H-6’’’), 7.75 (d, *J*=8.8 Hz, 2H, H-3’’ and H-5’’), 9.86 (s, 1H, C4’’-CHO), 10.04 (s, 1H, C2-CHO); ^{13}C NMR (100.6 MHz, $CDCl_3$): δ =11.81 (CH_3-1), 12.87 (CH_3-3), 15.05 (CH_3-5 and CH_3-7), 120.81 (C2’’ and C6’’), 124.17 (C6), 125.62 (C4’’’), 125.71 (C3’ and C5’), 126.34 (C2, C3’’’ and C5’’’), 129.19 (C2’ and C6’), 129.61 (C4’), 129.88 (C1), 130.03 (C2’’’ and C6’’’), 130.17 (C4’’), 131.37 (C3’’ and C5’’), 134.19 (C7), 142.41 (C8a), 142.98 (C8), 145.87 (C1’’’), 146.85 (C7a), 147.66 (C1’), 152.56 (C1’’), 156.61 (C3), 161.82 (C5), 185.88 (C2-CHO), 190.38 (C4’’-CHO); HRMS (ESI) *m/z*: $[M + 1]^{+\bullet}$ calcd for $C_{33}H_{29}BF_2N_3O_2$, 548.2315; found 548.2341.

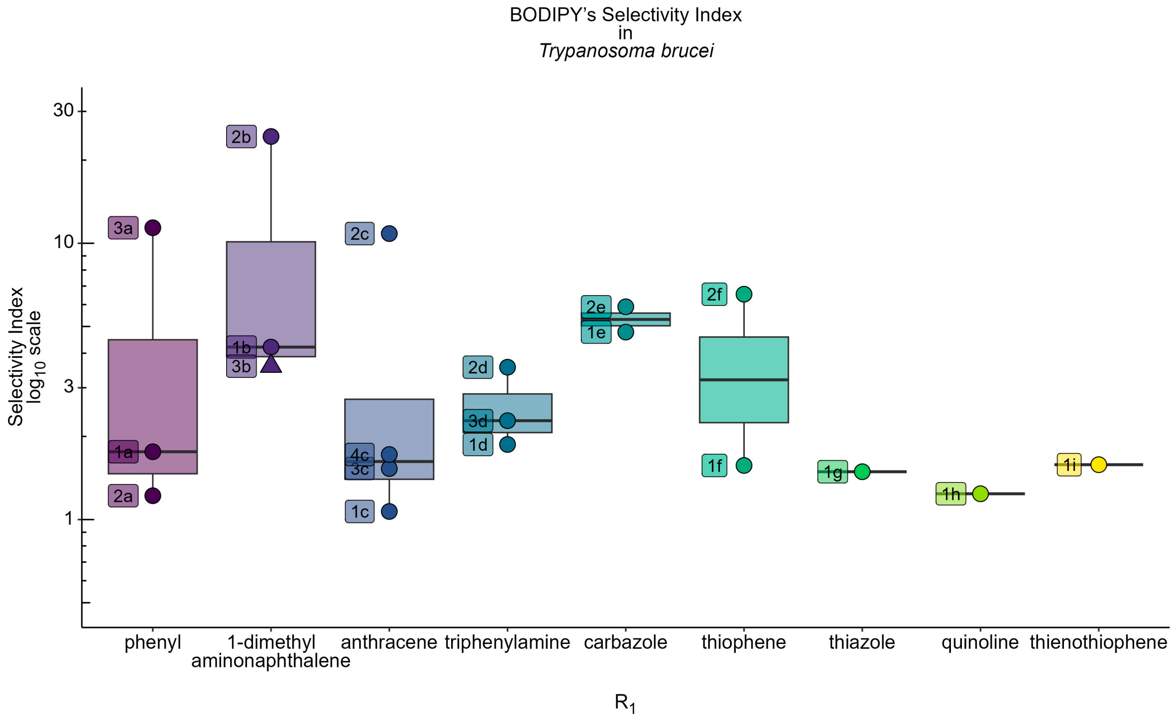

Figure S1. Analysis of the chemical structure - antitrypanosomal activity relationship according to the selectivity index (SI) values of the BODIPY derivatives. The BODIPYs were grouped based on the functionalization in position R_1 with different aromatic moieties and within each group the correspondent analogues functionalized in R_2 and/or R_3. The compounds labeled with circles correspond to their exact SI values. Compounds labeled with a triangle pointing up or down indicate that their SI values are greater or lower, respectively, than the marked value.

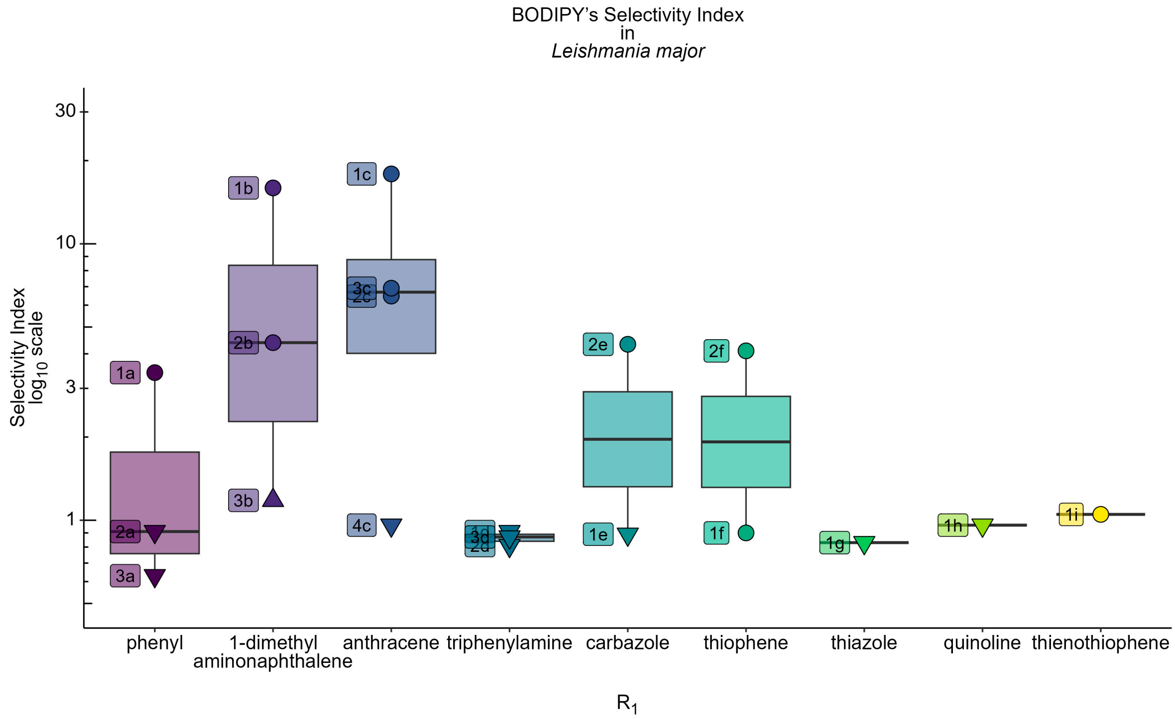

Figure S2. Analysis of the chemical structure - antileishmanial activity relationship according to the selectivity index values of the BODIPY derivatives. The BODIPYs were grouped based on the functionalization in position R_1 with different aromatic moieties and within each group the correspondent analogues functionalized in R_2 and/or R_3. The compounds labeled with circles correspond to their exact SI values. Compounds labeled with a triangle pointing up or down indicate that their SI values are greater or lower, respectively, than the marked value.

Table S1. Inter-compound distances based on difference counts of Morgan fingerprints of radius 2, normalized so that the maximum distance between the BODYPI derivatives reported in this work was 1.0.

Available in: Supplementary Materials_Table S1 (Excel file)

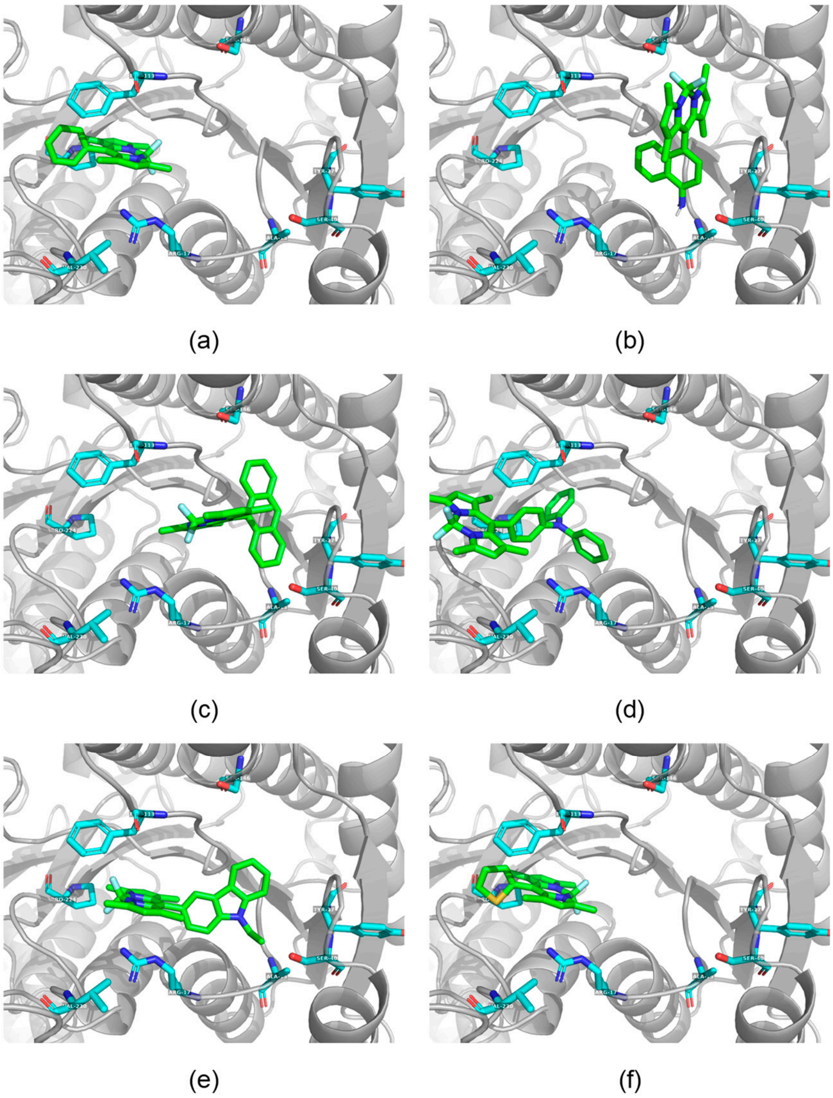

Figure S3. Images of the most stable complexes of **1a** (a), **1b** (b), **1c** (c), **1d** (d), **1e** (e), and **1f** (f) with PRLm, as found in the molecular docking studies. The side chains of the most relevant amino acid residues are explicitly shown (clockwise, starting at noon: Ser146, Tyr37, Ser40, Ala15, Arg17, Val230, Pro224 and Phe113).

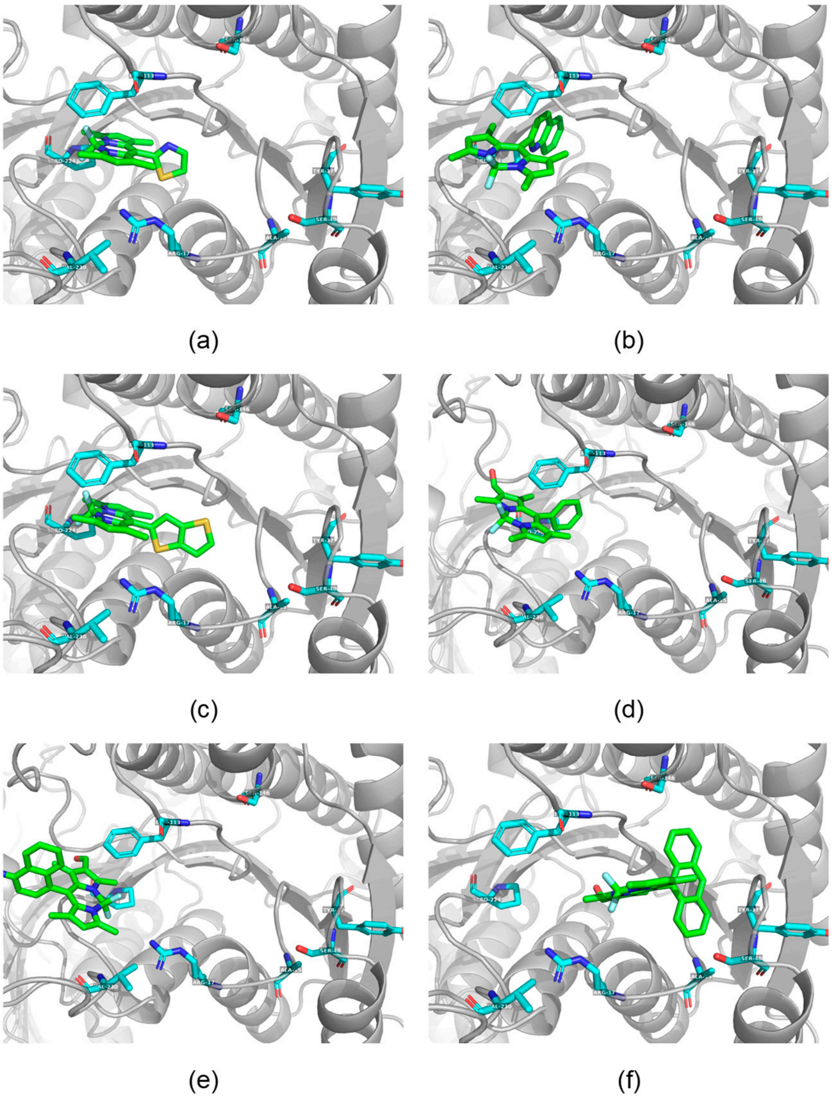

Figure S4. Images of the most stable complexes of **1g** (a), **1i** (b), **1h** (c), **2a** (d), **2b** (e), and **2c** (f) with PRLm, as found in the molecular docking studies. The side chains of the most relevant amino acid residues are explicitly shown (clockwise, starting at noon: Ser146, Tyr37, Ser40, Ala15, Arg17, Val230, Pro224 and Phe113).

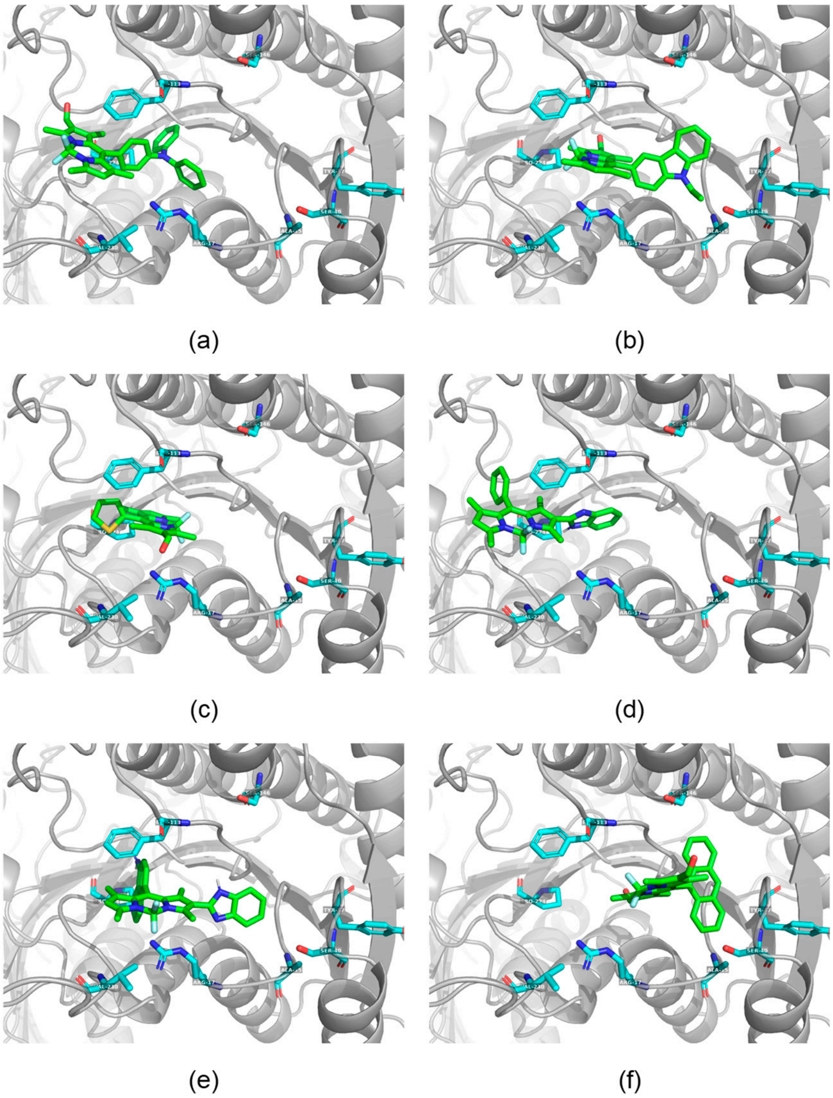

Figure S5. Images of the most stable complexes of **2d** (a), **2e** (b), **2f** (c), **3a** (d), **3b** (e), and **3c** (f) with PRLm, as found in the molecular docking studies. The side chains of the most relevant amino acid residues are explicitly shown (clockwise, starting at noon: Ser146, Tyr37, Ser40, Ala15, Arg17, Val230, Pro224 and Phe113).

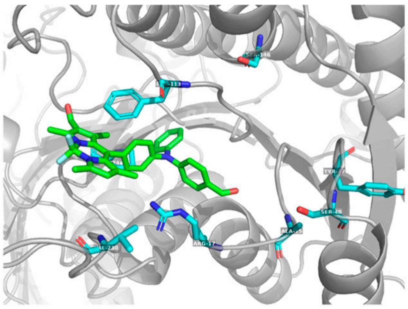

(a)

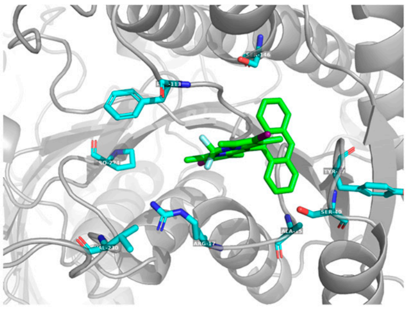

(b)

Figure S6. Images of the most stable complexes of **3d** (a) and **4c** (b) with PRLm, as found in the molecular docking studies. The side chains of the most relevant amino acid residues are explicitly shown (clockwise, starting at noon: Ser146, Tyr37, Ser40, Ala15, Arg17, Val230, Pro224 and Phe113).

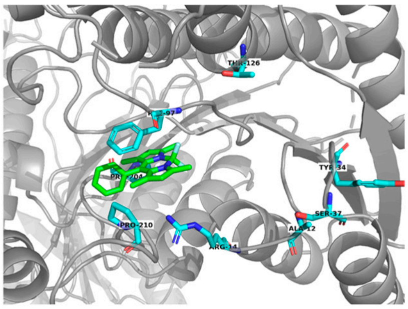

(a)

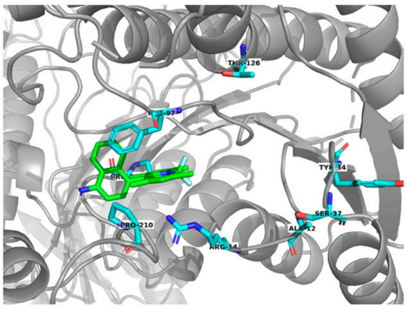

(b)

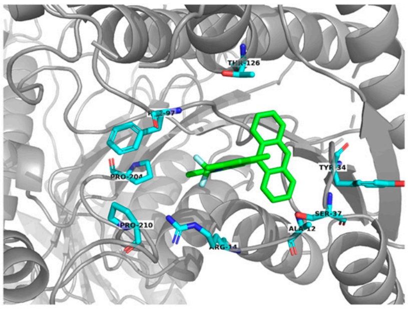

(c)

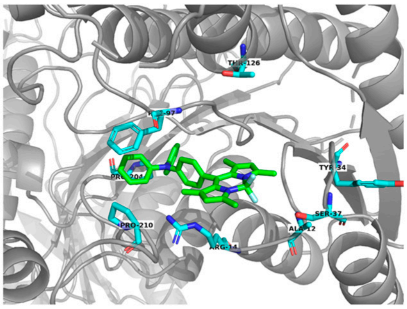

(d)

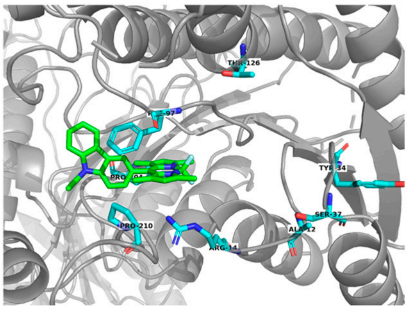

(e)

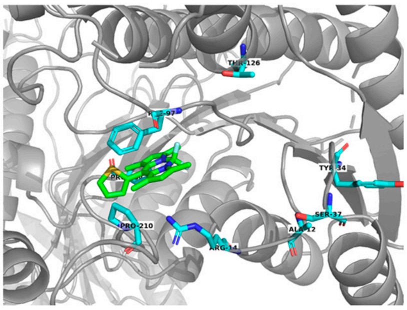

(f)

Figure S7. Images of the most stable complexes of **1a** (a), **1b** (b), **1c** (c), **1d** (d), **1e** (e), and **1f** (f) with PRTb, as found in the molecular docking studies. The side chains of the most relevant amino acid residues are explicitly shown (clockwise, starting at noon: Thr126, Tyr34, Ser37, Ala12, Arg14, Pro210, Pro204, and Phe97).

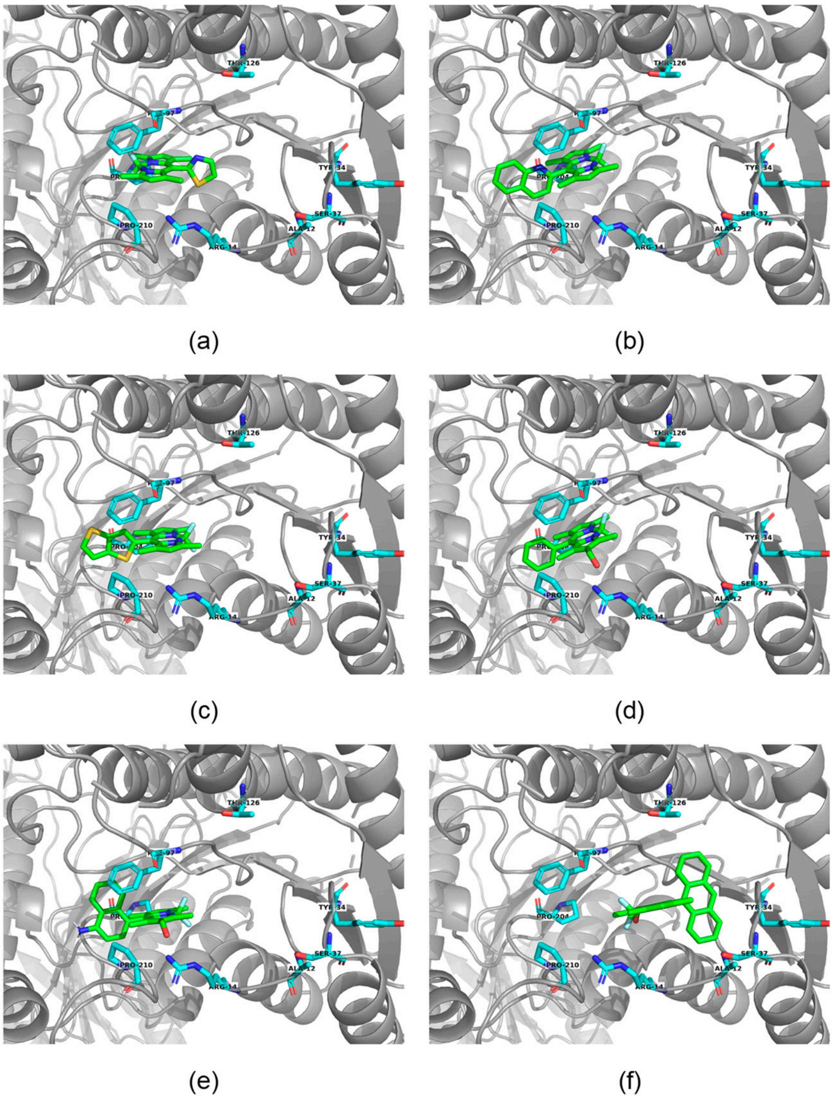

Figure S8. Images of the most stable complexes of **1g** (a), **1i** (b), **1h** (c), **2a** (d), **2b** (e), and **2c** (f) with PRTb, as found in the molecular docking studies. The side chains of the most relevant amino acid residues are explicitly shown (clockwise, starting at noon: Thr126, Tyr34, Ser37, Ala12, Arg14, Pro210, Pro204, and Phe97).

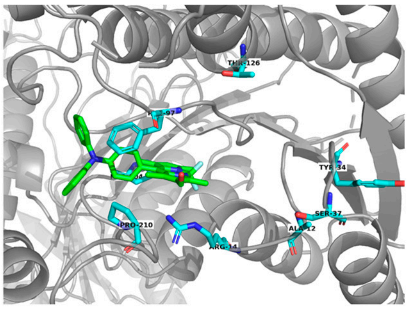

(a)

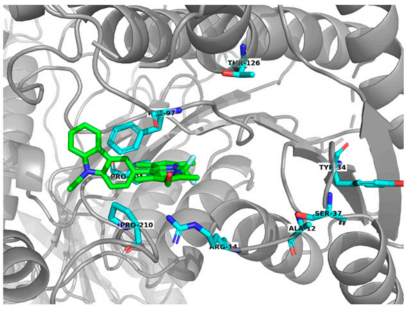

(b)

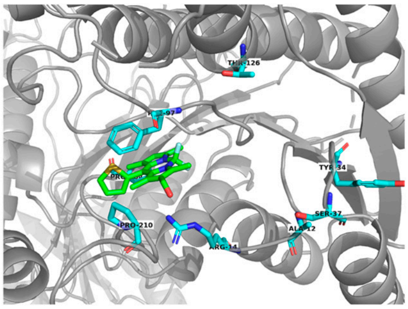

(c)

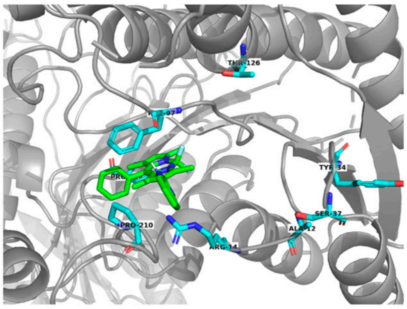

(d)

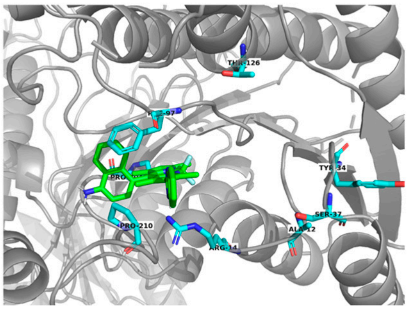

(e)

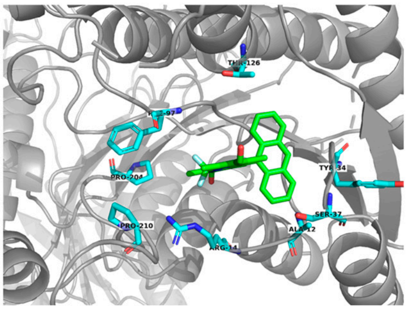

(f)

Figure S9. Images of the most stable complexes of **2d** (a), **2e** (b), **2f** (c), **3a** (d), **3b** (e), and **3c** (f) with PRTb, as found in the molecular docking studies. The side chains of the most relevant amino acid residues are explicitly shown (clockwise, starting at noon: Thr126, Tyr34, Ser37, Ala12, Arg14, Pro210, Pro204, and Phe97).

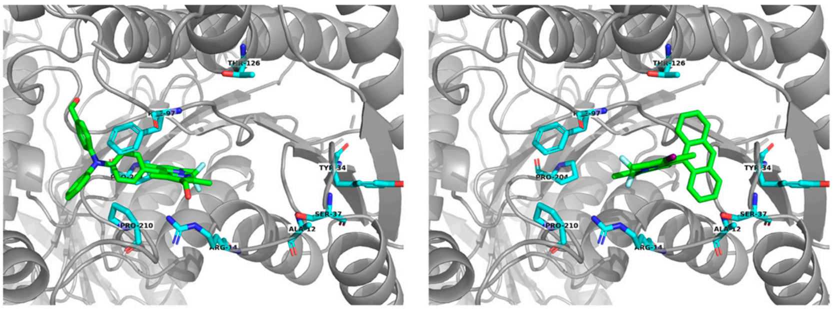

(a) (b)

Figure S10. Images of the most stable complexes of 3**d** (a), **4c** (b) with PRTb, as found in the molecular docking studies. The side chains of the most relevant amino acid residues are explicitly shown (clockwise, starting at noon: Thr126, Tyr34, Ser37, Ala12, Arg14, Pro210, Pro204, and Phe97).

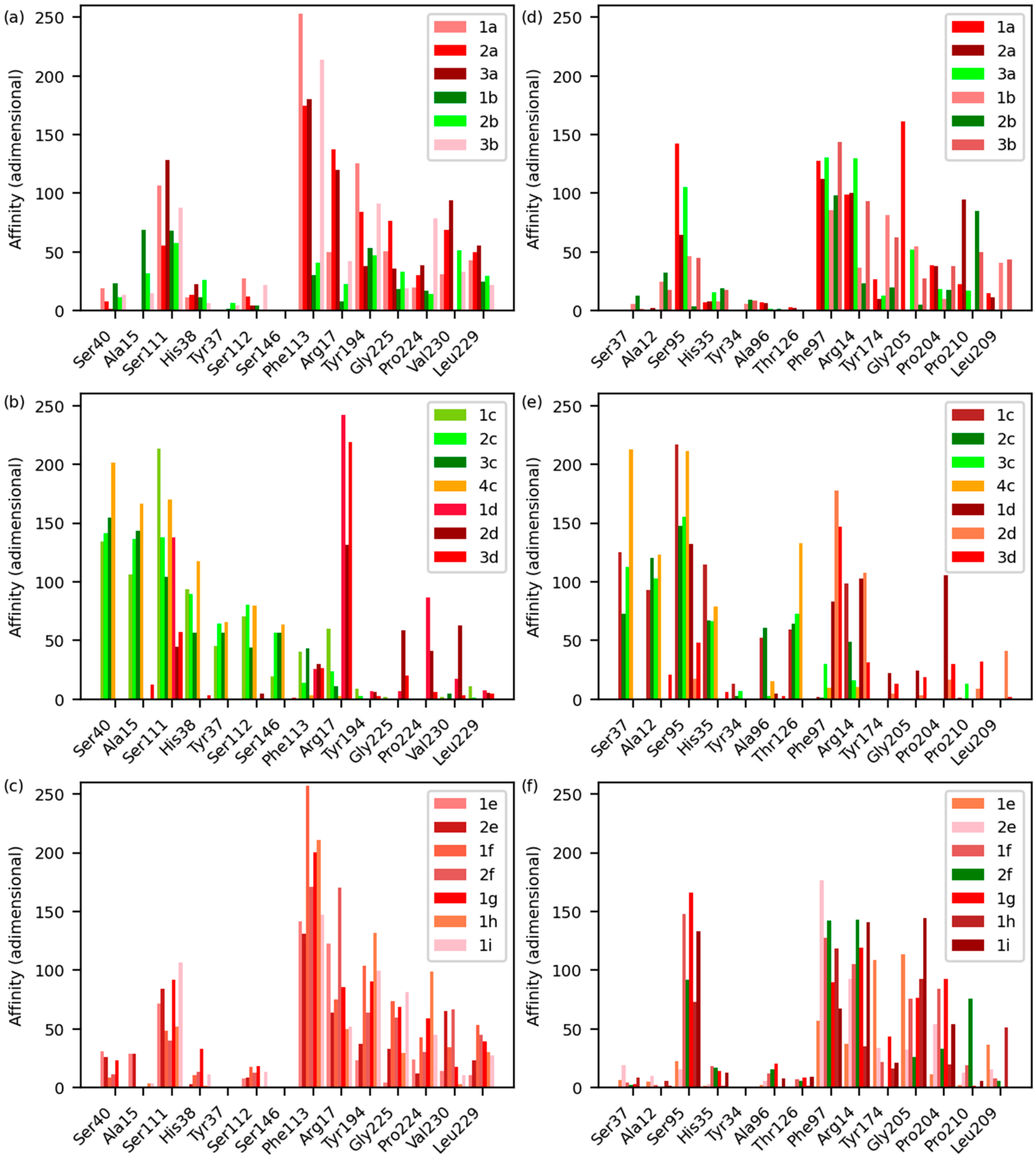

Figure S11. Adimensional affinities towards the amino acid residues in PRLm impacting the antileishmanial activity of BODIPY derivatives (according to the PCA analysis described in the main text): (a) compounds of the **a** and **b** series; (b) compounds of the **c** and **d** series; (c) compounds of the **e** , and **f** series, as well as **1g**–**1i**. Likewise, for the structural homologues in PRTb (d) compounds of the **a** and **b** series; (e) compounds of the **c** and **d** series; (f) compounds of the **e** , and **f** series, as well as **1g**–**1i**. Compounds showing antileishmanial activity are depicted in various shades of green in (a), (b) and (c), whereas those lacking antileishmanial activity are shown in various shades of red. A similar colour coding regarding antitrypanosomal activity is used in (d), (e) and (f).

Table S2. Population of each binding mode for each complex PRLm-BODIPY derivative.

Ligand	Mode	Affinity_ kcal.mol^{-1}	rmsd_L	rmsd_U	Z	%Pop
**1a**	1	-35.1104	0.000	0.000	1.00000	6.9
**1a**	2	-35.1062	2.114	6.324	0.99833	6.9
**1a**	3	-35.0979	2.112	5.099	0.99501	6.9
**1a**	4	-35.0813	0.048	3.916	0.98839	6.9
**1a**	5	-34.2992	4.339	7.514	0.72237	5.0
**1a**	6	-34.2534	4.344	6.773	0.70924	4.9
**1a**	7	-34.1411	3.295	6.452	0.67801	4.7
**1a**	8	-34.0954	3.205	5.642	0.66569	4.6
**1a**	9	-34.0787	10.466	12.322	0.66126	4.6
**1a**	10	-34.0662	10.464	11.913	0.65796	4.6
**1a**	11	-33.9539	3.320	6.060	0.62899	4.4
**1a**	12	-33.9373	3.456	5.726	0.62481	4.3
**1a**	13	-33.2758	1.660	2.593	0.47927	3.3
**1a**	14	-33.0470	4.727	7.727	0.43726	3.0
**1a**	15	-33.0262	4.732	6.811	0.43363	3.0
**1a**	16	-32.6227	4.382	7.207	0.36886	2.6
**1a**	17	-32.5437	4.386	6.518	0.35736	2.5
**1a**	18	-32.2858	2.769	5.092	0.32225	2.2
**1a**	19	-32.1360	2.738	6.144	0.30347	2.1
**1a**	20	-32.0736	2.476	5.992	0.29598	2.1
**1a**	21	-32.0486	2.645	4.820	0.29303	2.0
**1a**	22	-31.7824	1.869	4.888	0.26336	1.8
**1a**	23	-31.6202	1.874	5.482	0.24678	1.7
**1a**	24	-31.2250	2.437	5.000	0.21062	1.5
**1a**	25	-31.1459	1.978	3.107	0.20405	1.4
**1a**	26	-30.9670	2.782	5.020	0.18993	1.3
**1a**	27	-30.7590	2.312	6.933	0.17473	1.2
**1a**	28	-30.7466	3.267	5.575	0.17386	1.2
**1a**	29	-30.5885	3.577	5.780	0.16318	1.1
**1a**	30	-30.3056	10.605	11.708	0.14569	1.0
**1b**	1	-39.7197	0.000	0.000	1.00000	9.0
**1b**	2	-39.5949	0.205	3.565	0.95120	8.6
**1b**	3	-39.1622	14.156	16.671	0.79973	7.2
**1b**	4	-39.1414	14.110	16.376	0.79309	7.1
**1b**	5	-38.7421	10.562	13.010	0.67575	6.1
**1b**	6	-38.6256	8.494	11.196	0.64492	5.8
**1b**	7	-38.5882	11.029	13.463	0.63531	5.7
**1b**	8	-38.4717	8.487	11.245	0.60633	5.5
**1b**	9	-38.0682	7.420	9.800	0.51576	4.6
**1b**	10	-37.8269	6.970	10.013	0.46821	4.2
**1b**	11	-37.5814	8.919	11.081	0.42433	3.8
**1b**	12	-37.4067	9.660	11.998	0.39563	3.6
**1b**	13	-37.3318	8.918	11.715	0.38393	3.5
**1b**	14	-37.0074	8.907	12.037	0.33710	3.0
**1b**	15	-36.9408	6.013	8.709	0.32822	3.0
**1b**	16	-36.4624	3.836	6.679	0.27094	2.4

**1b**	17	-36.4166	3.831	6.198	0.26601	2.4
**1b**	18	-35.6096	9.023	11.582	0.19248	1.7
**1b**	19	-35.4432	10.691	12.882	0.18006	1.6
**1b**	20	-35.4058	8.131	10.623	0.17738	1.6
**1b**	21	-34.9440	3.145	6.060	0.14740	1.3
**1b**	22	-34.6611	8.777	11.254	0.13160	1.2
**1b**	23	-34.5363	1.730	4.203	0.12517	1.1
**1b**	24	-34.4490	13.884	16.155	0.12087	1.1
**1b**	25	-34.3616	7.337	9.672	0.11671	1.1
**1b**	26	-34.3574	4.579	7.360	0.11651	1.0
**1b**	27	-33.4922	2.455	5.505	0.08236	0.7
**1b**	28	-33.3923	2.457	5.974	0.07913	0.7
**1b**	29	-33.3507	16.612	18.598	0.07782	0.7
**1b**	30	-32.9014	3.059	5.114	0.06499	0.6
**1c**	1	-40.1898	0.000	0.000	1.00000	13.8
**1c**	2	-40.1731	0.042	3.391	0.99335	13.7
**1c**	3	-40.1565	0.017	4.677	0.98675	13.6
**1c**	4	-40.1315	0.047	3.221	0.97692	13.5
**1c**	5	-36.2586	1.977	5.234	0.20679	2.9
**1c**	6	-36.2502	1.987	4.438	0.20610	2.8
**1c**	7	-36.2211	1.977	4.569	0.20371	2.8
**1c**	8	-36.2170	1.989	5.128	0.20337	2.8
**1c**	9	-35.8550	2.644	6.296	0.17590	2.4
**1c**	10	-35.7760	2.637	6.367	0.17042	2.4
**1c**	11	-35.7594	2.653	5.918	0.16928	2.3
**1c**	12	-35.6720	14.205	16.787	0.16346	2.3
**1c**	13	-35.6637	14.175	16.989	0.16291	2.2
**1c**	14	-35.6138	3.267	7.402	0.15968	2.2
**1c**	15	-35.6096	3.274	7.867	0.15942	2.2
**1c**	16	-35.0022	2.408	5.519	0.12497	1.7
**1c**	17	-34.8899	1.960	4.046	0.11946	1.6
**1c**	18	-34.8816	1.953	3.274	0.11907	1.6
**1c**	19	-34.8026	1.968	4.896	0.11535	1.6
**1c**	20	-34.7734	2.421	5.226	0.11401	1.6
**1c**	21	-34.5738	2.439	4.730	0.10524	1.5
**1c**	22	-34.4947	2.438	5.122	0.10196	1.4
**1c**	23	-34.0538	1.871	4.550	0.08544	1.2
**1c**	24	-33.7293	4.995	7.771	0.07502	1.0
**1c**	25	-33.7168	4.879	7.271	0.07464	1.0
**1c**	26	-33.6294	4.956	8.095	0.07207	1.0
**1c**	27	-32.8557	2.687	6.424	0.05285	0.7
**1c**	28	-32.8432	2.680	6.435	0.05259	0.7
**1c**	29	-32.7392	2.681	6.703	0.05044	0.7
**1c**	30	-32.6435	2.696	5.680	0.04854	0.7
**1d**	1	-44.9904	0.000	0.000	1.00000	17.4
**1d**	2	-44.8906	0.131	3.153	0.96076	16.7
**1d**	3	-44.8614	0.166	4.418	0.94961	16.5
**1d**	4	-44.8323	0.080	3.096	0.93859	16.3
**1d**	5	-40.4310	4.861	9.489	0.16075	2.8
**1d**	6	-40.4019	1.450	4.649	0.15889	2.8

**1d**	7	-40.3770	4.862	9.708	0.15731	2.7
**1d**	8	-40.2979	1.448	3.493	0.15240	2.6
**1d**	9	-40.1274	4.864	9.633	0.14233	2.5
**1d**	10	-40.0733	4.868	9.460	0.13927	2.4
**1d**	11	-38.7130	3.185	8.794	0.08073	1.4
**1d**	12	-38.7130	4.835	9.186	0.08073	1.4
**1d**	13	-38.5715	3.167	8.304	0.07628	1.3
**1d**	14	-38.5133	3.171	9.283	0.07452	1.3
**1d**	15	-38.4384	4.841	9.472	0.07231	1.3
**1d**	16	-38.3968	3.233	6.001	0.07112	1.2
**1d**	17	-38.3885	3.168	8.814	0.07088	1.2
**1d**	18	-38.3677	3.278	6.533	0.07029	1.2
**1d**	19	-38.2970	3.270	6.400	0.06833	1.2
**1d**	20	-38.1888	3.246	5.902	0.06543	1.1
**1d**	21	-37.0573	4.440	8.887	0.04157	0.7
**1d**	22	-36.7744	4.677	8.989	0.03711	0.6
**1d**	23	-36.6288	4.645	9.090	0.03501	0.6
**1d**	24	-36.1962	3.640	5.898	0.02943	0.5
**1d**	25	-36.1920	1.654	3.767	0.02938	0.5
**1d**	26	-35.6096	4.046	7.900	0.02326	0.4
**1d**	27	-35.1645	4.185	7.655	0.01946	0.3
**1d**	28	-35.1603	4.163	7.650	0.01943	0.3
**1d**	29	-35.1021	4.177	7.796	0.01898	0.3
**1d**	30	-35.0938	4.195	7.884	0.01892	0.3
**1e**	1	-40.2730	0.000	0.000	1.00000	7.3
**1e**	2	-40.2438	0.065	3.339	0.98839	7.2
**1e**	3	-39.6448	2.967	5.800	0.77737	5.7
**1e**	4	-39.4035	8.035	13.741	0.70570	5.2
**1e**	5	-39.3494	8.015	14.206	0.69056	5.1
**1e**	6	-39.3370	2.975	4.947	0.68712	5.0
**1e**	7	-39.2122	8.060	13.380	0.65358	4.8
**1e**	8	-39.1664	8.018	13.870	0.64170	4.7
**1e**	9	-39.0582	6.695	9.154	0.61447	4.5
**1e**	10	-39.0541	6.704	9.534	0.61345	4.5
**1e**	11	-38.8461	3.816	7.241	0.56437	4.1
**1e**	12	-38.7338	3.822	6.580	0.53952	3.9
**1e**	13	-38.0058	2.634	5.372	0.40295	2.9
**1e**	14	-37.9725	9.697	14.287	0.39761	2.9
**1e**	15	-37.9059	5.153	8.806	0.38714	2.8
**1e**	16	-37.9059	9.675	14.066	0.38714	2.8
**1e**	17	-37.8726	3.181	8.467	0.38201	2.8
**1e**	18	-37.8602	5.384	8.683	0.38010	2.8
**1e**	19	-37.7104	3.190	8.007	0.35795	2.6
**1e**	20	-37.4608	3.278	7.677	0.32387	2.4
**1e**	21	-37.2070	2.847	5.177	0.29254	2.1
**1e**	22	-37.1530	3.383	8.143	0.28627	2.1
**1e**	23	-36.7078	2.905	4.201	0.23948	1.8
**1e**	24	-36.6621	3.084	7.271	0.23513	1.7
**1e**	25	-36.6038	3.192	8.243	0.22970	1.7
**1e**	26	-36.3584	4.529	7.180	0.20817	1.5

**1e**	27	-36.1629	4.274	10.083	0.19248	1.4
**1e**	28	-36.0381	4.689	7.437	0.18309	1.3
**1e**	29	-35.6138	10.073	14.374	0.15445	1.1
**1e**	30	-35.5098	14.320	19.555	0.14814	1.1
**1f**	1	-33.6003	0.000	0.000	1.00000	8.6
**1f**	2	-33.5670	0.117	4.017	0.98675	8.5
**1f**	3	-32.9805	2.493	4.810	0.77997	6.7
**1f**	4	-32.9139	2.489	6.158	0.75943	6.6
**1f**	5	-31.9987	4.473	6.862	0.52619	4.5
**1f**	6	-31.9571	4.494	7.621	0.51749	4.5
**1f**	7	-31.9280	3.252	6.164	0.51148	4.4
**1f**	8	-31.7990	10.500	12.198	0.48571	4.2
**1f**	9	-31.7782	1.488	4.224	0.48167	4.2
**1f**	10	-31.7283	3.362	5.473	0.47213	4.1
**1f**	11	-31.5744	1.501	2.533	0.44388	3.8
**1f**	12	-31.4746	3.240	5.572	0.42646	3.7
**1f**	13	-31.3622	3.229	5.864	0.40768	3.5
**1f**	14	-30.9920	3.358	5.455	0.35145	3.0
**1f**	15	-30.9046	4.537	7.327	0.33935	2.9
**1f**	16	-30.9046	4.519	6.644	0.33935	2.9
**1f**	17	-30.7424	4.268	7.461	0.31798	2.7
**1f**	18	-30.6966	4.696	6.598	0.31220	2.7
**1f**	19	-30.6342	4.706	7.610	0.30449	2.6
**1f**	20	-30.3680	4.133	6.834	0.27366	2.4
**1f**	21	-29.8979	2.362	4.572	0.22666	2.0
**1f**	22	-29.8896	4.092	6.606	0.22590	2.0
**1f**	23	-29.4445	4.803	7.397	0.18898	1.6
**1f**	24	-29.3072	4.260	6.544	0.17886	1.5
**1f**	25	-28.8538	3.331	5.832	0.14913	1.3
**1f**	26	-28.7706	6.649	9.361	0.14424	1.2
**1f**	27	-28.5667	2.937	6.929	0.13292	1.1
**1f**	28	-28.1299	7.163	9.679	0.11157	1.0
**1f**	29	-27.6474	21.178	22.601	0.09194	0.8
**1f**	30	-27.5933	21.166	22.911	0.08997	0.8
**1g**	1	-32.8598	0.000	0.000	1.00000	7.8
**1g**	2	-32.8598	0.143	4.008	1.00000	7.8
**1g**	3	-32.0154	2.501	6.113	0.71280	5.6
**1g**	4	-31.9946	2.513	4.760	0.70688	5.5
**1g**	5	-31.6077	2.423	5.881	0.60532	4.7
**1g**	6	-31.5869	9.721	11.285	0.60029	4.7
**1g**	7	-31.5744	2.440	4.679	0.59730	4.7
**1g**	8	-31.5411	9.668	10.792	0.58938	4.6
**1g**	9	-31.1709	4.192	6.610	0.50808	4.0
**1g**	10	-31.0128	3.496	6.237	0.47688	3.7
**1g**	11	-30.7424	3.628	6.306	0.42789	3.3
**1g**	12	-30.5968	2.860	4.597	0.40362	3.1
**1g**	13	-30.5594	4.640	7.986	0.39761	3.1
**1g**	14	-30.5094	4.586	8.112	0.38973	3.0
**1g**	15	-30.4678	4.101	7.077	0.38329	3.0
**1g**	16	-30.2973	1.968	3.001	0.35795	2.8

**1g**	17	-30.0810	2.956	5.553	0.32822	2.6
**1g**	18	-29.9062	5.545	9.446	0.30601	2.4
**1g**	19	-29.8189	2.058	4.577	0.29548	2.3
**1g**	20	-29.8022	1.677	2.699	0.29352	2.3
**1g**	21	-29.7898	3.435	5.226	0.29205	2.3
**1g**	22	-29.7690	4.906	8.291	0.28963	2.3
**1g**	23	-29.5734	3.379	6.334	0.26779	2.1
**1g**	24	-29.5318	5.549	8.721	0.26336	2.1
**1g**	25	-29.4861	3.471	6.475	0.25858	2.0
**1g**	26	-29.3779	2.043	3.863	0.24760	1.9
**1g**	27	-29.0368	10.171	11.237	0.21595	1.7
**1g**	28	-28.9786	3.327	5.500	0.21097	1.6
**1g**	29	-28.9037	6.581	9.692	0.20473	1.6
**1g**	30	-28.8954	2.801	5.021	0.20405	1.6
**1h**	1	-41.7123	0.000	0.000	1.00000	15.3
**1h**	2	-41.3629	0.259	3.631	0.86928	13.3
**1h**	3	-40.0275	3.357	5.454	0.50893	7.8
**1h**	4	-39.6448	2.943	6.042	0.43654	6.7
**1h**	5	-39.5366	3.007	6.711	0.41801	6.4
**1h**	6	-39.3869	1.950	3.149	0.39365	6.0
**1h**	7	-38.7088	1.960	4.634	0.29995	4.6
**1h**	8	-38.5882	1.258	3.959	0.28579	4.4
**1h**	9	-38.5590	2.965	5.190	0.28247	4.3
**1h**	10	-37.4816	2.923	7.707	0.18339	2.8
**1h**	11	-37.3485	4.248	6.682	0.17386	2.7
**1h**	12	-37.2736	3.098	8.337	0.16872	2.6
**1h**	13	-37.1904	4.305	6.468	0.16318	2.5
**1h**	14	-37.0573	1.898	2.643	0.15470	2.4
**1h**	15	-36.6205	5.313	7.908	0.12985	2.0
**1h**	16	-36.6038	1.938	4.022	0.12899	2.0
**1h**	17	-36.4000	3.009	8.418	0.11887	1.8
**1h**	18	-36.2336	4.120	6.674	0.11120	1.7
**1h**	19	-35.9258	5.683	8.434	0.09829	1.5
**1h**	20	-35.7469	1.284	1.918	0.09148	1.4
**1h**	21	-35.5139	5.390	7.731	0.08333	1.3
**1h**	22	-35.2810	3.348	7.819	0.07590	1.2
**1h**	23	-34.6154	2.292	4.266	0.05812	0.9
**1h**	24	-34.4282	1.927	2.454	0.05392	0.8
**1h**	25	-34.3574	2.631	6.647	0.05241	0.8
**1h**	26	-34.1702	3.301	7.936	0.04862	0.7
**1h**	27	-33.9082	9.997	12.676	0.04377	0.7
**1h**	28	-33.8998	3.290	8.639	0.04363	0.7
**1h**	29	-33.7584	20.179	22.003	0.04122	0.6
**1h**	30	-33.3590	20.243	22.104	0.03512	0.5
**1i**	1	-37.1155	0.000	0.000	1.00000	13.8
**1i**	2	-37.0365	0.024	3.766	0.96881	13.4
**1i**	3	-35.9299	4.204	6.280	0.62169	8.6
**1i**	4	-35.8259	4.297	6.935	0.59630	8.2
**1i**	5	-35.4890	3.868	5.715	0.52095	7.2
**1i**	6	-35.1229	3.208	6.231	0.44984	6.2

**1i**	7	-35.0896	3.203	7.194	0.44388	6.1
**1i**	8	-34.2576	1.301	4.102	0.31798	4.4
**1i**	9	-33.1178	6.434	10.478	0.20135	2.8
**1i**	10	-32.7184	6.358	10.887	0.17156	2.4
**1i**	11	-32.5894	8.421	10.137	0.16291	2.2
**1i**	12	-32.4854	3.748	6.487	0.15626	2.2
**1i**	13	-32.4688	3.739	7.146	0.15522	2.1
**1i**	14	-32.4397	3.667	6.793	0.15342	2.1
**1i**	15	-32.3315	4.505	7.443	0.14691	2.0
**1i**	16	-31.7616	2.139	3.056	0.11690	1.6
**1i**	17	-31.7200	1.652	2.203	0.11497	1.6
**1i**	18	-31.6118	2.123	4.451	0.11009	1.5
**1i**	19	-31.5411	3.387	7.265	0.10701	1.5
**1i**	20	-30.9379	2.941	4.394	0.08402	1.2
**1i**	21	-30.7424	6.839	9.883	0.07769	1.1
**1i**	22	-30.7382	9.402	11.009	0.07756	1.1
**1i**	23	-30.6675	6.926	10.025	0.07539	1.0
**1i**	24	-30.5261	9.296	11.151	0.07124	1.0
**1i**	25	-30.3597	3.599	6.744	0.06664	0.9
**1i**	26	-30.2266	4.687	7.480	0.06318	0.9
**1i**	27	-29.9853	5.458	9.335	0.05735	0.8
**1i**	28	-29.8646	6.639	9.658	0.05464	0.8
**1i**	29	-29.8230	6.696	9.565	0.05374	0.7
**1i**	30	-29.5485	2.319	3.009	0.04814	0.7
**2a**	1	-35.0147	0.000	0.000	1.00000	7.5
**2a**	2	-34.9232	3.960	6.727	0.96397	7.2
**2a**	3	-34.8400	4.526	7.633	0.93235	7.0
**2a**	4	-34.7318	1.205	1.706	0.89279	6.7
**2a**	5	-34.4822	4.172	6.459	0.80777	6.1
**2a**	6	-34.0579	2.389	4.858	0.68141	5.1
**2a**	7	-33.9622	2.877	5.861	0.65577	4.9
**2a**	8	-33.8874	2.217	5.714	0.63637	4.8
**2a**	9	-33.6586	2.813	5.205	0.58060	4.4
**2a**	10	-33.3632	12.687	14.323	0.51576	3.9
**2a**	11	-33.0886	2.127	3.253	0.46200	3.5
**2a**	12	-32.9805	2.208	4.656	0.44240	3.3
**2a**	13	-32.7226	12.651	13.870	0.39894	3.0
**2a**	14	-32.6810	3.063	7.017	0.39234	2.9
**2a**	15	-32.6310	4.572	7.215	0.38457	2.9
**2a**	16	-32.5853	2.008	4.948	0.37758	2.8
**2a**	17	-32.3814	4.338	6.326	0.34795	2.6
**2a**	18	-32.3814	2.498	5.089	0.34795	2.6
**2a**	19	-32.2816	3.170	5.544	0.33430	2.5
**2a**	20	-31.8323	3.970	6.578	0.27919	2.1
**2a**	21	-31.7782	3.303	6.775	0.27321	2.1
**2a**	22	-31.4954	2.086	4.225	0.24391	1.8
**2a**	23	-31.4704	4.074	6.888	0.24149	1.8
**2a**	24	-31.0378	2.148	3.404	0.20303	1.5
**2a**	25	-30.9587	3.812	6.046	0.19670	1.5
**2a**	26	-30.7382	5.456	7.901	0.18006	1.4

**2a**	27	-30.6509	3.328	5.491	0.17386	1.3
**2a**	28	-30.4595	6.341	9.523	0.16102	1.2
**2a**	29	-29.8730	4.235	7.095	0.12728	1.0
**2a**	30	-28.8829	9.859	12.360	0.08558	0.6
**2b**	1	-39.8528	0.000	0.000	1.00000	10.5
**2b**	2	-39.7571	2.795	7.053	0.96237	10.1
**2b**	3	-39.0166	13.861	16.595	0.71518	7.5
**2b**	4	-38.5923	13.680	16.059	0.60330	6.3
**2b**	5	-38.4717	5.441	9.655	0.57482	6.0
**2b**	6	-38.3344	3.209	7.899	0.54404	5.7
**2b**	7	-38.1347	2.025	4.309	0.50218	5.3
**2b**	8	-37.5981	4.882	7.931	0.40497	4.2
**2b**	9	-37.3984	6.457	9.259	0.37382	3.9
**2b**	10	-37.2861	3.234	7.449	0.35736	3.7
**2b**	11	-36.9741	4.926	9.730	0.31534	3.3
**2b**	12	-36.9075	3.830	7.879	0.30704	3.2
**2b**	13	-36.7245	5.438	8.458	0.28531	3.0
**2b**	14	-36.4125	11.886	15.071	0.25177	2.6
**2b**	15	-36.0339	5.085	8.434	0.21632	2.3
**2b**	16	-36.0339	10.226	14.041	0.21632	2.3
**2b**	17	-35.9674	10.339	14.107	0.21062	2.2
**2b**	18	-35.3267	5.568	9.086	0.16291	1.7
**2b**	19	-35.3101	5.277	7.306	0.16183	1.7
**2b**	20	-35.2394	5.789	8.610	0.15731	1.6
**2b**	21	-35.1146	10.290	14.199	0.14963	1.6
**2b**	22	-35.0314	5.068	9.277	0.14472	1.5
**2b**	23	-34.9357	8.266	12.233	0.13927	1.5
**2b**	24	-34.9232	4.986	8.881	0.13858	1.4
**2b**	25	-34.7194	12.872	15.486	0.12770	1.3
**2b**	26	-34.5821	6.581	9.058	0.12087	1.3
**2b**	27	-34.5280	5.132	9.257	0.11827	1.2
**2b**	28	-34.1994	5.263	7.960	0.10367	1.1
**2b**	29	-34.0870	11.612	14.807	0.09911	1.0
**2b**	30	-34.0704	5.625	7.728	0.09845	1.0
**2c**	1	-40.1814	0.000	0.000	1.00000	18.0
**2c**	2	-40.1398	0.056	3.126	0.98346	17.7
**2c**	3	-38.9085	1.920	4.140	0.60029	10.8
**2c**	4	-38.9043	1.927	5.192	0.59929	10.8
**2c**	5	-37.3360	2.440	4.817	0.31958	5.7
**2c**	6	-37.3277	2.435	5.405	0.31851	5.7
**2c**	7	-35.8717	2.784	5.305	0.17767	3.2
**2c**	8	-35.8301	2.278	5.159	0.17473	3.1
**2c**	9	-35.8176	2.780	4.977	0.17386	3.1
**2c**	10	-35.8093	2.283	4.515	0.17328	3.1
**2c**	11	-34.7859	3.058	6.907	0.11497	2.1
**2c**	12	-33.8915	3.010	6.594	0.08032	1.4
**2c**	13	-33.6253	3.165	6.521	0.07219	1.3
**2c**	14	-33.5712	3.206	5.762	0.07064	1.3
**2c**	15	-33.5254	3.163	6.792	0.06936	1.2
**2c**	16	-33.3549	14.178	17.157	0.06478	1.2

**2c**	17	-33.2509	14.152	16.969	0.06213	1.1
**2c**	18	-33.2301	3.207	6.076	0.06161	1.1
**2c**	19	-33.1635	4.558	6.767	0.05999	1.1
**2c**	20	-33.1344	4.551	7.210	0.05930	1.1
**2c**	21	-32.4189	2.724	7.009	0.04451	0.8
**2c**	22	-32.2816	1.980	2.278	0.04213	0.8
**2c**	23	-31.5786	17.999	21.992	0.03178	0.6
**2c**	24	-31.5078	4.782	7.426	0.03089	0.6
**2c**	25	-31.4995	2.591	5.858	0.03079	0.6
**2c**	26	-31.4662	2.536	5.159	0.03038	0.5
**2c**	27	-31.4538	17.933	22.138	0.03023	0.5
**2c**	28	-31.3914	2.547	5.558	0.02948	0.5
**2c**	29	-31.3581	2.617	5.119	0.02909	0.5
**2c**	30	-31.1002	3.087	5.641	0.02623	0.5
**2d**	1	-46.0595	0.000	0.000	1.00000	26.4
**2d**	2	-46.0346	0.058	2.939	0.99004	26.1
**2d**	3	-44.4371	1.773	3.921	0.52182	13.8
**2d**	4	-44.1626	1.771	4.840	0.46743	12.3
**2d**	5	-40.5184	2.270	4.363	0.10845	2.9
**2d**	6	-39.6406	5.327	9.681	0.07628	2.0
**2d**	7	-39.6074	5.345	9.925	0.07527	2.0
**2d**	8	-38.7712	3.620	6.299	0.05383	1.4
**2d**	9	-38.6838	3.627	6.223	0.05198	1.4
**2d**	10	-38.6173	3.810	9.471	0.05061	1.3
**2d**	11	-38.4134	3.806	9.050	0.04664	1.2
**2d**	12	-38.1555	3.443	6.551	0.04206	1.1
**2d**	13	-37.9850	3.435	6.470	0.03928	1.0
**2d**	14	-37.2653	5.335	9.978	0.02943	0.8
**2d**	15	-37.2445	5.302	9.713	0.02919	0.8
**2d**	16	-36.8534	5.276	9.070	0.02495	0.7
**2d**	17	-36.6995	5.282	9.113	0.02346	0.6
**2d**	18	-36.6995	3.808	9.019	0.02346	0.6
**2d**	19	-35.9507	4.849	9.207	0.01738	0.5
**2d**	20	-35.5347	4.836	8.865	0.01471	0.4
**2d**	21	-35.2061	4.135	7.666	0.01289	0.3
**2d**	22	-35.0938	4.125	7.714	0.01232	0.3
**2d**	23	-34.9690	1.624	2.344	0.01172	0.3
**2d**	24	-34.9232	5.071	8.810	0.01151	0.3
**2d**	25	-34.8358	3.789	6.580	0.01111	0.3
**2d**	26	-34.6486	5.162	9.019	0.01031	0.3
**2d**	27	-34.6154	3.984	6.341	0.01017	0.3
**2d**	28	-34.3075	4.647	8.552	0.00899	0.2
**2d**	29	-34.2118	3.975	6.206	0.00865	0.2
**2d**	30	-33.9456	3.954	6.892	0.00778	0.2
**2e**	1	-40.9094	0.000	0.000	1.00000	12.9
**2e**	2	-39.4618	4.580	7.552	0.55968	7.2
**2e**	3	-39.2621	4.354	7.621	0.51662	6.7
**2e**	4	-39.2330	1.923	4.095	0.51063	6.6
**2e**	5	-39.1498	3.857	6.732	0.49388	6.4
**2e**	6	-39.0416	8.472	13.900	0.47292	6.1

**2e**	7	-38.6838	6.853	9.741	0.40973	5.3
**2e**	8	-38.3011	8.587	14.278	0.35145	4.6
**2e**	9	-38.1722	2.465	4.033	0.33374	4.3
**2e**	10	-37.9933	3.165	5.764	0.31064	4.0
**2e**	11	-37.6522	3.242	7.777	0.27094	3.5
**2e**	12	-37.5565	9.935	14.458	0.26074	3.4
**2e**	13	-37.2195	3.674	8.598	0.22779	2.9
**2e**	14	-37.1696	6.820	9.201	0.22328	2.9
**2e**	15	-37.1613	3.929	8.428	0.22254	2.9
**2e**	16	-36.9990	3.565	8.192	0.20852	2.7
**2e**	17	-36.8160	2.548	3.811	0.19377	2.5
**2e**	18	-36.4998	3.601	8.055	0.17070	2.2
**2e**	19	-35.6262	3.028	7.455	0.12026	1.6
**2e**	20	-35.4682	6.835	9.588	0.11288	1.5
**2e**	21	-35.3974	9.609	14.221	0.10972	1.4
**2e**	22	-35.2394	3.972	8.157	0.10298	1.3
**2e**	23	-35.1853	3.316	8.256	0.10078	1.3
**2e**	24	-34.9731	14.405	19.433	0.09256	1.2
**2e**	25	-34.2992	14.259	19.160	0.07064	0.9
**2e**	26	-33.9414	3.514	8.515	0.06121	0.8
**2e**	27	-33.7584	6.639	9.168	0.05687	0.7
**2e**	28	-33.7501	4.328	7.607	0.05669	0.7
**2e**	29	-33.6502	3.552	9.003	0.05446	0.7
**2e**	30	-33.2966	4.500	6.924	0.04726	0.6
**2f**	1	-33.4339	0.000	0.000	1.00000	8.4
**2f**	2	-33.0678	2.437	4.628	0.86350	7.2
**2f**	3	-32.8931	4.800	7.878	0.80508	6.7
**2f**	4	-32.4896	3.793	6.580	0.68483	5.7
**2f**	5	-32.3440	3.678	6.271	0.64600	5.4
**2f**	6	-32.3357	2.267	4.782	0.64385	5.4
**2f**	7	-32.1194	4.404	6.712	0.59036	4.9
**2f**	8	-31.8240	3.243	6.314	0.52444	4.4
**2f**	9	-31.8032	5.429	8.273	0.52008	4.4
**2f**	10	-31.5786	3.317	5.577	0.47529	4.0
**2f**	11	-31.2000	10.165	11.268	0.40836	3.4
**2f**	12	-31.1834	4.373	6.459	0.40565	3.4
**2f**	13	-31.0336	1.571	2.421	0.38201	3.2
**2f**	14	-31.0170	10.393	11.390	0.37947	3.2
**2f**	15	-30.7590	4.094	6.320	0.34219	2.9
**2f**	16	-30.5011	2.963	4.792	0.30858	2.6
**2f**	17	-30.4678	3.041	4.931	0.30449	2.6
**2f**	18	-30.3181	2.593	4.669	0.28674	2.4
**2f**	19	-30.1891	2.843	4.268	0.27230	2.3
**2f**	20	-30.0768	4.397	6.404	0.26031	2.2
**2f**	21	-30.0518	4.747	6.498	0.25771	2.2
**2f**	22	-29.7482	4.808	7.567	0.22817	1.9
**2f**	23	-29.5069	4.546	7.107	0.20714	1.7
**2f**	24	-29.4029	3.862	6.243	0.19868	1.7
**2f**	25	-29.1866	5.576	8.711	0.18217	1.5
**2f**	26	-29.0410	2.377	3.367	0.17184	1.4

**2f**	27	-28.8995	3.189	5.781	0.16237	1.4
**2f**	28	-28.6998	6.278	9.534	0.14988	1.3
**2f**	29	-28.5750	6.530	9.114	0.14256	1.2
**2f**	30	-28.3920	4.679	7.425	0.13248	1.1
**3a**	1	-41.7830	0.000	0.000	1.00000	10.4
**3a**	2	-41.1923	4.096	7.283	0.78913	8.2
**3a**	3	-40.8304	2.197	4.464	0.68255	7.1
**3a**	4	-40.5059	3.378	5.573	0.59929	6.3
**3a**	5	-40.4144	4.734	8.285	0.57770	6.0
**3a**	6	-40.3062	2.260	4.680	0.55319	5.8
**3a**	7	-40.2605	2.656	5.001	0.54313	5.7
**3a**	8	-39.9984	2.756	8.780	0.48896	5.1
**3a**	9	-39.6240	2.598	3.799	0.42081	4.4
**3a**	10	-39.4243	1.972	4.508	0.38844	4.1
**3a**	11	-39.4035	3.428	8.424	0.38521	4.0
**3a**	12	-39.0582	5.516	9.624	0.33541	3.5
**3a**	13	-38.8045	2.358	5.217	0.30297	3.2
**3a**	14	-38.6672	3.693	7.207	0.28674	3.0
**3a**	15	-38.4634	3.499	5.890	0.26424	2.8
**3a**	16	-38.4301	5.915	11.135	0.26074	2.7
**3a**	17	-38.1347	2.864	9.189	0.23162	2.4
**3a**	18	-37.9392	2.474	3.692	0.21416	2.2
**3a**	19	-37.8768	3.730	7.898	0.20887	2.2
**3a**	20	-37.6022	4.213	9.359	0.18710	2.0
**3a**	21	-37.3984	2.689	4.160	0.17242	1.8
**3a**	22	-36.8826	2.393	5.050	0.14021	1.5
**3a**	23	-36.2794	3.175	5.432	0.11009	1.2
**3a**	24	-36.2586	2.768	8.220	0.10917	1.1
**3a**	25	-35.2810	10.504	14.624	0.07378	0.8
**3a**	26	-34.7526	3.722	5.647	0.05969	0.6
**3a**	27	-34.3990	3.803	6.467	0.05180	0.5
**3a**	28	-34.2826	4.850	8.884	0.04944	0.5
**3a**	29	-34.1744	4.606	6.726	0.04734	0.5
**3a**	30	-33.5421	6.788	10.123	0.03674	0.4
**3b**	1	-45.2733	0.000	0.000	1.00000	23.0
**3b**	2	-42.9312	2.803	3.334	0.39103	9.0
**3b**	3	-42.8938	2.793	8.141	0.38521	8.9
**3b**	4	-42.5027	3.040	4.482	0.32932	7.6
**3b**	5	-42.3197	2.995	8.069	0.30601	7.0
**3b**	6	-41.5958	3.023	7.836	0.22894	5.3
**3b**	7	-40.7763	3.480	9.279	0.16483	3.8
**3b**	8	-40.5309	6.634	10.429	0.14938	3.4
**3b**	9	-40.0982	2.408	6.535	0.12559	2.9
**3b**	10	-40.0442	2.940	8.235	0.12290	2.8
**3b**	11	-39.9859	3.546	6.743	0.12006	2.8
**3b**	12	-39.4576	2.165	6.271	0.09714	2.2
**3b**	13	-39.3370	3.321	9.047	0.09256	2.1
**3b**	14	-39.3370	2.844	8.606	0.09256	2.1
**3b**	15	-38.9376	3.223	6.771	0.07886	1.8
**3b**	16	-38.8960	5.385	8.676	0.07756	1.8

**3b**	17	-38.6630	4.021	8.903	0.07064	1.6
**3b**	18	-38.4176	2.623	6.046	0.06402	1.5
**3b**	19	-38.3843	3.659	7.212	0.06318	1.5
**3b**	20	-38.2970	3.901	9.708	0.06100	1.4
**3b**	21	-37.7603	4.559	8.113	0.04919	1.1
**3b**	22	-37.7395	2.628	5.608	0.04878	1.1
**3b**	23	-37.1363	3.184	7.306	0.03831	0.9
**3b**	24	-37.0198	8.619	12.963	0.03656	0.8
**3b**	25	-36.6163	5.235	8.988	0.03110	0.7
**3b**	26	-36.5622	5.488	9.255	0.03043	0.7
**3b**	27	-36.0714	3.453	6.116	0.02499	0.6
**3b**	28	-35.5597	5.042	8.475	0.02036	0.5
**3b**	29	-35.5430	3.483	9.159	0.02022	0.5
**3b**	30	-35.5222	12.279	15.416	0.02006	0.5
**3c**	1	-39.3578	0.000	0.000	1.00000	12.7
**3c**	2	-39.3411	0.057	4.702	0.99335	12.6
**3c**	3	-39.3037	0.059	5.598	0.97855	12.4
**3c**	4	-39.1706	0.084	3.038	0.92770	11.8
**3c**	5	-37.3360	2.558	4.885	0.44462	5.7
**3c**	6	-37.3152	2.551	5.448	0.44093	5.6
**3c**	7	-37.3110	2.540	4.957	0.44019	5.6
**3c**	8	-37.2819	2.550	5.514	0.43508	5.5
**3c**	9	-35.7261	3.092	8.119	0.23317	3.0
**3c**	10	-35.6720	3.057	7.358	0.22817	2.9
**3c**	11	-35.6054	3.058	6.878	0.22217	2.8
**3c**	12	-34.9190	2.807	4.925	0.16872	2.1
**3c**	13	-34.8234	2.805	6.109	0.16237	2.1
**3c**	14	-34.7984	2.818	5.807	0.16075	2.0
**3c**	15	-34.4323	2.818	5.323	0.13881	1.8
**3c**	16	-34.3866	3.029	8.499	0.13629	1.7
**3c**	17	-33.4797	2.607	5.176	0.09474	1.2
**3c**	18	-33.0803	2.507	5.687	0.08073	1.0
**3c**	19	-32.7475	12.016	15.688	0.07064	0.9
**3c**	20	-32.5354	11.969	15.538	0.06488	0.8
**3c**	21	-31.9904	4.980	7.169	0.05215	0.7
**3c**	22	-31.7741	2.094	5.111	0.04782	0.6
**3c**	23	-31.6368	24.173	27.331	0.04526	0.6
**3c**	24	-31.6285	24.195	27.287	0.04511	0.6
**3c**	25	-31.6035	24.196	27.154	0.04466	0.6
**3c**	26	-31.5370	24.183	27.100	0.04348	0.6
**3c**	27	-31.4454	26.206	28.929	0.04192	0.5
**3c**	28	-31.4288	17.614	21.976	0.04164	0.5
**3c**	29	-31.3830	17.597	21.846	0.04088	0.5
**3c**	30	-31.3206	4.105	7.935	0.03987	0.5
**3d**	1	-46.6170	0.000	0.000	1.00000	48.5
**3d**	2	-44.5494	1.747	3.838	0.43654	21.2
**3d**	3	-39.9901	4.775	9.639	0.07017	3.4
**3d**	4	-39.1498	3.475	6.089	0.05010	2.4
**3d**	5	-38.9293	3.640	6.381	0.04587	2.2
**3d**	6	-38.7462	3.477	9.472	0.04262	2.1

**3d**	7	-38.2595	3.360	6.311	0.03506	1.7
**3d**	8	-38.0848	3.610	6.639	0.03269	1.6
**3d**	9	-38.0307	4.733	9.703	0.03199	1.6
**3d**	10	-37.9891	3.920	6.277	0.03146	1.5
**3d**	11	-37.5648	3.467	9.381	0.02654	1.3
**3d**	12	-37.4982	3.190	9.886	0.02584	1.3
**3d**	13	-37.3526	3.726	6.478	0.02438	1.2
**3d**	14	-37.2486	3.720	6.010	0.02338	1.1
**3d**	15	-37.1488	4.369	9.108	0.02246	1.1
**3d**	16	-37.0198	3.620	6.324	0.02133	1.0
**3d**	17	-36.3210	4.198	6.930	0.01612	0.8
**3d**	18	-36.2128	4.058	7.491	0.01544	0.7
**3d**	19	-35.8550	4.360	7.712	0.01337	0.6
**3d**	20	-35.6179	4.472	6.267	0.01216	0.6
**3d**	21	-35.4390	4.877	8.888	0.01132	0.5
**3d**	22	-35.1936	10.460	14.212	0.01026	0.5
**3d**	23	-35.1437	4.907	8.654	0.01005	0.5
**3d**	24	-35.0314	3.817	5.943	0.00961	0.5
**3d**	25	-34.7194	4.093	6.968	0.00848	0.4
**3d**	26	-34.6778	4.686	7.889	0.00834	0.4
**3d**	27	-34.3616	4.367	7.419	0.00735	0.4
**3d**	28	-34.2950	4.529	6.784	0.00715	0.3
**3d**	29	-34.1827	4.926	10.039	0.00684	0.3
**3d**	30	-33.9040	4.523	7.571	0.00612	0.3
**4c**	1	-42.4986	0.000	0.000	1.00000	20.6
**4c**	2	-42.4819	0.031	3.126	0.99335	20.5
**4c**	3	-42.4778	0.057	4.207	0.99170	20.5
**4c**	4	-42.4778	0.010	5.241	0.99170	20.5
**4c**	5	-37.6147	1.180	1.238	0.14114	2.9
**4c**	6	-35.3226	2.801	5.459	0.05631	1.2
**4c**	7	-35.3018	2.830	5.145	0.05584	1.2
**4c**	8	-35.2726	2.809	5.111	0.05519	1.1
**4c**	9	-35.1562	2.670	4.752	0.05267	1.1
**4c**	10	-35.1104	2.796	4.771	0.05172	1.1
**4c**	11	-34.9274	2.595	5.357	0.04806	1.0
**4c**	12	-34.5571	1.813	3.622	0.04143	0.9
**4c**	13	-34.2742	2.593	4.726	0.03699	0.8
**4c**	14	-34.2202	2.602	4.762	0.03619	0.7
**4c**	15	-34.2202	2.596	5.356	0.03619	0.7
**4c**	16	-34.2160	2.600	5.390	0.03613	0.7
**4c**	17	-34.0496	1.978	5.480	0.03380	0.7
**4c**	18	-33.9622	2.723	5.347	0.03264	0.7
**4c**	19	-33.8624	2.077	4.647	0.03136	0.6
**4c**	20	-32.3107	4.120	6.818	0.01683	0.3
**4c**	21	-32.2816	4.117	7.479	0.01664	0.3
**4c**	22	-31.6368	2.417	5.109	0.01285	0.3
**4c**	23	-31.6160	2.434	3.203	0.01274	0.3
**4c**	24	-31.4538	17.576	22.024	0.01194	0.2
**4c**	25	-31.3747	17.578	21.899	0.01157	0.2
**4c**	26	-31.3539	17.587	21.586	0.01147	0.2

**4c**	27	-31.3539	17.627	21.733	0.01147	0.2
**4c**	28	-29.9853	2.774	5.731	0.00663	0.1
**4c**	29	-29.7315	2.784	7.279	0.00599	0.1
**4c**	30	-29.6026	2.857	6.312	0.00568	0.1

Table S3. Population of each binding mode for each complex PRTb-BODIPY derivative.

**Ligand**	**Mode**	**Affinity_ kcal.mol^{-1}**	**rmsd_L**	**rmsd_U**	**Z**	**%Pop**
**1a**	1	-39.4243	0.0000	0.0000	1.00000	19.3
**1a**	2	-39.3827	0.0410	3.9160	0.98346	19.0
**1a**	3	-36.8534	2.2090	6.3930	0.35676	6.9
**1a**	4	-36.8035	2.0330	4.6690	0.34969	6.7
**1a**	5	-36.7910	2.2030	5.2120	0.34795	6.7
**1a**	6	-36.7702	2.0230	3.3950	0.34506	6.7
**1a**	7	-36.0256	2.4040	5.0130	0.25600	4.9
**1a**	8	-36.0006	2.1160	4.9740	0.25345	4.9
**1a**	9	-35.9216	2.1140	5.9530	0.24555	4.7
**1a**	10	-34.3034	1.9980	5.2550	0.12835	2.5
**1a**	11	-34.2534	1.9420	5.7640	0.12580	2.4
**1a**	12	-32.9971	3.2730	5.5610	0.07602	1.5
**1a**	13	-32.8640	3.9460	6.6230	0.07207	1.4
**1a**	14	-32.8432	2.3600	3.6670	0.07147	1.4
**1a**	15	-32.8349	3.2870	6.4150	0.07124	1.4
**1a**	16	-32.5978	9.7630	12.0410	0.06478	1.2
**1a**	17	-32.2234	10.0110	11.7930	0.05575	1.1
**1a**	18	-31.7949	13.8210	16.5440	0.04695	0.9
**1a**	19	-31.7824	13.8230	16.8150	0.04671	0.9
**1a**	20	-31.4746	10.6100	12.1440	0.04129	0.8
**1a**	21	-31.0128	11.4610	12.9690	0.03431	0.7
**1a**	22	-30.9379	4.5810	8.9580	0.03330	0.6
**1a**	23	-30.6301	4.5380	7.4180	0.02943	0.6
**1a**	24	-30.1850	14.3250	17.0850	0.02462	0.5
**1a**	25	-30.1808	14.3440	17.3690	0.02458	0.5
**1a**	26	-29.9437	14.2550	17.1950	0.02235	0.4
**1a**	27	-29.8022	19.1170	22.0970	0.02112	0.4
**1a**	28	-29.7773	19.1260	21.8350	0.02091	0.4
**1a**	29	-29.5277	13.6940	16.2580	0.01892	0.4
**1a**	30	-29.4861	19.2740	22.2820	0.01861	0.4
**1b**	1	-44.1626	0.0000	0.0000	1.00000	21.9
**1b**	2	-43.8422	0.0600	3.5610	0.87948	19.3
**1b**	3	-42.2947	1.2220	2.3500	0.47292	10.4
**1b**	4	-41.8080	1.2160	4.2410	0.38908	8.5
**1b**	5	-40.7597	2.5570	6.1780	0.25557	5.6
**1b**	6	-40.3645	2.5430	6.8980	0.21813	4.8
**1b**	7	-39.5990	2.2020	4.5030	0.16049	3.5
**1b**	8	-39.1456	2.2160	3.3070	0.13381	2.9
**1b**	9	-38.7837	9.4580	12.4180	0.11574	2.5

**1b**	10	-38.7296	9.4240	12.1010	0.11325	2.5
**1b**	11	-38.5507	3.0260	4.4800	0.10542	2.3
**1b**	12	-38.2678	2.2450	3.6440	0.09411	2.1
**1b**	13	-38.1722	4.8750	9.1700	0.09057	2.0
**1b**	14	-37.8976	4.8520	9.7070	0.08113	1.8
**1b**	15	-36.6621	14.3250	17.1610	0.04944	1.1
**1b**	16	-36.5082	9.6850	12.3230	0.04648	1.0
**1b**	17	-36.3418	14.6960	17.1320	0.04348	1.0
**1b**	18	-36.1504	3.4810	5.0080	0.04027	0.9
**1b**	19	-35.3642	5.6050	10.6110	0.02938	0.6
**1b**	20	-35.3101	5.2940	10.9540	0.02875	0.6
**1b**	21	-35.1936	5.4680	10.5420	0.02744	0.6
**1b**	22	-35.0979	10.1950	12.4370	0.02641	0.6
**1b**	23	-35.0230	14.3460	16.6940	0.02563	0.6
**1b**	24	-34.9482	14.3800	16.3930	0.02487	0.5
**1b**	25	-34.7110	5.4230	8.4160	0.02261	0.5
**1b**	26	-34.6902	5.4450	7.8800	0.02243	0.5
**1b**	27	-34.2243	6.2320	9.5430	0.01861	0.4
**1b**	28	-34.1661	15.3360	17.6680	0.01818	0.4
**1b**	29	-34.1037	5.4140	10.2350	0.01773	0.4
**1b**	30	-33.9498	5.5390	10.9720	0.01667	0.4
**1c**	1	-40.7347	0.0000	0.0000	1.00000	13.0
**1c**	2	-40.7098	0.0450	3.3910	0.99004	12.9
**1c**	3	-40.6848	0.0440	3.2210	0.98019	12.8
**1c**	4	-40.6682	0.0570	4.6770	0.97367	12.7
**1c**	5	-37.3485	2.4440	4.8230	0.25729	3.4
**1c**	6	-37.3485	2.4440	5.4440	0.25729	3.4
**1c**	7	-37.2528	2.4360	5.2640	0.24760	3.2
**1c**	8	-37.2403	2.4340	4.6030	0.24637	3.2
**1c**	9	-36.8784	2.7610	5.9710	0.21309	2.8
**1c**	10	-36.8701	2.7440	6.3170	0.21238	2.8
**1c**	11	-36.8077	2.7540	6.6570	0.20714	2.7
**1c**	12	-36.7453	2.7310	6.3300	0.20202	2.6
**1c**	13	-36.1338	1.9780	4.4140	0.15809	2.1
**1c**	14	-35.9840	21.2140	24.4350	0.14888	1.9
**1c**	15	-35.9382	21.1930	24.4550	0.14618	1.9
**1c**	16	-35.8675	21.2100	24.6370	0.14209	1.9
**1c**	17	-35.8509	21.1950	24.5800	0.14114	1.8
**1c**	18	-35.8010	1.9920	4.5140	0.13835	1.8
**1c**	19	-35.0480	2.3020	5.3530	0.10230	1.3
**1c**	20	-35.0189	2.3290	6.0100	0.10111	1.3
**1c**	21	-34.9357	2.2750	4.8770	0.09780	1.3
**1c**	22	-34.6403	28.5390	31.4440	0.08687	1.1
**1c**	23	-34.5696	28.3890	31.2470	0.08445	1.1
**1c**	24	-34.4406	21.6420	25.2530	0.08019	1.0
**1c**	25	-34.4365	3.1190	6.4090	0.08006	1.0
**1c**	26	-34.4282	21.7640	25.2850	0.07979	1.0
**1c**	27	-34.3491	3.1210	6.9350	0.07730	1.0
**1c**	28	-34.3075	3.1660	6.3860	0.07602	1.0
**1c**	29	-34.2701	2.5440	4.8930	0.07489	1.0

**1c**	30	-33.8998	2.6720	5.4120	0.06456	0.8
**1d**	1	-42.6109	0.0000	0.0000	1.00000	8.6
**1d**	2	-42.4362	0.0950	3.1540	0.93235	8.0
**1d**	3	-42.3821	0.0650	2.9590	0.91235	7.9
**1d**	4	-42.3696	0.1120	4.3700	0.90780	7.8
**1d**	5	-41.9078	3.4280	8.9950	0.75438	6.5
**1d**	6	-41.6749	1.8060	4.0020	0.68712	5.9
**1d**	7	-41.6125	3.4120	8.8440	0.67014	5.8
**1d**	8	-41.5043	3.4220	9.3830	0.64170	5.5
**1d**	9	-41.0842	3.6540	8.4860	0.54223	4.7
**1d**	10	-39.4368	17.8990	23.1460	0.28013	2.4
**1d**	11	-39.4285	3.1580	9.2710	0.27919	2.4
**1d**	12	-39.3162	18.4910	24.2800	0.26690	2.3
**1d**	13	-39.2870	3.1950	8.2690	0.26380	2.3
**1d**	14	-39.2662	18.4760	24.1590	0.26161	2.3
**1d**	15	-39.2496	3.1990	8.7730	0.25987	2.2
**1d**	16	-39.1789	18.4500	24.3490	0.25261	2.2
**1d**	17	-39.1706	3.1890	8.8230	0.25177	2.2
**1d**	18	-39.1456	18.2170	23.6150	0.24926	2.1
**1d**	19	-39.1414	18.2310	23.5810	0.24884	2.1
**1d**	20	-38.9709	17.9480	23.1990	0.23240	2.0
**1d**	21	-38.6755	3.4830	8.8010	0.20645	1.8
**1d**	22	-38.6464	18.2690	23.5140	0.20405	1.8
**1d**	23	-38.6131	18.0040	23.2190	0.20135	1.7
**1d**	24	-38.5923	3.4870	8.6290	0.19967	1.7
**1d**	25	-38.4925	18.2770	23.4690	0.19184	1.7
**1d**	26	-38.0058	18.6100	24.3760	0.15783	1.4
**1d**	27	-37.9475	3.6700	8.2810	0.15419	1.3
**1d**	28	-37.7062	18.6320	24.4690	0.13997	1.2
**1d**	29	-37.6522	18.6730	24.5410	0.13697	1.2
**1d**	30	-37.2653	3.6110	8.8420	0.11729	1.0
**1e**	1	-47.9814	0.0000	0.0000	1.00000	27.1
**1e**	2	-47.9315	0.0980	3.3400	0.98019	26.6
**1e**	3	-46.3091	1.0340	2.5010	0.51148	13.9
**1e**	4	-45.8224	1.0240	4.1630	0.42081	11.4
**1e**	5	-42.0493	4.5120	10.7360	0.09271	2.5
**1e**	6	-41.7414	4.1590	7.1340	0.08195	2.2
**1e**	7	-41.7290	4.1910	6.7120	0.08154	2.2
**1e**	8	-41.4211	3.0780	8.1260	0.07207	2.0
**1e**	9	-41.3213	3.0830	8.6160	0.06924	1.9
**1e**	10	-40.9427	2.0690	3.8970	0.05949	1.6
**1e**	11	-40.4518	2.1170	5.1330	0.04887	1.3
**1e**	12	-39.3453	4.9370	10.6980	0.03136	0.8
**1e**	13	-38.8586	2.8630	7.9170	0.02580	0.7
**1e**	14	-38.8502	3.0900	9.1350	0.02571	0.7
**1e**	15	-38.8003	2.8730	8.4130	0.02520	0.7
**1e**	16	-38.7462	3.1090	8.7980	0.02466	0.7
**1e**	17	-37.4358	9.0380	12.6960	0.01458	0.4
**1e**	18	-37.4192	5.8990	9.7380	0.01449	0.4
**1e**	19	-37.3069	3.1980	8.3390	0.01385	0.4

**1e**	20	-37.1738	3.1900	8.7530	0.01313	0.4
**1e**	21	-36.8451	4.8610	8.2720	0.01151	0.3
**1e**	22	-36.7494	3.3730	9.1880	0.01108	0.3
**1e**	23	-36.7328	4.0260	7.6250	0.01100	0.3
**1e**	24	-36.2336	5.7960	8.4260	0.00901	0.2
**1e**	25	-36.2045	9.0220	12.7300	0.00890	0.2
**1e**	26	-36.0797	3.5930	8.9060	0.00847	0.2
**1e**	27	-35.4806	5.0520	10.9350	0.00666	0.2
**1e**	28	-35.2685	17.0300	19.4210	0.00612	0.2
**1e**	29	-35.0730	15.9490	18.2110	0.00566	0.2
**1e**	30	-34.8691	15.9640	18.0240	0.00521	0.1
**1f**	1	-36.1795	0.0000	0.0000	1.00000	11.9
**1f**	2	-36.0090	0.1410	4.0170	0.93391	11.1
**1f**	3	-35.1395	1.2280	1.4420	0.65906	7.9
**1f**	4	-35.1062	2.6370	4.9130	0.65032	7.8
**1f**	5	-35.0688	2.0670	3.2950	0.64063	7.6
**1f**	6	-34.7776	1.9900	4.5700	0.57004	6.8
**1f**	7	-34.7485	2.6380	6.2340	0.56343	6.7
**1f**	8	-34.3283	2.5270	4.7460	0.47608	5.7
**1f**	9	-33.4838	2.5020	5.5960	0.33935	4.1
**1f**	10	-33.4589	2.4500	4.7200	0.33597	4.0
**1f**	11	-32.6560	2.3810	5.5840	0.24351	2.9
**1f**	12	-32.2442	2.3510	4.8370	0.20645	2.5
**1f**	13	-32.0029	4.0280	6.1590	0.18741	2.2
**1f**	14	-31.6909	4.0200	6.5450	0.16538	2.0
**1f**	15	-31.4413	9.5820	11.7400	0.14963	1.8
**1f**	16	-31.3664	9.6170	11.4630	0.14520	1.7
**1f**	17	-31.0502	10.4980	12.1990	0.12792	1.5
**1f**	18	-30.9462	3.3550	5.7760	0.12269	1.5
**1f**	19	-30.7507	3.4530	7.0670	0.11344	1.4
**1f**	20	-30.4637	3.8430	5.8110	0.10111	1.2
**1f**	21	-30.3472	3.3800	6.2360	0.09650	1.2
**1f**	22	-30.2224	10.3170	11.7910	0.09179	1.1
**1f**	23	-29.4819	4.5070	7.4340	0.06821	0.8
**1f**	24	-29.3571	31.0210	33.3740	0.06488	0.8
**1f**	25	-29.2989	14.2680	17.1220	0.06339	0.8
**1f**	26	-28.9286	11.5600	12.6020	0.05464	0.7
**1f**	27	-28.9078	10.6770	11.5570	0.05419	0.6
**1f**	28	-28.8954	11.2180	12.1350	0.05392	0.6
**1f**	29	-28.7331	7.7230	10.0770	0.05052	0.6
**1f**	30	-28.6250	10.7000	11.9370	0.04838	0.6
**1g**	1	-35.2643	0.0000	0.0000	1.00000	11.0
**1g**	2	-35.2602	2.5400	5.8630	0.99833	10.9
**1g**	3	-35.2061	0.1870	4.0100	0.97692	10.7
**1g**	4	-35.0896	2.4840	4.7630	0.93235	10.2
**1g**	5	-34.8650	2.6990	6.2290	0.85205	9.3
**1g**	6	-34.4032	2.4760	4.6830	0.70806	7.8
**1g**	7	-33.0013	1.6640	2.5150	0.40362	4.4
**1g**	8	-32.9930	1.7600	4.3110	0.40228	4.4
**1g**	9	-32.6893	1.7240	4.2830	0.35617	3.9

**1g**	10	-32.0445	2.7600	6.0030	0.27503	3.0
**1g**	11	-31.8032	3.7820	6.8990	0.24968	2.7
**1g**	12	-31.1917	8.7010	10.6100	0.19539	2.1
**1g**	13	-31.1792	3.7700	6.3130	0.19442	2.1
**1g**	14	-31.1085	9.0050	11.1430	0.18898	2.1
**1g**	15	-30.8131	2.3050	3.2420	0.16788	1.8
**1g**	16	-30.3805	2.1070	4.8630	0.14114	1.5
**1g**	17	-30.2723	9.6210	11.5260	0.13516	1.5
**1g**	18	-30.1142	3.2200	6.1030	0.12686	1.4
**1g**	19	-29.7149	9.5240	11.4270	0.10809	1.2
**1g**	20	-29.5443	3.7960	6.2650	0.10094	1.1
**1g**	21	-28.8538	3.5460	5.9180	0.07653	0.8
**1g**	22	-28.8205	3.6430	6.2970	0.07552	0.8
**1g**	23	-28.8080	10.3470	10.9900	0.07514	0.8
**1g**	24	-28.3296	10.2790	11.1590	0.06203	0.7
**1g**	25	-28.2214	3.8180	5.5930	0.05939	0.7
**1g**	26	-28.0842	3.6420	6.3640	0.05621	0.6
**1g**	27	-28.0634	3.1160	5.5490	0.05575	0.6
**1g**	28	-27.9594	8.2840	10.4110	0.05347	0.6
**1g**	29	-27.9094	7.5710	9.8060	0.05241	0.6
**1g**	30	-27.8637	14.2800	17.2650	0.05146	0.6
**1h**	1	-47.3699	0.0000	0.0000	1.00000	29.2
**1h**	2	-47.2493	0.1450	3.6250	0.95279	27.8
**1h**	3	-45.1485	1.4940	4.1830	0.41041	12.0
**1h**	4	-44.7907	1.4930	2.1290	0.35557	10.4
**1h**	5	-40.7680	2.5020	7.5770	0.07088	2.1
**1h**	6	-40.6432	4.5450	7.2070	0.06742	2.0
**1h**	7	-40.4685	2.5120	6.8610	0.06286	1.8
**1h**	8	-40.4227	4.6800	7.0670	0.06172	1.8
**1h**	9	-39.7530	3.9190	6.8510	0.04718	1.4
**1h**	10	-39.5866	2.1050	3.3100	0.04414	1.3
**1h**	11	-39.1872	10.4910	12.6630	0.03761	1.1
**1h**	12	-38.7878	3.1290	6.1350	0.03204	0.9
**1h**	13	-38.7338	10.5900	12.8740	0.03136	0.9
**1h**	14	-38.5923	2.6330	6.8790	0.02963	0.9
**1h**	15	-38.5258	5.5570	10.2400	0.02885	0.8
**1h**	16	-38.4925	5.3940	10.5410	0.02847	0.8
**1h**	17	-37.8851	3.4360	6.0290	0.02231	0.7
**1h**	18	-37.7270	3.2240	5.4220	0.02094	0.6
**1h**	19	-37.4442	3.4280	5.4500	0.01870	0.5
**1h**	20	-37.3443	2.9900	8.3850	0.01796	0.5
**1h**	21	-36.2586	2.1680	6.7730	0.01162	0.3
**1h**	22	-36.1920	11.4080	13.5280	0.01132	0.3
**1h**	23	-35.9590	2.5970	6.7480	0.01031	0.3
**1h**	24	-35.9466	4.4160	7.9120	0.01026	0.3
**1h**	25	-35.7094	11.3290	13.8260	0.00933	0.3
**1h**	26	-35.6637	2.5640	7.2770	0.00916	0.3
**1h**	27	-35.2019	4.0940	8.7720	0.00761	0.2
**1h**	28	-35.1645	13.6030	16.0810	0.00750	0.2
**1h**	29	-34.7984	15.5180	17.7540	0.00647	0.2

**1h**	30	-34.4573	13.5180	15.6550	0.00565	0.2
**1i**	1	-40.6890	0.0000	0.0000	1.00000	22.9
**1i**	2	-40.4934	0.0900	3.7660	0.92461	21.2
**1i**	3	-38.1888	3.1060	6.0950	0.36702	8.4
**1i**	4	-37.9184	3.1070	7.0530	0.32932	7.6
**1i**	5	-36.0256	3.6350	6.1470	0.15419	3.5
**1i**	6	-35.8675	3.6890	5.5630	0.14472	3.3
**1i**	7	-35.6470	3.0730	5.7630	0.13248	3.0
**1i**	8	-35.5222	3.0600	6.5810	0.12601	2.9
**1i**	9	-35.2477	9.6060	11.4040	0.11288	2.6
**1i**	10	-35.1645	9.4920	11.6330	0.10917	2.5
**1i**	11	-34.5322	2.8930	5.9240	0.08473	1.9
**1i**	12	-34.5322	2.9740	4.3450	0.08473	1.9
**1i**	13	-34.5114	2.8760	5.1440	0.08402	1.9
**1i**	14	-34.4989	3.6180	6.0150	0.08361	1.9
**1i**	15	-34.4698	2.9050	6.5100	0.08263	1.9
**1i**	16	-34.1952	3.6190	5.6730	0.07402	1.7
**1i**	17	-33.8582	2.0860	3.1200	0.06467	1.5
**1i**	18	-33.8166	1.4540	1.8650	0.06360	1.5
**1i**	19	-33.7709	2.9830	5.1940	0.06244	1.4
**1i**	20	-32.8141	3.2860	6.0500	0.04255	1.0
**1i**	21	-32.0944	11.4010	12.9300	0.03188	0.7
**1i**	22	-31.7075	10.6150	11.8700	0.02730	0.6
**1i**	23	-31.6451	10.4130	11.8060	0.02663	0.6
**1i**	24	-31.4454	10.1260	11.1470	0.02458	0.6
**1i**	25	-31.2291	3.4270	6.5940	0.02254	0.5
**1i**	26	-31.0586	15.1610	17.9060	0.02105	0.5
**1i**	27	-31.0003	15.1750	17.8700	0.02056	0.5
**1i**	28	-30.9587	7.5430	9.6540	0.02022	0.5
**1i**	29	-30.7965	7.5640	9.6300	0.01895	0.4
**1i**	30	-30.7507	19.1540	21.7440	0.01861	0.4
**2a**	1	-39.1165	0.0000	0.0000	1.00000	26.5
**2a**	2	-37.0614	2.2960	5.0560	0.43873	11.6
**2a**	3	-36.2627	2.3670	4.7200	0.31851	8.4
**2a**	4	-36.1587	2.2500	4.6890	0.30550	8.1
**2a**	5	-35.8675	2.9040	6.2660	0.27184	7.2
**2a**	6	-35.3309	2.2280	5.1720	0.21922	5.8
**2a**	7	-33.9331	3.1400	6.2640	0.12517	3.3
**2a**	8	-33.5546	3.4000	5.5050	0.10755	2.8
**2a**	9	-33.0096	2.3350	3.5100	0.08644	2.3
**2a**	10	-33.0054	3.2000	7.2540	0.08630	2.3
**2a**	11	-32.8016	3.0480	5.5460	0.07952	2.1
**2a**	12	-32.6726	10.5770	12.1920	0.07552	2.0
**2a**	13	-32.1277	9.0450	10.9870	0.06070	1.6
**2a**	14	-31.9571	4.4260	7.1470	0.05669	1.5
**2a**	15	-31.9488	14.9530	17.9250	0.05650	1.5
**2a**	16	-31.3747	15.1630	18.0040	0.04488	1.2
**2a**	17	-31.2749	15.2870	17.9140	0.04312	1.1
**2a**	18	-31.2666	9.8760	11.4360	0.04298	1.1
**2a**	19	-31.1251	20.2450	23.1630	0.04061	1.1

**2a**	20	-31.1085	20.7430	23.4350	0.04034	1.1
**2a**	21	-30.8131	3.6620	8.0120	0.03583	0.9
**2a**	22	-30.7674	15.4180	18.4590	0.03518	0.9
**2a**	23	-30.5802	14.8080	17.3880	0.03264	0.9
**2a**	24	-30.2890	15.9290	18.8260	0.02904	0.8
**2a**	25	-30.0061	3.9490	7.4570	0.02593	0.7
**2a**	26	-29.9603	7.7510	10.1140	0.02546	0.7
**2a**	27	-29.9437	21.1550	23.1030	0.02529	0.7
**2a**	28	-29.9354	19.6070	22.3010	0.02520	0.7
**2a**	29	-29.8730	4.5740	7.3210	0.02458	0.7
**2a**	30	-29.3322	3.9960	8.3420	0.01979	0.5
**2b**	1	-43.7882	0.0000	0.0000	1.00000	35.7
**2b**	2	-41.5584	1.2600	2.3180	0.40905	14.6
**2b**	3	-40.3520	2.1000	4.3280	0.25219	9.0
**2b**	4	-39.5533	9.1980	12.2660	0.18309	6.5
**2b**	5	-37.8810	9.2240	11.7390	0.09365	3.3
**2b**	6	-37.4275	4.7790	8.9030	0.07808	2.8
**2b**	7	-37.3859	4.5990	9.5040	0.07679	2.7
**2b**	8	-37.1446	2.0640	3.0610	0.06971	2.5
**2b**	9	-37.1155	2.6920	4.3220	0.06890	2.5
**2b**	10	-36.9034	15.0450	17.5780	0.06328	2.3
**2b**	11	-36.8202	14.8090	17.7360	0.06121	2.2
**2b**	12	-36.5914	5.4650	10.9190	0.05584	2.0
**2b**	13	-35.8634	15.0030	17.4840	0.04171	1.5
**2b**	14	-35.7885	5.1390	8.2900	0.04047	1.4
**2b**	15	-35.5638	21.5270	23.8260	0.03699	1.3
**2b**	16	-35.1686	5.5240	10.7710	0.03157	1.1
**2b**	17	-34.8941	5.4650	7.9060	0.02828	1.0
**2b**	18	-34.7901	2.7150	5.9040	0.02712	1.0
**2b**	19	-34.5322	5.4560	10.3100	0.02446	0.9
**2b**	20	-33.8915	6.9240	11.3820	0.01892	0.7
**2b**	21	-33.8749	9.9840	12.0710	0.01879	0.7
**2b**	22	-33.6627	4.1920	7.5820	0.01726	0.6
**2b**	23	-33.6336	15.6730	18.1190	0.01706	0.6
**2b**	24	-33.4339	5.3940	9.9010	0.01575	0.6
**2b**	25	-33.3466	5.6820	9.9610	0.01521	0.5
**2b**	26	-32.8598	9.3150	11.9400	0.01251	0.4
**2b**	27	-32.8474	4.2020	7.8520	0.01245	0.4
**2b**	28	-32.7350	15.2230	17.4690	0.01190	0.4
**2b**	29	-32.6976	10.0550	12.0920	0.01172	0.4
**2b**	30	-32.2941	3.3380	7.7040	0.00997	0.4
**2c**	1	-41.3920	0.0000	0.0000	1.00000	20.3
**2c**	2	-41.3587	0.0340	3.1260	0.98675	20.0
**2c**	3	-39.3661	2.0660	5.2550	0.44388	9.0
**2c**	4	-39.3078	2.0590	4.2220	0.43363	8.8
**2c**	5	-37.2736	2.5590	4.8040	0.19184	3.9
**2c**	6	-36.9491	3.2520	6.5500	0.16844	3.4
**2c**	7	-36.6912	2.9770	5.3400	0.15189	3.1
**2c**	8	-35.9133	3.3200	6.1620	0.11120	2.3
**2c**	9	-35.8800	3.3130	5.9880	0.10972	2.2

**2c**	10	-35.5888	20.2560	24.1470	0.09763	2.0
**2c**	11	-35.4557	20.2350	24.2920	0.09256	1.9
**2c**	12	-35.4224	3.1560	6.7380	0.09133	1.9
**2c**	13	-35.2186	2.4970	4.9470	0.08416	1.7
**2c**	14	-35.1728	2.5040	5.6110	0.08263	1.7
**2c**	15	-35.1229	20.5280	24.5800	0.08100	1.6
**2c**	16	-34.8275	2.9910	6.1870	0.07195	1.5
**2c**	17	-34.7734	2.4410	5.3330	0.07041	1.4
**2c**	18	-34.7610	3.0210	5.5690	0.07006	1.4
**2c**	19	-34.4198	2.7380	4.7500	0.06110	1.2
**2c**	20	-34.1411	20.6690	24.8320	0.05464	1.1
**2c**	21	-34.0954	20.4900	24.3700	0.05365	1.1
**2c**	22	-34.0787	20.5110	24.2040	0.05329	1.1
**2c**	23	-33.9789	3.7010	6.6370	0.05120	1.0
**2c**	24	-33.8749	27.5440	30.6860	0.04911	1.0
**2c**	25	-33.8624	27.5020	30.6750	0.04887	1.0
**2c**	26	-33.8499	1.9990	3.9980	0.04862	1.0
**2c**	27	-33.7834	20.6690	24.8070	0.04734	1.0
**2c**	28	-33.7501	2.3820	4.5300	0.04671	0.9
**2c**	29	-33.5670	2.7480	6.6650	0.04341	0.9
**2c**	30	-33.2925	20.2700	24.1290	0.03888	0.8
**2d**	1	-50.6896	0.0000	0.0000	1.00000	39.8
**2d**	2	-50.5190	0.0740	2.9460	0.93391	37.2
**2d**	3	-44.4288	1.3650	2.6090	0.08127	3.2
**2d**	4	-42.6026	3.5700	7.4270	0.03908	1.6
**2d**	5	-42.5942	3.5310	7.3660	0.03895	1.5
**2d**	6	-42.4528	3.4870	7.0060	0.03680	1.5
**2d**	7	-42.4154	3.5170	7.6460	0.03625	1.4
**2d**	8	-42.4154	1.8000	3.9650	0.03625	1.4
**2d**	9	-42.2656	3.4820	9.4530	0.03414	1.4
**2d**	10	-42.2573	3.4810	9.8530	0.03403	1.4
**2d**	11	-42.0659	1.8020	4.8150	0.03151	1.3
**2d**	12	-41.4211	3.4950	9.3120	0.02434	1.0
**2d**	13	-40.2813	3.5620	8.5550	0.01541	0.6
**2d**	14	-40.2522	3.5710	8.9640	0.01523	0.6
**2d**	15	-40.2230	17.1270	20.1810	0.01505	0.6
**2d**	16	-40.2064	16.9990	20.3000	0.01495	0.6
**2d**	17	-40.0608	17.0240	20.4580	0.01411	0.6
**2d**	18	-39.9235	17.1930	20.2120	0.01335	0.5
**2d**	19	-39.8861	17.0540	20.3040	0.01315	0.5
**2d**	20	-39.8112	17.0350	20.1450	0.01276	0.5
**2d**	21	-39.1539	17.7800	20.8030	0.00981	0.4
**2d**	22	-38.9376	3.3290	9.1140	0.00899	0.4
**2d**	23	-38.8253	3.7150	9.6710	0.00860	0.3
**2d**	24	-38.5590	4.8120	7.9080	0.00773	0.3
**2d**	25	-38.4675	4.7760	7.7890	0.00745	0.3
**2d**	26	-38.4218	4.2820	8.2320	0.00731	0.3
**2d**	27	-38.4010	17.8270	20.9900	0.00725	0.3
**2d**	28	-38.2637	4.2710	8.0980	0.00686	0.3
**2d**	29	-37.2861	9.0160	11.1050	0.00464	0.2

**2d**	30	-37.0198	9.0870	11.3510	0.00417	0.2
**2e**	1	-47.0870	0.0000	0.0000	1.00000	32.6
**2e**	2	-46.2966	1.8850	4.0150	0.72842	23.7
**2e**	3	-45.1277	1.0180	2.4060	0.45588	14.8
**2e**	4	-42.5069	4.5260	10.5440	0.15942	5.2
**2e**	5	-41.4710	4.0400	6.4240	0.10524	3.4
**2e**	6	-41.4003	4.2900	9.7980	0.10230	3.3
**2e**	7	-40.8595	8.6010	11.8710	0.08236	2.7
**2e**	8	-40.2397	3.3710	8.7730	0.06424	2.1
**2e**	9	-40.0816	1.9660	3.7040	0.06029	2.0
**2e**	10	-38.9002	3.2780	8.4060	0.03755	1.2
**2e**	11	-38.6922	5.8720	9.5620	0.03454	1.1
**2e**	12	-38.0141	8.8090	12.4010	0.02632	0.9
**2e**	13	-37.8269	3.5360	6.3400	0.02442	0.8
**2e**	14	-37.5565	3.2040	8.3640	0.02191	0.7
**2e**	15	-37.5107	5.3980	11.5510	0.02151	0.7
**2e**	16	-37.2154	4.6820	7.8750	0.01911	0.6
**2e**	17	-37.1654	4.0840	8.0370	0.01873	0.6
**2e**	18	-36.8867	2.9980	7.8550	0.01675	0.5
**2e**	19	-36.2627	3.3820	8.3510	0.01304	0.4
**2e**	20	-36.0214	4.0560	7.4020	0.01184	0.4
**2e**	21	-35.4931	5.2770	11.6840	0.00958	0.3
**2e**	22	-35.1728	5.7120	9.0650	0.00843	0.3
**2e**	23	-35.0272	8.2270	10.8450	0.00795	0.3
**2e**	24	-34.8483	7.9200	10.7250	0.00740	0.2
**2e**	25	-34.4698	16.7240	19.1090	0.00636	0.2
**2e**	26	-34.2451	6.0860	9.4730	0.00581	0.2
**2e**	27	-34.1661	18.2920	20.7330	0.00563	0.2
**2e**	28	-34.0413	17.0920	19.5340	0.00535	0.2
**2e**	29	-33.9123	18.4400	20.9920	0.00508	0.2
**2e**	30	-33.8208	16.3460	19.1210	0.00490	0.2
**2f**	1	-36.2253	0.0000	0.0000	1.00000	17.8
**2f**	2	-35.2851	2.6050	4.7620	0.68597	12.2
**2f**	3	-34.2077	2.4000	4.8090	0.44536	7.9
**2f**	4	-34.0621	3.1780	6.2480	0.42011	7.5
**2f**	5	-33.4755	2.4720	4.5620	0.33207	5.9
**2f**	6	-33.2592	2.5510	4.8570	0.30449	5.4
**2f**	7	-32.6602	3.6450	7.0430	0.23948	4.3
**2f**	8	-32.3523	3.8710	5.7220	0.21168	3.8
**2f**	9	-31.7158	3.2960	6.5810	0.16400	2.9
**2f**	10	-31.3997	3.3370	6.2130	0.14448	2.6
**2f**	11	-31.3290	11.0170	12.2130	0.14044	2.5
**2f**	12	-31.1875	3.5450	5.4550	0.13270	2.4
**2f**	13	-31.0710	4.5820	7.1200	0.12664	2.3
**2f**	14	-31.0710	9.2300	10.9760	0.12664	2.3
**2f**	15	-30.6758	3.2060	5.4970	0.10809	1.9
**2f**	16	-30.4429	8.5910	10.8250	0.09845	1.8
**2f**	17	-30.3805	3.6430	6.1850	0.09602	1.7
**2f**	18	-30.3264	2.4990	3.7260	0.09396	1.7
**2f**	19	-30.2682	8.8910	10.7760	0.09179	1.6

**2f**	20	-29.9520	4.4270	7.2860	0.08086	1.4
**2f**	21	-29.9478	15.1600	17.8040	0.08073	1.4
**2f**	22	-29.3363	7.5540	9.8040	0.06318	1.1
**2f**	23	-29.2157	10.6140	11.4080	0.06019	1.1
**2f**	24	-29.1574	15.2520	17.9340	0.05880	1.0
**2f**	25	-29.0160	4.7210	7.4000	0.05556	1.0
**2f**	26	-28.9994	4.4980	8.1700	0.05519	1.0
**2f**	27	-28.9453	15.0080	17.3710	0.05401	1.0
**2f**	28	-28.9120	4.6970	7.5400	0.05329	0.9
**2f**	29	-28.6582	15.4160	18.0960	0.04814	0.9
**2f**	30	-28.5459	8.1750	10.2220	0.04602	0.8
**3a**	1	-45.9056	0.0000	0.0000	1.00000	24.8
**3a**	2	-44.6493	4.0850	6.8960	0.60431	15.0
**3a**	3	-43.9088	2.7430	6.7610	0.44909	11.1
**3a**	4	-43.6051	2.7330	7.3840	0.39761	9.9
**3a**	5	-43.4096	1.9650	6.9800	0.36763	9.1
**3a**	6	-43.1434	2.8490	8.0340	0.33042	8.2
**3a**	7	-41.9411	1.5640	2.1600	0.20405	5.1
**3a**	8	-40.2979	2.8040	4.9850	0.10559	2.6
**3a**	9	-39.8237	3.6120	9.5470	0.08731	2.2
**3a**	10	-39.6282	2.0360	4.7680	0.08073	2.0
**3a**	11	-39.5242	4.1940	8.6540	0.07743	1.9
**3a**	12	-37.6397	5.2790	9.4100	0.03638	0.9
**3a**	13	-36.9990	7.6660	12.0840	0.02814	0.7
**3a**	14	-36.9616	3.5170	8.3350	0.02772	0.7
**3a**	15	-36.2378	3.4760	5.4440	0.02074	0.5
**3a**	16	-36.1504	6.5350	9.5560	0.02002	0.5
**3a**	17	-36.0922	17.4050	22.5570	0.01956	0.5
**3a**	18	-36.0006	3.1170	8.7290	0.01886	0.5
**3a**	19	-35.8467	3.4760	8.1980	0.01773	0.4
**3a**	20	-35.8093	17.9830	22.1220	0.01746	0.4
**3a**	21	-35.7926	5.1480	9.6260	0.01735	0.4
**3a**	22	-35.7718	7.3630	10.8010	0.01720	0.4
**3a**	23	-35.3434	8.6610	10.2470	0.01449	0.4
**3a**	24	-35.1021	18.6380	22.9540	0.01315	0.3
**3a**	25	-35.0938	16.2500	21.5730	0.01311	0.3
**3a**	26	-34.7485	16.6970	22.1060	0.01141	0.3
**3a**	27	-34.3907	16.7820	20.8790	0.00989	0.2
**3a**	28	-34.3824	20.2040	23.9720	0.00986	0.2
**3a**	29	-33.6170	22.8680	25.7980	0.00725	0.2
**3a**	30	-33.5296	16.3960	21.5950	0.00700	0.2
**3b**	1	-50.1488	0.0000	0.0000	1.00000	43.9
**3b**	2	-46.6253	2.9100	8.4050	0.24351	10.7
**3b**	3	-46.4755	3.8880	10.0030	0.22932	10.1
**3b**	4	-45.3690	2.0790	7.5250	0.14715	6.5
**3b**	5	-44.1834	2.7510	8.1850	0.09148	4.0
**3b**	6	-43.7715	2.3540	7.6320	0.07756	3.4
**3b**	7	-43.7674	4.8700	11.0130	0.07743	3.4
**3b**	8	-43.6134	4.6030	9.5180	0.07280	3.2
**3b**	9	-43.2640	4.8150	10.7340	0.06328	2.8

**3b**	10	-43.1725	3.9220	10.2150	0.06100	2.7
**3b**	11	-42.0202	3.8200	7.6530	0.03843	1.7
**3b**	12	-41.3005	7.3210	10.3130	0.02880	1.3
**3b**	13	-40.2438	2.9230	8.4780	0.01886	0.8
**3b**	14	-40.1690	15.3980	19.3660	0.01830	0.8
**3b**	15	-39.4826	5.2410	10.1330	0.01390	0.6
**3b**	16	-39.2579	5.3900	9.3550	0.01270	0.6
**3b**	17	-38.5590	3.6740	7.7630	0.00960	0.4
**3b**	18	-38.1722	5.1170	10.8420	0.00822	0.4
**3b**	19	-38.0723	16.7250	21.6080	0.00789	0.3
**3b**	20	-37.8269	16.8240	20.7090	0.00715	0.3
**3b**	21	-37.5606	20.7230	24.6940	0.00643	0.3
**3b**	22	-37.5315	16.6080	21.0070	0.00636	0.3
**3b**	23	-37.5190	16.5880	20.4430	0.00632	0.3
**3b**	24	-37.1114	4.9980	8.9900	0.00537	0.2
**3b**	25	-36.9699	5.1150	9.4080	0.00507	0.2
**3b**	26	-36.7453	20.1440	24.1460	0.00464	0.2
**3b**	27	-36.3917	19.9060	22.8060	0.00402	0.2
**3b**	28	-36.3501	4.4760	8.9130	0.00396	0.2
**3b**	29	-36.1754	17.2330	21.8610	0.00369	0.2
**3b**	30	-35.7136	16.0480	19.9300	0.00307	0.1
**3c**	1	-40.1107	0.0000	0.0000	1.00000	14.5
**3c**	2	-40.0733	0.1280	5.6160	0.98510	14.3
**3c**	3	-40.0733	0.1030	3.0390	0.98510	14.3
**3c**	4	-40.0608	0.1000	4.7240	0.98019	14.2
**3c**	5	-36.6621	2.6180	5.1190	0.25093	3.6
**3c**	6	-36.5581	2.5910	5.3520	0.24068	3.5
**3c**	7	-36.3917	2.6040	4.7530	0.22515	3.3
**3c**	8	-36.1754	2.6300	5.5810	0.20645	3.0
**3c**	9	-35.4806	2.9530	6.0280	0.15626	2.3
**3c**	10	-35.4765	2.9310	6.2020	0.15600	2.3
**3c**	11	-35.3766	2.9740	5.2040	0.14988	2.2
**3c**	12	-35.1312	20.8810	24.5750	0.13583	2.0
**3c**	13	-35.0813	20.8910	24.7470	0.13314	1.9
**3c**	14	-35.0563	20.8610	24.9710	0.13182	1.9
**3c**	15	-35.0230	2.6420	5.6540	0.13007	1.9
**3c**	16	-35.0022	2.6520	5.0540	0.12899	1.9
**3c**	17	-34.9773	2.6370	4.9040	0.12770	1.9
**3c**	18	-34.9648	20.8700	24.8080	0.12707	1.8
**3c**	19	-33.2426	2.8840	6.7080	0.06370	0.9
**3c**	20	-33.1011	2.8760	7.0720	0.06019	0.9
**3c**	21	-33.0678	2.8840	5.9730	0.05939	0.9
**3c**	22	-32.9430	2.8820	6.3330	0.05650	0.8
**3c**	23	-32.8598	2.8430	7.0550	0.05464	0.8
**3c**	24	-32.8307	2.8270	6.3650	0.05401	0.8
**3c**	25	-32.8141	2.8390	6.0060	0.05365	0.8
**3c**	26	-32.7974	2.8450	7.3650	0.05329	0.8
**3c**	27	-32.4979	3.1650	6.0490	0.04726	0.7
**3c**	28	-32.3773	24.7720	29.0930	0.04503	0.7
**3c**	29	-32.2525	3.0770	6.0690	0.04283	0.6

**3c**	30	-32.1568	2.7740	5.7320	0.04122	0.6
**3d**	1	-46.8374	0.0000	0.0000	1.00000	32.0
**3d**	2	-43.4262	3.5970	7.2290	0.25472	8.1
**3d**	3	-43.2016	3.4150	7.2990	0.23279	7.4
**3d**	4	-43.1974	3.8480	9.9140	0.23240	7.4
**3d**	5	-42.5485	3.8250	10.0230	0.17916	5.7
**3d**	6	-42.2906	3.8200	7.5940	0.16156	5.2
**3d**	7	-41.7581	3.6720	10.3500	0.13050	4.2
**3d**	8	-40.7181	16.7550	20.4620	0.08601	2.8
**3d**	9	-40.6598	3.6160	9.7040	0.08402	2.7
**3d**	10	-40.5725	3.8420	9.2390	0.08113	2.6
**3d**	11	-40.5600	3.7340	9.9150	0.08073	2.6
**3d**	12	-40.4435	16.6470	20.1430	0.07704	2.5
**3d**	13	-39.8070	3.2440	5.7410	0.05969	1.9
**3d**	14	-39.7405	3.9620	10.4230	0.05812	1.9
**3d**	15	-39.4243	3.9050	9.0490	0.05120	1.6
**3d**	16	-39.2912	17.2120	20.9230	0.04854	1.6
**3d**	17	-38.5382	5.0880	8.0810	0.03589	1.1
**3d**	18	-38.5050	3.8040	9.4040	0.03542	1.1
**3d**	19	-38.3344	4.6290	8.5520	0.03308	1.1
**3d**	20	-37.7894	3.5760	9.8310	0.02658	0.9
**3d**	21	-37.4358	5.0770	9.0200	0.02307	0.7
**3d**	22	-37.1238	5.9430	8.6730	0.02036	0.7
**3d**	23	-37.1238	16.6600	20.3800	0.02036	0.7
**3d**	24	-37.0531	16.8350	20.4250	0.01979	0.6
**3d**	25	-37.0240	6.8410	10.7430	0.01956	0.6
**3d**	26	-36.9658	8.4480	14.0270	0.01911	0.6
**3d**	27	-36.8326	16.8880	20.5940	0.01812	0.6
**3d**	28	-36.5040	5.2420	9.3750	0.01588	0.5
**3d**	29	-35.8925	5.2230	9.3300	0.01243	0.4
**3d**	30	-34.8733	5.5790	8.9200	0.00826	0.3
**4c**	1	-42.9270	0.0000	0.0000	1.00000	19.3
**4c**	2	-42.8480	0.0540	4.2070	0.96881	18.7
**4c**	3	-42.7814	0.0730	5.2410	0.94330	18.3
**4c**	4	-42.7357	0.0760	3.1260	0.92615	17.9
**4c**	5	-38.3178	2.6150	5.2710	0.15757	3.0
**4c**	6	-38.2845	2.6360	4.6290	0.15548	3.0
**4c**	7	-38.1971	2.6360	5.1560	0.15013	2.9
**4c**	8	-38.1430	2.6240	4.7350	0.14691	2.8
**4c**	9	-35.9424	1.6680	5.4730	0.06080	1.2
**4c**	10	-35.3226	2.1190	3.8430	0.04742	0.9
**4c**	11	-35.2602	2.6000	5.6200	0.04625	0.9
**4c**	12	-35.2560	1.9290	5.6270	0.04617	0.9
**4c**	13	-34.9690	21.2340	24.6290	0.04115	0.8
**4c**	14	-34.9648	21.2180	24.6300	0.04108	0.8
**4c**	15	-34.9606	21.2410	24.8200	0.04102	0.8
**4c**	16	-34.8733	21.2140	24.4390	0.03960	0.8
**4c**	17	-34.6611	3.1560	7.1610	0.03638	0.7
**4c**	18	-34.6112	3.1550	6.6950	0.03565	0.7
**4c**	19	-34.5738	3.1430	6.4560	0.03512	0.7

**4c**	20	-34.5238	3.1680	5.9220	0.03443	0.7
**4c**	21	-33.9789	2.7030	4.7130	0.02767	0.5
**4c**	22	-33.8416	2.6350	4.9390	0.02619	0.5
**4c**	23	-33.6586	2.6840	5.4350	0.02434	0.5
**4c**	24	-33.3050	2.7360	5.0120	0.02112	0.4
**4c**	25	-33.2883	2.7350	5.6860	0.02098	0.4
**4c**	26	-33.1926	2.7290	4.9090	0.02019	0.4
**4c**	27	-33.1594	2.7380	5.5820	0.01992	0.4
**4c**	28	-32.9638	2.8350	4.9920	0.01842	0.4
**4c**	29	-32.9638	2.8610	4.7530	0.01842	0.4
**4c**	30	-32.8848	2.8580	5.0570	0.01785	0.3

3. Configuration of Autodock Vina used in the docking studies

3.1 For PRLm (protein structure extracted from PDB 2HQX)

receptor = 2qhx-prot.pdbqt
ligand = *<ligand_name>*.pdbqt
center_x = -5.178
center_y = 34.985
center_z = 55.300
size_x = 40.0
size_y = 40.0
size_z = 52.0
cpu = 4
exhaustiveness = 24
out = *<ligand_name>*-results.pdbqt
num_modes = 30
energy_range = 5

3.2 For PRTb (protein structure extracted from PDB 4CM7)

receptor = 4cm7-prot.pdbqt
ligand = *<ligand_name>*.pdbqt
center_x = 8.694
center_y = -5.023
center_z = 10.417
size_x = 40.0
size_y = 54.0
size_z = 48.0
cpu = 4
exhaustiveness = 24
out = *<ligand_name>*-results.pdbqt
num_modes = 30
energy_range = 5

4. Python Scripts

4.1. Script for finding the 10 closest contacts

```python
#! /usr/bin/env python3

# MIT License
#
#Copyright 2024 Filipe Teixeira
#
# Permission is hereby granted, free of charge, to any person obtaining a copy
# of this software and associated documentation files (the "Software"), to deal
# in the Software without restriction, including without limitation the rights
# to use, copy, modify, merge, publish, distribute, sublicense, and/or sell
# copies of the Software, and to permit persons to whom the Software is
# furnished to do so, subject to the following conditions:
#
# The above copyright notice and this permission notice shall be included in
# all copies or substantial portions of the Software.
#
# THE SOFTWARE IS PROVIDED "AS IS", WITHOUT WARRANTY OF ANY KIND, EXPRESS OR
# IMPLIED, INCLUDING BUT NOT LIMITED TO THE WARRANTIES OF MERCHANTABILITY,
# FITNESS FOR A PARTICULAR PURPOSE AND NONINFRINGEMENT. IN NO EVENT SHALL THE
# AUTHORS OR COPYRIGHT HOLDERS BE LIABLE FOR ANY CLAIM, DAMAGES OR OTHER
# LIABILITY, WHETHER IN AN ACTION OF CONTRACT, TORT OR OTHERWISE, ARISING FROM,
# OUT OF OR IN CONNECTION WITH THE SOFTWARE OR THE USE OR OTHER DEALINGS IN THE
# SOFTWARE.

__doc__=f"""
Usage: {__file__} [-h|--help] -r target.pdbqt [-o output.csv] ligand-file(s).pdbqt
"""

import numpy as np
import pandas as pd

def read_ref_pdbqt(fn):
    data=open(fn,'r').readlines()
    o=list()
    for line in data:
        if line.startswith('ATOM'):
            a=dict()
            a['ID']=int(line[8:12])
            a['name']=line[12:17].strip()
            a['resname']=line[17:20]
            a['chain']=line[21:22]
            a['resID']=int(line[22:26])
            a['pos']=np.array(list(map(float,[line[30:39],line[39:47],line[47:55]])))
            a['element']=line[77:80].strip()
            o.append(a)
    return o

def find_top_contacts(geo, ref, n=3):
    o=list()
    for i in range(n):
        o.append({'RefResName':'','RefResID':-1,'RefChain':'','RefAtomName':'',
                  'LigAtom':'','LigAtomID':-1,'Distance':1.0e10,
                  'Distance_to_B':0.0, 'Distance_to_C8':0.0, 'Orientation':0.0})
    cut = 1.0e10
    for a1 in geo:
        for a2 in ref:
            d = np.linalg.norm(a2['pos']-a1['pos'])
            if d < cut:
                for e in o:
                    if e['Distance'] > d:
                        e['RefResName']=a2['resname']
                        e['RefResID']=a2['resID']
                        e['RefChain']=a2['chain']
                        e['RefAtomName']=a2['name']
                        e['LigAtom']=a1['name']
                        e['LigAtomID']=a1['ID']
                        e['Distance']=d
                        e['Distance_to_B']=np.linalg.norm(a2['pos']-geo[8]['pos'])
                        e['Distance_to_C8']=np.linalg.norm(a2['pos']-geo[0]['pos'])
```

```
                        e['Orientation']=np.sign(e['Distance_to_C8']-e['Distance_to_B'])
                        break
                cut = max([x['Distance'] for x in o])
    return o

def analyse_ligand(fn, ref, temperature=300):
    R = 8.31446261815324e-3 # kJ/mol
    RT=R*temperature
    data=open(fn,'r').readlines()
    o=list()
    #Structure and close contacts
    n_models=1
    for line in data:
        if line.startswith('MODEL'):
            m=dict()
            m['Ligand']=fn[:-6]
            m['Mode']=n_models
            g=list()
        elif line.startswith('REMARK VINA RESULT:'):
            m['Affinity_kJmol']=4.16*float(line.split()[-3])
            m['rmsd_L']=float(line.split()[-2])
            m['rmsd_U']=float(line.split()[-1])
        elif line.startswith('ATOM') or line.startswith('HETATM'):
            a=dict()
            a['ID']=int(line[8:12])
            a['name']=line[12:17].strip()
            a['pos']=np.array(list(map(float,[line[30:39],line[39:47],line[47:55]])))
            a['element']=line[77:80].strip()
            g.append(a)
        elif line.startswith('ENDMDL'):
            contacts = find_top_contacts(g,ref, n=10)
            contacts.sort(key=lambda x: x['Distance'])
            n=1
            for l in contacts:
                for k,v in l.items():
                    m[f'C{n:d}_{k}']=v
                n += 1
            o.append(m)
            n_models += 1
    # Thermodynamics
    ref_BE = o[0]['Affinity_kJmol']
    sumZ=0.0
    for m in o:
        Z=np.exp(-(m['Affinity_kJmol']-ref_BE)/RT)
        m['Z']=Z
        sumZ += Z
    sumPop = 0.0
    for m in o:
        pop = 100.0 * (m['Z']/sumZ)
        m['%Pop'] = pop
        sumPop += pop
        m['%CPop'] = sumPop
    #o=pd.DataFrame(o)
    return o

def driver(ref_fn, ligs_fnl, ofn='output.csv'):
    ref=read_ref_pdbqt(ref_fn)
    all_data=list()
    for lfn in ligs_fnl:
        all_data += analyse_ligand(lfn, ref)
    final_data=pd.DataFrame(all_data)
    final_data.to_csv(ofn,index=False)

def main(args):
    opts={}
    opts['ref_fn']=''
    opts['ligs_fnl']=list()
    if(len(args)<2):
        print(__doc__)
        sys.exit(0)
    n=1
    while(n<len(args)):
        if(args[n]=='--help' or args[n]=='-h'):
            print(__doc__)
```

```
            sys.exit(0)
        elif(args[n]=='-r' or args[n]=='-r'):
            n += 1
            opts['ref_fn']=args[n]
        elif(args[n]=='-o' or args[n]=='-o'):
            n += 1
            opts['ofn']=args[n]
        else:
            opts['ligs_fnl'].append(args[n])
        n += 1
    if opts['ref_fn'] and opts['ligs_fnl']:
        driver(**opts)
    print("Goodbye now.")

if(__name__=='__main__'):
    import sys
    main(sys.argv)

```

4.2. Script for performing Principal Component Analysis on the affinity data

```
#! /usr/bin/env python3

# MIT License
#
#Copyright 2024 Filipe Teixeira
#
# Permission is hereby granted, free of charge, to any person obtaining a copy
# of this software and associated documentation files (the "Software"), to deal
# in the Software without restriction, including without limitation the rights
# to use, copy, modify, merge, publish, distribute, sublicense, and/or sell
# copies of the Software, and to permit persons to whom the Software is
# furnished to do so, subject to the following conditions:
#
# The above copyright notice and this permission notice shall be included in
# all copies or substantial portions of the Software.
#
# THE SOFTWARE IS PROVIDED "AS IS", WITHOUT WARRANTY OF ANY KIND, EXPRESS OR
# IMPLIED, INCLUDING BUT NOT LIMITED TO THE WARRANTIES OF MERCHANTABILITY,
# FITNESS FOR A PARTICULAR PURPOSE AND NONINFRINGEMENT. IN NO EVENT SHALL THE
# AUTHORS OR COPYRIGHT HOLDERS BE LIABLE FOR ANY CLAIM, DAMAGES OR OTHER
# LIABILITY, WHETHER IN AN ACTION OF CONTRACT, TORT OR OTHERWISE, ARISING FROM,
# OUT OF OR IN CONNECTION WITH THE SOFTWARE OR THE USE OR OTHER DEALINGS IN THE
# SOFTWARE.

__doc__=f"""
Usage: {__file__} [-h|--help] file.csv [-o basename]
"""

import pandas as pd
import matplotlib.pyplot as plt
import seaborn as sns
from sklearn import decomposition
from sklearn import preprocessing
from sklearn import manifold
from sklearn import inspection

def driver(ifn,ofn):
    data=pd.read_csv(ifn)
    # get list of named residues
    contact_count = 0
    for c_n in data.columns:
        if c_n.endswith('_RefResName'):
            contact_count += 1
    aa_lst = list()
    la_lst = list()
    for n in range(contact_count):
        l1=f'C{n+1}_RefResName'
        l2=f'C{n+1}_RefResID'
        l3=f'C{n+1}_RefChain'
        l4=f'C{n+1}_LigAtom'
```

```
        l5=f'C{n+1}_LigAtomID'
        for l in data.iterrows():
            s=f"{l[1][l1]}{l[1][l2]}({l[1][l3]})"
            if s not in aa_lst: aa_lst.append(s)
            sa=f"{l[1][l4]}({l[1][l5]})"
            if sa not in la_lst: la_lst.append(sa)
    ll = list(set(data['Ligand']))
    ll.sort()
    lll=[x.replace('-results','') for x in ll]
    aa_lst.sort(key=lambda x: int(x[3:-3]))
    la_lst.sort(key=lambda x: int(x[x.index('(')+1:-1]))
    # aa affinity per ligand
    res_aa=list()
    res_at=list()
    for lig in ll:
        o=dict()
        oa=dict()
        o['Ligand']=lig
        oa['Ligand']=lig
        for aan in aa_lst:
            o[aan]=0.0
        for a in la_lst:
            oa[a]=0.0
        d=data.loc[data['Ligand']==lig]
        for n in range(contact_count):
            l1=f'C{n+1}_RefResName'
            l2=f'C{n+1}_RefResID'
            l3=f'C{n+1}_RefChain'
            l4=f'C{n+1}_LigAtom'
            l5=f'C{n+1}_LigAtomID'
            l6=f'C{n+1}_Orientation'
            l7=f'C{n+1}_Distance'
            for l in d.iterrows():
                s=f"{l[1][l1]}{l[1][l2]}({l[1][l3]})"
                sa=f"{l[1][l4]}({l[1][l5]})"
                o[s] += l[1]['%Pop']
                oa[sa] += l[1]['%Pop']
        res_aa.append(o)
        res_at.append(oa)
    res_aa=pd.DataFrame(res_aa)
    res_aa.to_csv(ofn+'-aa_affinity.csv',index=False)
    res_at=pd.DataFrame(res_at)
    res_at.to_csv(ofn+'-atom_affinity.csv',index=False)
    # PCA
    n_comp=10
    pca = decomposition.PCA(n_components=n_comp)
    pc_lst=[f"PC{x+1}" for x in range(n_comp)]
    in_data=preprocessing.StandardScaler(with_std=False).fit_transform(res_aa[aa_lst])
    #scores=pca.fit_transform(in_data) # works well for 2qhx
    scores=pca.fit_transform(res_aa[aa_lst])
    scores=pd.DataFrame(scores, columns=pc_lst)
    scores['Ligand']=lll
    scores=scores.reindex(columns=['Ligand']+pc_lst)
    print(scores)
    loadings=pd.DataFrame(pca.components_,columns=aa_lst)
    loadings['PC']=pc_lst
    loadings['ExpVar']=100.0*pca.explained_variance_ratio_
    loadings = loadings.reindex(columns=['PC','ExpVar']+aa_lst)
    scores.to_csv(ofn+'_aa_pca_scores.csv', index=False)
    loadings.to_csv(ofn+'_aa_pca_loadings.csv', index=False)
    # plot PCA scores
    fig_size=(10,8)
    fig_dpi=96
    for i in range(n_comp):
        fig,ax=plt.subplots(figsize=fig_size,dpi=fig_dpi)
        ax.bar(scores['Ligand'],scores[f"PC{i+1}"])
        ax.set_xlabel(f"PC{i+1} ({100.0*pca.explained_variance_ratio_[i]:0.1f} %)")
        ax.set_ylabel(f"Score")
        fig.savefig(ofn+f'_aa_pca_scores{i+1:02d}.png')
        plt.clf()
        plt.close()

def main(args):
    opts={}
```

```
    opts['ifn']=''
    opts['ofn']='output'
    if(len(args)<2):
        print(__doc__)
        sys.exit(0)
    n=1
    while(n<len(args)):
        if(args[n]=='--help' or args[n]=='-h'):
            print(__doc__)
            sys.exit(0)
        elif(args[n]=='--output' or args[n]=='-o'):
            n += 1
            opts['ofn']=args[n]
        else:
            opts['ifn']=args[n]
        n += 1
    if opts['ifn']:
        driver(opts['ifn'],opts['ofn'])
    print("Goodbye now.")

if(__name__=='__main__'):
    import sys
    main(sys.argv)
```

5. References:

1. Cunha Dias De Rezende, L.; Menezes Vaidergorn, M.; Biazzotto Moraes, J.C.; Da Silva Emery, F. Synthesis, Photophysical Properties and Solvatochromism of *Meso*-Substituted Tetramethyl BODIPY Dyes. *J. Fluoresc.* **2014**, *24*, 257–266, doi:10.1007/s10895-013-1293-8.
2. Shi, W.-J.; Yan, X.-H.; Yang, J.; Wei, Y.-F.; Huo, Y.-T.; Su, C.-L.; Yan, J.; Han, D.; Niu, L. Development of *Meso* -Five-Membered Heterocycle BODIPY-Based AIE Fluorescent Probes for Dual-Organelle Viscosity Imaging. *Anal. Chem.* **2023**, *95*, 9646–9653, doi:10.1021/acs.analchem.3c01409.
3. Kukoyi, A.; He, H.; Wheeler, K. Quinoline-Functionalized BODIPY Dyes: Structural and Photophysical Properties. *J. Photochem. Photobiol. Chem.* **2022**, *425*, 113686, doi:10.1016/j.jphotochem.2021.113686.
